# Daily habits, diseases, drugs and knee osteoarthritis: a two-sample Mendelian randomization analysis

**DOI:** 10.3389/fgene.2024.1418551

**Published:** 2024-07-09

**Authors:** Yaqiong Zhou, Qi Wang, Liping Chen, Yun Bo, Yuanyuan Zhang

**Affiliations:** ^1^ Department of Orthopedics and Traumatology, Jiangsu Province Hospital of Chinese Medicine, Affiliated Hospital of Nanjing University of Chinese Medicine, Nanjing, China; ^2^ Department of Nursing, Jiangsu Province Hospital of Chinese Medicine, Affiliated Hospital of Nanjing University of Chinese Medicine, Nanjing, China

**Keywords:** Mendelian randomization, daily habits, diseases, drugs, knee osteoarthritis

## Abstract

**Background:**

The causal relationship between daily habits, diseases, drugs, and knee osteoarthritis (KOA) remains unclear. This study utilized a two-sample Mendelian randomization (MR) method to investigate the causal links between these factors and KOA, providing new insights for KOA prevention.

**Methods:**

SNPs strongly associated with exposure factors (daily habits, diseases, drugs) were extracted from publicly available genome-wide association study (GWAS) as instrumental variables (IVs). We then selected GWAS of KOA as the outcome, conducting a two-sample MR analysis.

**Results:**

Our findings revealed significant causal relationships between several factors and KOA. There was a notable association with time spent watching TV (OR = 4.038; 95% CI: 1.859–8.770; *P* = 4.192E-04), frequency of friend/family visits (OR = 0.415; 95% CI: 0.219–0.788; *P* = 7.174E-03), smoking history (OR = 0.781; 95% CI: 0.663–0.921; *P* = 3.235E-03), gastroesophageal reflux disease (GERD) (OR = 1.519; 95% CI: 1.244–1.856; *P* = 4.183E-05), hypercholesterolemia (OR = 0.498; 95% CI: 0.290–0.855; *P* = 0.011), hypothyroidism (OR = 1.048; 95% CI: 1.013–1.084; *P* = 6.645E-03), use of antithrombotic agents (OR = 0.892; 95% CI: 0.816–0.976; *P* = 0.013), statin medication (OR = 0.956; 95% CI: 0.916–0.998; *P* = 0.041), and thyroid preparations (OR = 1.042; 95% CI: 1.014–1.071; *P* = 2.974E-03) with KOA. Specifically, KOA was positively associated with longer time spent watching TV, GERD, hypothyroidism and thyroid preparations, however showed a negative correlation with more frequent visits from friends or family, smoking history, hypercholesterolemia, antithrombotic agents and statin medication. Sensitivity analysis indicated no significant pleiotropy in these studies (*P >* 0.05).

**Conclusion:**

This comprehensive study underscores the significance of modifying certain habits to mitigate the risk of KOA. Additionally, the elevated risk of KOA among individuals with GERD, hypothyroidism, and those using thyroid preparations warrants attention. These results would be beneficial for clinical research and nursing education.

## 1 Introduction

Knee osteoarthritis (KOA) is a common bone and joint disease, which is a knee joint disease characterized by joint pain, stiffness, and limited mobility, mainly caused by the degeneration of synovial joints (Shimizu et al., 2022). Mild symptoms may manifest as occasional joint stiffness and intermittent pain related to mobility, severe symptoms may manifest as persistent and severe pain and limited joint mobility, and more severe symptoms may include claudication and knee instability. The emergence of KOA seriously affects the quality of life of patients (Baez et al., 2023). With global aging on the rise, KOA’s prevalence is notably increasing, affecting more than one million individuals (Sharma, 2021). Some studies suggest that certain lifestyle habits, such as increased exercise, frequent toilet use, and wearing high heels, may elevate the risk of KOA to some extent (Mounach et al., 2008). However, most of these studies are observational and cannot ascertain a definitive causal relationship with KOA. Additionally, the association between diseases, medications, and KOA is susceptible to confounding factors, often leading to biased results and diminished credibility (Burgess et al., 2023).

Mendelian randomization (MR) analysis leverages data from genome-wide association study (GWAS), utilizing single nucleotide polymorphisms (SNPs) strongly correlated with exposure factors as instrumental variables (IVs) to infer causal effects between exposures and outcomes. As an effective statistical tool for causal inference in epidemiology, MR follows Mendelian laws of genetics, and the probability of being affected by confounding factors is almost zero. It effectively avoids the influence of subjective and objective factors such as environmental induction, human intervention, and reverse causality, and its results are stable and reliable. (Luo et al., 2021).

The objective of this study is to employ a two-sample MR method to scrutinize the causal relationship between select daily habits, diseases, medications, and KOA. By doing so, we aim to provide novel insights for KOA prevention and management.

## 2 Materials and methods

### 2.1 Ethical approval and informed consent

This study adheres to the STROBE-MR guidelines for MR studies (Supplementary Table S1). All original studies utilized in this research have obtained ethical approval and informed consent. Moreover, this study imposes no additional risks to the subjects and thus does not necessitate further signatures.

### 2.2 Study design

**FIGURE 1:**
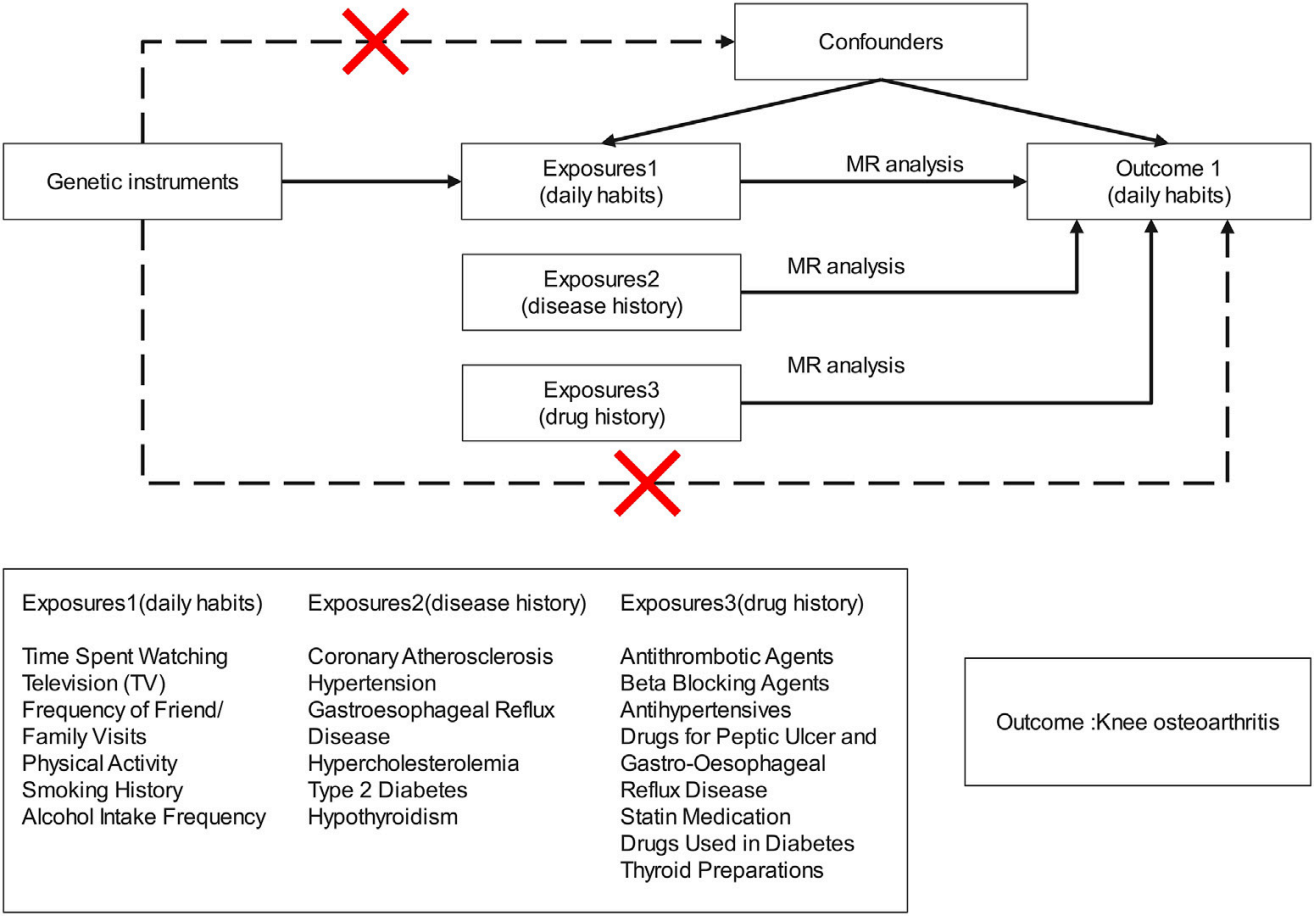
Design of this Mendelian randomization study. This study was performed with publicly available genome-wide association study (GWAS) datasets. MR, Mendelian randomization.

This study employs a two-sample MR method to explore the potential causal relationship between specific daily habits, disease history, drug history, and KOA. GWAS studies sourced from publicly available databases such as the MRC Integrative Epidemiology Unit (MRC-IEU), Neale Lab, and other independent research teams serve as reference datasets (Figure 1). Throughout the research process, MR analysis is guided by three critical hypotheses, namely, 1) correlation hypothesis: IVs are strongly correlated with exposure factors (*P* < 5E-08); 2) independence hypothesis: IVs are independent of the outcomes under investigation; and 3) hypothesis: IVs do not influence outcomes through pathways other than the intended exposure (Rui and Joseph, 2021).

### 2.3 Source of exposure factor data

In our clinical research, we have observed that lifestyle habits, disease history, and medication history have an impact on knee osteoarthritis, which is consistent with the observational study by Sharma (2021). However, in order to better reduce the interference of reverse causality and confounding factors, we chose Mendelian randomization analysis to illustrate these correlations. After fully considering the clinical relevance and the availability of GWAS data, we screened the exposure factor data for the study. These data encompass three main categories: daily habits, diseases, and corresponding drug usage history. Daily habits include variables such as time spent watching television (TV), frequency of friend/family visits, physical activity, smoking history, and alcohol intake frequency. Disease-related variables consist of coronary atherosclerosis (CAs), hypertension (HTN), gastroesophageal reflux disease (GERD), hypercholesterolemia, type 2 diabetes (T2DM), and hypothyroidism. Drug usage history includes categories such as antithrombotic agents, beta-blocking agents, antihypertensives, drugs for peptic ulcer and GERD, statin medication, drugs used in diabetes, and thyroid preparations. For this study, exposure factor data were sourced from publicly available GWAS studies conducted by institutions such as the MRC-IEU, Neale Lab, and other independent research teams. The characteristics of the GWAS summary data of exposures were shown in Table 1.

**TABLE 1:** Characteristics of the GWAS summary data of exposures.

Exposures/outcome	Consortium	Ethnicity	Sample size		PMID	Number of SNPs used as instrumental variable
			Cases	Controls		
Daily habits						
Time spent watching television (TV)	Neale Lab	European	319,740		—	6
Frequency of friend/family visits	MRC-IEU	European	459,830		—	7
Physical activity	Neale Lab	European	310,749		—	4
Smoking history	MRC-IEU	European	462,346		—	28
Alcohol Intake frequency	NA	European	89,683		—	50
Disease history						
Coronary atherosclerosis	NA	European	23,363	195,429	—	19
Hypertension	NA	European	55,917	162,837	—	53
Gastroesophageal reflux disease	NA	European	129,080	473,524	34187846	19
Hypercholesterolemia	Neale Lab	European	41,296	295,863	—	4
Type 2 diabetes	NA	European	48,286	250,671	29632382	30
Hypothyroidism	NA	European	30,155	379,986	34594039	61
Drug history						
Antithrombotic agents	NA	European	67,653	85,986	34594039	23
Beta Blocking agents	NA	European	31,700	192,324	34594039	48
Antihypertensives	NA	European	6,431	145,949	34594039	5
Drugs for peptic ulcer and gastro-oesophageal reflux disease	NA	European	53,137	79,230	34594039	3
Statin medication	NA	European	68,782	150,010	—	48
Drugs used in diabetes	NA	European	15,272	290,641	34594039	73
Thyroid preparations	NA	European	24,832	280,750	34594039	103
Outcome						
Knee osteoarthritis	NA	European	24,955	4,378,169	30664745	—

SNP, single nucleotide polymorphisms.

To construct IVs, genome-wide significant SNPs with a significance threshold of *P* < 5E-08 were extracted from the summary data of GWAS studies. Subsequently, SNPs with longer physical distances (≥5,000 kb) and a lower likelihood of linkage imbalance (*R* ^2^ < 0.01) were retained. To minimize weak instrumental bias, the exposure intensity of each SNP was assessed using the *F*-statistic (*F* = *β* ^2^/se^2^), where *β* represents the coefficient estimate and “se” represents the standard error. SNPs with an *F*-value > 10 were considered to have a strong correlation with the selected exposures. Furthermore, the National Institutes of Health (NIH) database (https://ldlink.nih.gov/) was consulted to identify SNPs directly associated with outcomes, along with recognized SNPs linked to risk factors for outcomes, such as knee trauma history, body mass index (Dong et al., 2023), education background (Haider et al., 2022), etc. (Supplementary Tables S5–S8). Finally, the SNP datasets of exposure factors and outcomes were merged, and incompatible alleles as well as SNPs from palindromes were removed (Supplementary Tables S9–S11). The remaining SNPs constitute the final IVs representing the exposure factors were shown in Table 1.

### 2.4 Source of outcome data

KOA was utilized as the primary outcome measure. The individuals participating in GWAS studies served as the subjects for exposure in the study. Specifically, the study included European individuals diagnosed with KOA, comprising 24,955 cases and 4,378,169 controls (Supplementary Table S12).

### 2.5 MR analysis

For the two-sample MR analysis, we employed the inverse variance weighting-fixed effects model (IVW-FE) as the primary method to assess the causal relationship among the aforementioned daily habits, disease history, drug history, and KOA. This analysis instrument ensures that outcomes are solely influenced by exposure factors and not through any other pathways (Burgess and Thompson, 2017). Additionally, four complementary MR analysis methods, namely, MR-Egger, weighted-median, weighted-model, and simple-model, were utilized to corroborate the findings. MR-Egger takes into account the presence of intercept terms and can detect and adjust for horizontal pleiotropy. If there is no horizontal pleiotropy, then the results of MR Egger regression and IVW are basically consistent (Dan et al., 2021). The premise of the weighted-median method is to provide accurate evaluation results based on the assumption that at least 50% of IVs are valid (Boehm et al., 2022). Weighted-model and simple-model are also two important statistical methods in MR analysis (Zhang et al., 2021). When the sample size used in the study is large enough, the Z-distribution method is used to calculate confidence intervals, while when the sample size is less than 30, the t-distribution method is used to calculate confidence intervals. All results are obtained in R language through code operations. Subsequently, Cochran’s *Q*-test was employed to evaluate potential heterogeneity (*P* < 0.05) (Bowden et al., 2019). In case of significant heterogeneity in the results (*P* < 0.05), we opted for the inverse variance weighted-random effects model (IVW-RE) as an alternative to IVW-FE. To verify the reliability of the results, we utilized the Egger-intercept method for multiple-effect testing, employing the intercept of MR-Egger to estimate the level of pleiotropy among genetic variants (*P* < 0.05 is considered significant) (Bowden et al., 2017). Furthermore, as a sensitivity analysis, the leave-one-out test was conducted to identify SNPs that are outliers by sequentially eliminating them one by one.

The entire MR study process was conducted using the TSMR software package (v0.5.8) in R (v4.2.1).

## 3 Results

### 3.1 Selection of IVs

Following the exclusion of linkage disequilibrium, screening by *F*-value, removal of confounding factors, and deletion of incompatible alleles, the remaining SNPs were included in the study (Supplementary Tables S13–S15).

### 3.2 Causal relationship between daily habits and KOA

**FIGURE 2:**
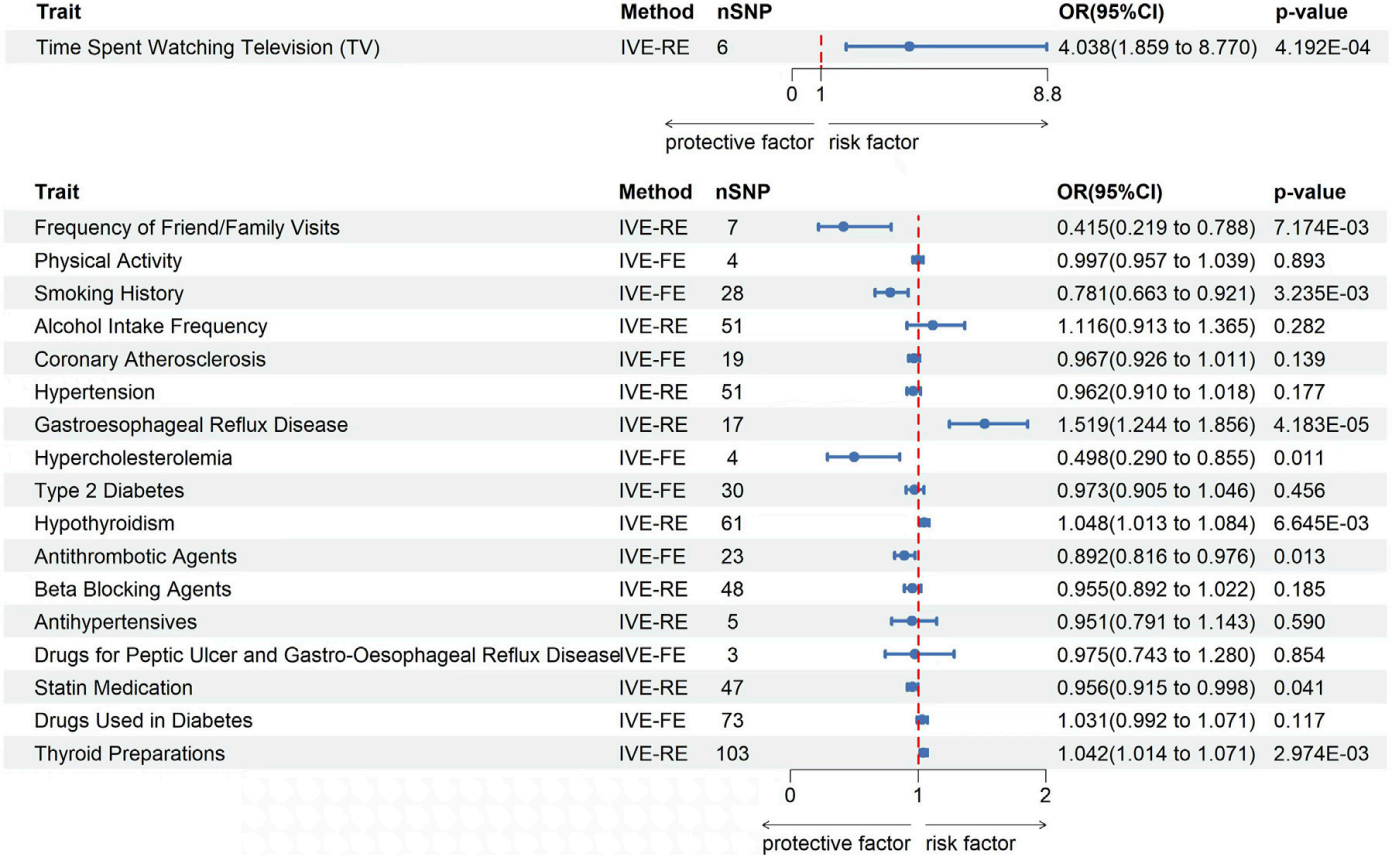
Forest plots of the results on two-sample Mendelian randomization analysis. OR, odds ratio. IVW-FE, inverse variance weighted-fixed effects model. IVW-RE, inverse variance weighted-random effects model.

**FIGURE 3:**
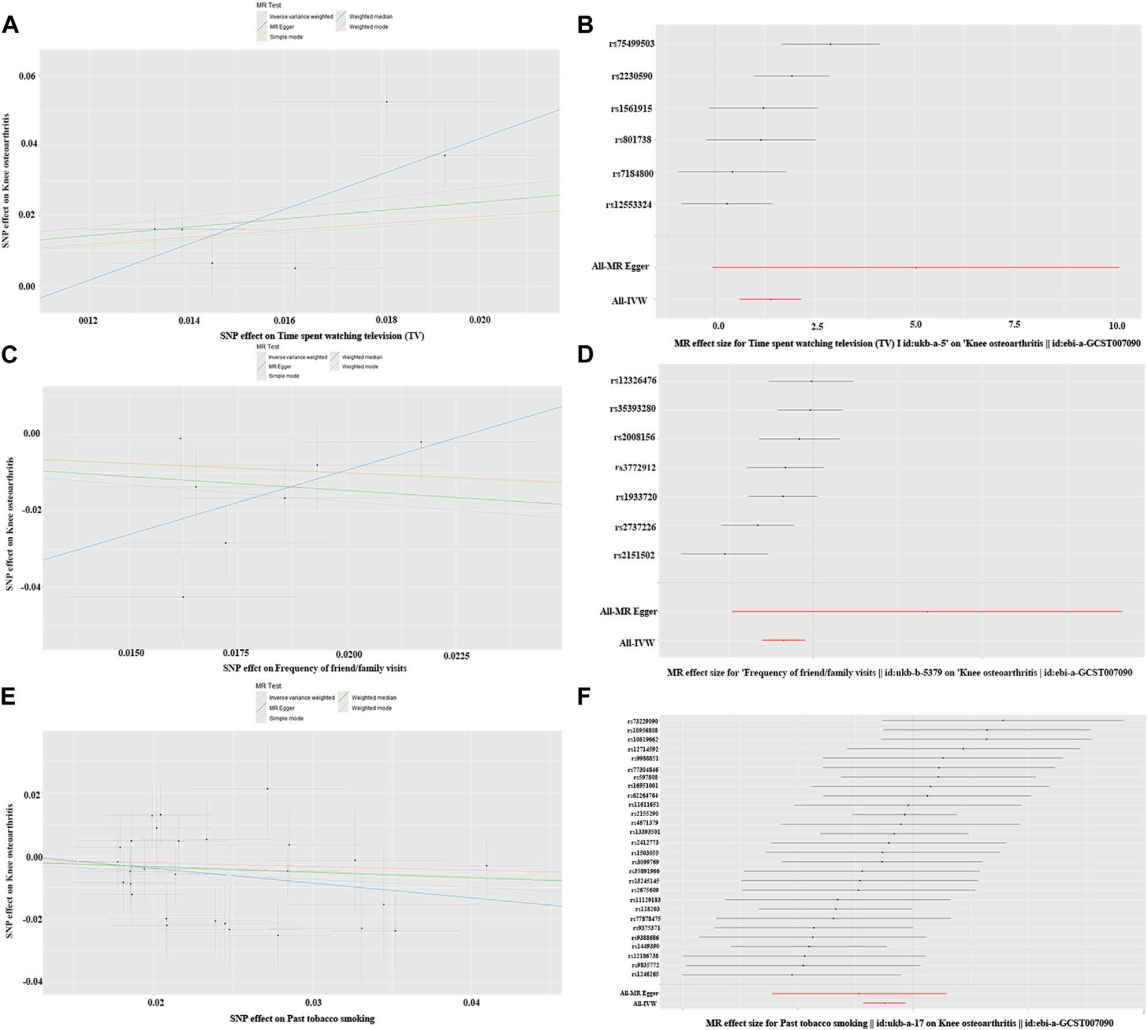
Part of visualization of Mendelian randomization analysis results about daily habits. **(A)** Scatter diagram (spent watching TV); **(B)** forest map (spent watching TV); A and B charts indicate KOA was positively associated with longer time spent watching TV and there is no obvious pleiotropy; **(C)** scatter diagram (frequency of friend/family visits); **(D)** forest map (frequency of friend/family visits); **(C, D)** charts indicate KOA was negatively correlation with more frequent visits from friends or family and there is no obvious pleiotropy; **(E)** scatter diagram (smoking history); **(F)** forest map (smoking history); **(E, F)** charts indicate KOA was negatively correlation with smoking history and there is no obvious pleiotropy.

Using a two-sample MR method, this study examined the causal relationship between five daily habits and KOA. The analysis revealed a significant causal association between time spent watching TV (OR = 4.038; 95% confidence interval [CI]: 1.859–8.770; *P* = 4.192E-04), frequency of friend/family visits (OR = 0.415; 95% CI: 0.219–0.788; *P* = 7.174E-03), and smoking history (OR = 0.781; 95% CI: 0.663–0.921; *P* = 3.235E-03) with KOA (Figure 2). KOA was positively associated with longer time spent watching TV, negatively associated with more frequent visits from friends or family and negatively associated with smoking history. Although some heterogeneity (*P* < 0.05) was observed between time spent watching TV, frequency of friend/family visits, alcohol Intake Frequency, and KOA, we did not observe any pleiotropy in these studies when verifying the stability of the results (*P* > 0.05) (Table 2). At last, the leave-one-out test further confirmed the robustness of these associations. After removing individual SNPs one by one, the effect values of the included instrumental variables remained similar to the overall effect values, indicating that there were no specific SNPs that had a significant impact on causal relationships (Supplementary Figures S1–S17). Part of visualization of Mendelian randomization analysis results about daily habits were shown in Figure 3.

**TABLE 2:** Pleiotropic analysis of daily habits, disease history and drug history on knee osteoarthritis risk.

Trait	Method	β	SE	Cochran’s Q test pval
Time spent watching television (TV)	IVW-RE	1.3958	0.3957	0.0283
Frequency of friend/family Visits	IVW-RE	−0.8788	0.3269	0.0327
Physical activity	IVW-FE	−0.0028	0.0212	0.0988
Smoking history	IVW-FE	−0.2472	0.0839	0.1497
Alcohol intake frequency	IVW-RE	0.1101	0.1024	7.00E-08
Coronary atherosclerosis	IVW-FE	−0.0332	0.0224	0.2595
Hypertension	IVW-RE	−0.0386	0.0286	0.0002
Gastroesophageal reflux disease	IVW-RE	0.4182	0.1021	0.0372
Hypercholesterolemia	IVW-FE	−0.6966	0.2755	0.4291
Type 2 diabetes	IVW-FE	−0.0276	0.037	0.252
Hypothyroidism	IVW-RE	0.0466	0.0172	0.0372
Antithrombotic agents	IVW-FE	−0.1141	0.0458	0.1194
Beta blocking agents	IVW-RE	−0.0462	0.0349	0.0146
Antihypertensives	IVW-RE	−0.0505	0.0939	0.0187
Drugs for peptic ulcer and gastro-oesophageal reflux disease	IVW-FE	−0.0255	0.1388	0.1186
Statin medication	IVW-RE	−0.0451	0.0221	0.0001
Drugs used in diabetes	IVW-FE	0.0305	0.0194	0.0815
Thyroid preparations	IVW-RE	0.0413	0.0139	0.0019

IVW-FE, inverse variance weighted-fixed effects model. IVW-RE, inverse variance weighted-random effects model.

### 3.3 Causal relationship between disease history and KOA

**FIGURE 4:**
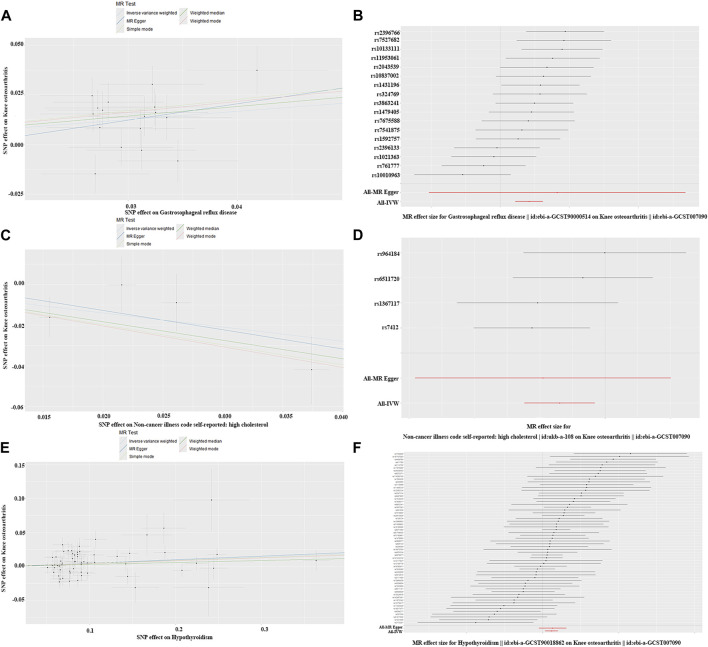
Part of visualization of Mendelian randomization analysis results about disease history. **(A)** scatter diagram (gastroesophageal reflux disease); **(B)** forest map (gastroesophageal reflux disease); **(A, B)** charts indicate KOA was positively associated with GERD and there is no obvious pleiotropy; **(C)** scatter diagram (hypercholesterolemia); **(D)** forest map (hypercholesterolemia); **(C, D)** charts indicate KOA was negatively correlation with hypercholesterolemia and there is no obvious pleiotropy; **(E)** scatter diagram (hypothyroidism); **(F)** forest map (hypothyroidism); **(E, F)** charts indicate KOA was positively correlation with hypothyroidism and there is no obvious pleiotropy.

We employed a two-sample MR method to analyze the causal relationship between six lifestyle habits and KOA. The study revealed a significant causal relationship between GERD (OR = 1.519; 95% CI: 1.244–1.856; *P* = 4.183E-05), hypercholesterolemia (OR = 0.498; 95% CI: 0.290–0.855; *P* = 0.011), and hypothyroidism (OR = 1.048; 95% CI: 1.013–1.084; *P* = 6.645E-03) with KOA (Figure 2). KOA was positively associated with GERD and hypothyroidism, however, showed a negative correlation with hypercholesterolemia. Although some heterogeneity (*P* < 0.05) was observed between HTN, GERD, hypothyroidism, and KOA, we did not observe any pleiotropy in these studies when verifying the stability of the results (*P* > 0.05) (Table 2). At last, the leave-one-out test further confirmed the robustness of these associations. After removing individual SNPs one by one, the effect values of the included instrumental variables remained similar to the overall effect values, indicating that there were no specific SNPs that had a significant impact on causal relationships (Supplementary Figures S1–S17). Part of visualization of Mendelian randomization analysis results about disease history were displayed in Figure 4.

### 3.4 Causal relationship between drugs history and KOA

**FIGURE 5:**
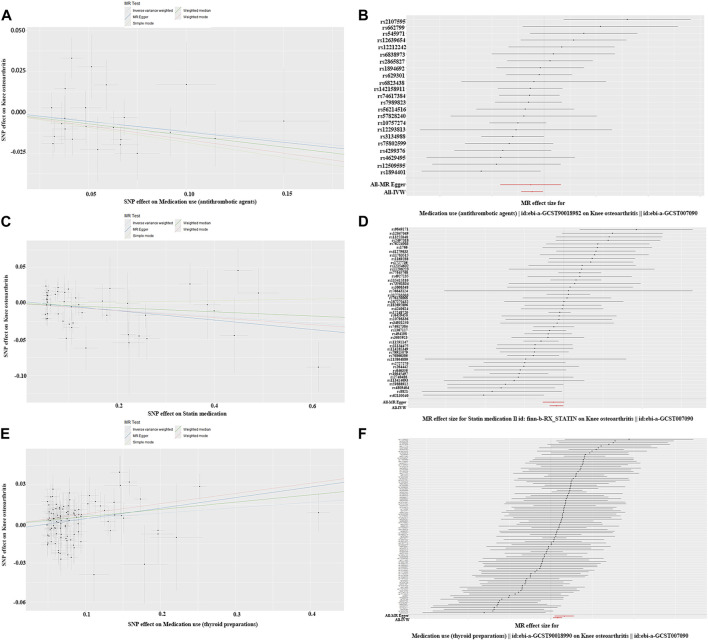
Part of visualization of Mendelian randomization analysis results about drugs history. **(A)** Scatter diagram (antithrombotic agents); **(B)** forest map (antithrombotic agents); **(A, B)** charts indicate KOA was negatively correlation with antithrombotic agents and there is no obvious pleiotropy; **(C)** scatter diagram (statin medication); **(D)** forest map (statin medication); **(C, D)** charts indicate KOA was negatively correlation with statin medication and there is no obvious pleiotropy; **(E)** scatter diagram (thyroid preparations); **(F)** forest map (thyroid preparations); **(E, F)** charts indicate KOA was positively correlation with thyroid preparations and there is no obvious pleiotropy.

This study used a two-sample MR method to analyze the causal relationship between seven related drugs and KOA. The study found a significant causal relationship between antithrombotic agents (OR = 0.892; 95% CI: 0.816–0.976; *P* = 0.013), statin medication (OR = 0.956; 95% CI: 0.916–0.998; *P* = 0.041), and thyroid preparations (OR = 1.042; 95% CI: 1.014–1.071; *P* = 2.974E-03) and KOA (Figure 2). KOA showed a negative association with antithrombotic agents and statin medication, but a positive associated with thyroid preparations. Although some heterogeneity (*P* < 0.05) was observed between beta-blocking agents, antihypertensives, statin medication, and thyroid preparations with KOA, we did not observe any pleiotropy in these studies when verifying the stability of the results (*P* > 0.05) (Table 2). At last, the leave-one-out test further confirmed the robustness of these associations. After removing individual SNPs one by one, the effect values of the included instrumental variables remained similar to the overall effect values, indicating that there were no specific SNPs that had a significant impact on causal relationships (Supplementary Figures S1–S17). Part of visualization of Mendelian randomization analysis results about drugs history were shown as in Figure 5.

## 4 Discussion

Old age, women, and obesity have gradually been recognized as risk factors for KOA (Andriacchi et al., 2015). Although the lifestyles of these groups share certain similarities, conventional observational studies struggle to establish a causal relationship between them and KOA. Thus, this study employed a two-sample MR method to explore the causal links between common daily habits and KOA. The results showed a significant positive causal association between time spent watching TV and KOA, alongside a significant negative causal association between the frequency of friend/family visits, smoking history, and KOA (Figure 3). Notably, physical activity and alcohol intake frequency showed no significant association with KOA. Based on these results, it is inferred that prolonged TV viewing increases the risk of KOA to some extent, while friend/family visits and a history of smoking are associated with a decreased risk of KOA.

The increase in time spent watching TV and the frequency of friend/family visits correspond to two distinct lifestyles: sedentary behavior and outdoor walking. Our research indicates a positive causal relationship between time spent watching TV and KOA (OR = 4.038; 95% CI: 1.859–8.770; *P* = 4.192E-04), whereas a negative causal relationship exists between the frequency of friend/family visits (OR = 0.415; 95% CI: 0.219–0.788; *P* = 7.174E-03) and KOA. These findings align with the research conducted by Wang et al. (2023), which suggested that moderate activity can help maintain leg muscle strength and stabilize joints, thereby reducing the risk of KOA. Hence, encouraging friend/family visits may contribute to minimizing the risk of KOA.

In this study, we investigated the association between smoking, alcohol consumption, and KOA. Our findings revealed a certain negative causal relationship between smoking history and KOA (OR = 0.781; 95% CI: 0.663–0.921; *P* = 3.235E-03). Numerous studies have indicated that nicotine in cigarettes can induce vasospasm, potentially exacerbating arthritis symptoms (Youngmee and Won-Kyung, 2018). However, Kong et al.’s (2017) study demonstrated that smokers have a reduced risk of KOA. While the underlying mechanism remains unclear, our MR study partially supports their findings. Additionally, our MR results indicate no causal relationship between alcohol intake frequency and KOA.

Previous research has suggested that increased physical activity poses a risk for KOA (Dong et al., 2023), yet our study found no significant causal relationship between physical activity and KOA. We speculate that this may be due to the lack of standardized physical activity measures, as certain activities not involving the knee joint may introduce bias into the results. Moreover, the exclusion of confounding factors in the MR study could also contribute to these findings. Further investigation using diverse exposure factor databases is warranted to clarify this discrepancy.

While a history of knee joint disease and arthritis has been extensively studied as risk factors for KOA (Bank et al., 2024), there is relatively limited research on the relationship between other systemic diseases and KOA. Therefore, we examined common diseases and corresponding medications as exposure factors to analyze their causal relationship with KOA. Our results indicate that GERD, hypothyroidism, and thyroid preparations have a positive causal relationship with KOA, whereas hypercholesterolemia, antithrombotic agents, and statin medication have a negative causal relationship with KOA (Figures 4, 5). In terms of the diseases and drugs we studied, no significant correlation was found between other factors and KOA.

The MR results clearly indicate that there is no direct relationship between CAs and KOA. Among the drugs taken post-diagnosis, antithrombotic agents exhibit a negative causal relationship with KOA (OR = 0.892; 95% CI: 0.816–0.976; *P* = 0.013), whereas beta-blocking agents do not show such an association. Inflammation and thrombosis exert bidirectional effects (Stark and Massberg, 2021), with the complement system playing a pivotal role. Thrombosis requires the participation of platelets, and platelet binding to complement amplifies the inflammatory response of immune cells (Peerschke et al., 2010). The utilization of antithrombotic agents attenuates this link to some extent, thereby diminishing the occurrence of inflammatory diseases. Although further discussion is warranted on the association between antithrombotic agents and other inflammatory conditions, this study furnishes evidence suggesting that antithrombotic agents may mitigate the risk of KOA. We posit that patients on long-term antithrombotic therapy may experience a reduction in the risk of developing KOA to some extent. It is noteworthy that the MR results indicate no direct causal relationship between CAs and KOA. We attribute this partly to the exclusion of confounding factors such as inflammation in the MR study, thereby eliminating the possibility of indirect relationships. Additionally, the lack of association between beta-blocking agents and KOA stems from their distinct therapeutic mechanisms compared to antithrombotic agents; beta-blocking agents primarily mitigate CAs by arteriole relaxation (Maltais et al., 2018).

Furthermore, we observed a positive relationship between GERD and KOA (OR = 1.519; 95% CI: 1.244–1.856; *P* = 4.183E-05), while medications for peptic ulcer and GERD did not show such an association. The esophageal mucosal components, dominated by inflammatory mediators, play a crucial role in the pathogenesis of GERD (Ustaoglu et al., 2020). The esophageal squamous cells exposed to acid reflux produce pro-inflammatory cytokines such as interleukin, which attract immune cells to the tissue, leading to an increase in inflammation levels in the human body (Souza et al., 2009). This mechanism can, to some extent, explain our findings. Based on the MR results, we advocate for regular health screenings for KOA following GERD diagnosis, which may reduce the risk of secondary KOA in patients to some extent.

Currently, there is no consensus regarding the relationship between high cholesterol and osteoarthritis. Recent research suggests that cholesterol 25-hydroxylase may inhibit the inflammatory response of chondrocytes by binding to the upstream target miR-*10a-3p*, thus potentially reducing the incidence of osteoarthritis (Li et al., 2021). Our findings support this notion (OR = 0.498; 95% CI: 0.290–0.855; *P* = 0.011). In addition, certain cholesterol metabolites, such as intestinal derived high-density lipoprotein, can block the inflammatory signals produced by intestinal bacteria, thereby avoiding inflammatory reactions and having significant anti-inflammatory effects. This viewpoint is consistent with our research findings (Han et al., 2021). While many observational studies have failed to establish a link between them (Abbas et al., 2022), MR analysis, being less susceptible to confounding factors, provides more reliable results to some extent. It is worth mentioning, that according to the MR results, we also observed a negative causal relationship between statin medication (OR = 0.956; 95% CI: 0.916–0.998; *P* = 0.041) and KOA. The mechanism by which statins reduce the incidence of KOA may be associated with the anti-inflammatory effect of elevated serum high-density lipoprotein and the pro-inflammatory effect of elevated serum low-density lipoprotein (LDL) and oxidized LDL (de Munter et al., 2016). However, further research is needed to elucidate the specific reasons. Based on our research findings, we believe that hypercholesterolemia and the use of statin medication can somewhat reduce the likelihood of KOA and delay the progression of the disease.

Thyroid hormones play an important role in growth, metabolism, and immunity. Our study identified a positive causal relationship between hypothyroidism, thyroid preparations, and KOA. We found that hypothyroidism may elevate the risk of KOA (OR = 1.048; 95% CI: 1.013–1.084; *P* = 6.645E-03), consistent with the findings of Ramendra et al. (2021). Our study revealed that patients with hypothyroidism exhibited significantly decreased adiponectin levels, contributing to various musculoskeletal dysfunctions. Furthermore, based on the MR results, we speculate that thyroid preparations are associated with an increased risk of KOA (OR = 1.042; 95% CI: 1.014–1.071; *P* = 2.974E-03), possibly due to their promotion of inflammatory cytokine secretion and induction of inflammation in the body (Wenzek et al., 2022). Although the specific mechanisms remain to be explored, MR analysis offers a theoretical basis. Thus, we posit that patients with hypothyroidism and those taking oral thyroid preparations should be mindful of the potential impact on KOA.

While there is a known correlation between HTN, T2DM, and inflammation (Wu et al., 2023), our study did not find a significant causal relationship between HTN, T2DM, or the use of related drugs and KOA. This may be attributed to the exclusion of confounding factors in the MR study or the limited availability of GWAS data.

Undoubtedly, our research has certain limitations. Firstly, although we adopted standardized methods for analysis and testing, there is significant heterogeneity in some associations, and the reliability of these results needs further investigation. Secondly, although some results are significant, the OR value is relatively small, and the relationship between these factors and KOA needs further verification. Finally, as the participants are of European descent, the results may not apply to other ethnic groups outside of Europe. We believe that as the GWAS database gradually improves, it is necessary to further validate this study in a more diverse population.

## 5 Conclusion

This study utilized a two-sample MR method to investigate the causal relationship between various daily habits, diseases, drugs, and KOA. Our comprehensive analysis revealed that time spent watching TV, GERD, hypothyroidism, and thyroid preparations exhibit a positive causal relationship with KOA. Conversely, the frequency of friend/family visits, smoking history, hypercholesterolemia, antithrombotic agents, and statin medication demonstrated a negative causal relationship with KOA.

We emphasize the significance of modifying certain habits to reduce the risk of KOA. Additionally, the heightened risk of KOA associated with GERD, hypothyroidism, and the use of thyroid preparations should not be overlooked, highlighting the importance of early prevention screening and the potential for targeted prevention strategies for individuals at risk of KOA. At last, we believe that these results would be beneficial for clinical research and nursing education.

## Data Availability

The original contributions presented in the study are included in the article/Supplementary Material, further inquiries can be directed to the corresponding authors.
