# Supplementary material for: Daily habits, diseases, drugs and knee osteoarthritis: a two-sample Mendelian randomization analysis
Source: Front Genet. 2024 Jul 9;15:1418551. doi: 10.3389/fgene.2024.1418551 (PMC11263196; doi:10.3389/fgene.2024.1418551)
Supplement: Supplementary file 1 [file DataSheet1.docx]

**Catalog**

[**Table S1. STROBE-MR checklist of Mendelian randomization study** 5](#_Toc162361468)

[**Table S2. Characteristics of the GWAS summary data of exposures (daily habits)** 11](#_Toc162361469)

[**Table S3. Characteristics of the GWAS summary data of exposures (disease history)** 12](#_Toc162361470)

[**Table S4. Characteristics of the GWAS summary data of exposures (drug history)** 13](#_Toc162361471)

[**Table S5. Basis for deleting confounding factors** 14](#_Toc162361472)

[**Table S6. Removed SNPs of daily habits and related traits (confounding factors)** 15](#_Toc162361473)

[**Table S7. Removed SNPs of disease history and related traits (confounding factors)** 19](#_Toc162361474)

[**Table S8. Removed SNPs of drug history and related traits (confounding factors)** 24](#_Toc162361475)

[**Table S9. Removed SNPs of daily habits (no confounding factors)** 29](#_Toc162361476)

[**Table S10. Removed SNPs of disease history (no confounding factors)** 30](#_Toc162361477)

[**Table S11. Removed SNPs of drug history (no confounding factors)** 31](#_Toc162361478)

[**Table S12. Characteristics of the GWAS summary data of outcome** 32](#_Toc162361479)

[**Table S13. SNPs used as instrumental variable for daily habits** 33](#_Toc162361480)

[**Table S14. SNPs used as instrumental variable for disease history** 38](#_Toc162361481)

[**Table S15. SNPs used as instrumental variable for drug history** 49](#_Toc162361482)

[**Table S16. Causal effects of daily habits on knee osteoarthritis risk using two sample mendelian randomization analysis** 73](#_Toc162361483)

[**Table S17. Causal effects of disease history on knee osteoarthritis risk using two sample mendelian randomization analysis** 76](#_Toc162361484)

[**Table S18. Causal effects of drug history on knee osteoarthritis risk using two sample mendelian randomization analysis** 80](#_Toc162361485)

[**Table S19. Sensitivity analyses (daily habits)** 84](#_Toc162361486)

[**Table S20. Sensitivity analyses (disease history)** 85](#_Toc162361487)

[**Table S21. Sensitivity analyses (drug history)** 86](#_Toc162361488)

[**Figure S1. Visualization of mendelian randomization analysis results about time spent watching television (exposure) - knee osteoarthritis (outcome)** 87](#_Toc162361489)

[**Figure S2. Visualization of mendelian randomization analysis results about frequency of friend/family visits (exposure) - knee osteoarthritis (outcome)** 88](#_Toc162361490)

[**Figure S3. Visualization of mendelian randomization analysis results about physical activity (exposure) - knee osteoarthritis (outcome)** 89](#_Toc162361491)

[**Figure S4. Visualization of mendelian randomization analysis results about smoking history (exposure) - knee osteoarthritis (outcome)** 90](#_Toc162361492)

[**Figure S5. Visualization of mendelian randomization analysis results about alcohol intake frequency (exposure) - knee osteoarthritis (outcome)** 91](#_Toc162361493)

[**Figure S6. Visualization of mendelian randomization analysis results about coronary atherosclerosis (exposure) - knee osteoarthritis (outcome)** 92](#_Toc162361494)

[**Figure S7. Visualization of mendelian randomization analysis results about hypertension (exposure) - knee osteoarthritis (outcome)** 93](#_Toc162361495)

[**Figure S8. Visualization of mendelian randomization analysis results about gastroesophageal reflux disease (exposure) - knee osteoarthritis (outcome)** 94](#_Toc162361496)

[**Figure S9. Visualization of mendelian randomization analysis results about hypercholesterolemia (exposure) - knee osteoarthritis (outcome)** 95](#_Toc162361497)

[**Figure S10. Visualization of mendelian randomization analysis results about type 2 diabetes (exposure) - knee osteoarthritis (outcome)** 96](#_Toc162361498)

[**Figure S11. Visualization of mendelian randomization analysis results about antithrombotic agents (exposure) - knee osteoarthritis (outcome)** 97](#_Toc162361499)

[**Figure S12. Visualization of mendelian randomization analysis results about beta blocking agents (exposure) - knee osteoarthritis (outcome)** 98](#_Toc162361500)

[**Figure S13. Visualization of mendelian randomization analysis results about antihypertensives (exposure) - knee osteoarthritis (outcome)** 99](#_Toc162361501)

[**Figure S14. Visualization of mendelian randomization analysis results about drugs for peptic ulcer and gastro-oesophageal reflux disease (exposure) - knee osteoarthritis (outcome)** 100](#_Toc162361502)

[**Figure S15. Visualization of mendelian randomization analysis results about statin medication (exposure) - knee osteoarthritis (outcome)** 101](#_Toc162361503)

[**Figure S16. Visualization of mendelian randomization analysis results about drugs used in diabetes (exposure) - knee osteoarthritis (outcome)** 102](#_Toc162361504)

[**Figure S17. Visualization of mendelian randomization analysis results about thyroid preparations (exposure) - knee osteoarthritis (outcome)** 103](#_Toc162361505)

| **Table.S1 STROBE-MR checklist of Mendelian randomization study** | | | |
| --- | --- | --- | --- |
| Item No. | Section | Checklist item | Page No. |
| 1 | TITLE and ABSTRACT | Indicate Mendelian randomization (MR) as the study’s design in the title and/or the abstract if that is a main purpose of the study | Page 1 |
|  | INTRODUCTION |  |  |
| 2 | Background | Explain the scientific background and rationale for the reported study. What is the exposure? Is a potential causal relationship between exposure and outcome plausible? Justify why MR is a helpful method to address the study question | Page 1-2 |
| 3 | Objectives | State specific objectives clearly, including pre-specified causal hypotheses (if any). State that MR is a method that, under specific assumptions, intends to estimate causal effects | Page 1-3 |
|  | METHODS |  |  |
| 4 | Study design and data sources | Present key elements of the study design early in the article. Consider including a table listing sources of data for all phases of the study. For each data source contributing to the analysis, describe the following: | Page 2-3 |
|  | a) | Setting: Describe the study design and the underlying population, if possible. Describe the setting, locations, and relevant dates, including periods of recruitment, exposure, follow-up, and data collection, when available. | Not mentioned |
|  | b) | Participants: Give the eligibility criteria, and the sources and methods of selection of participants. Report the sample size, and whether any power or sample size calculations were carried out prior to the main analysis | Page 2-3 |
|  | c) | Describe measurement, quality control and selection of genetic variants | Page 2-3 |
|  | d) | For each exposure, outcome, and other relevant variables, describe methods of assessment and diagnostic criteria for diseases | Page 2-3 |
|  | e) | Provide details of ethics committee approval and participant informed consent, if relevant | Page 2 |
| 5 | Assumptions | Explicitly state the three core IV assumptions for the main analysis (relevance, independence and exclusion restriction) as well assumptions for any additional or sensitivity analysis | Page 3 |
| 6 | Statistical methods: main analysis | Describe statistical methods and statistics used |  |
|  | a) | Describe how quantitative variables were handled in the analyses (i.e., scale, units, model) | Page 3-4 |
|  | b) | Describe how genetic variants were handled in the analyses and, if applicable, how their weights were selected | Page 3-4 |
|  | c) | Describe the MR estimator (e.g. two-stage least squares, Wald ratio) and related statistics. Detail the included covariates and, in case of two-sample MR, whether the same covariate set was used for adjustment in the two samples | Page 3-4 |
|  | d) | Explain how missing data were addressed | Not mentioned |
|  | e) | If applicable, indicate how multiple testing was addressed | Not mentioned |
| 7 | Assessment of assumptions | Describe any methods or prior knowledge used to assess the assumptions or justify their validity | Page 4 |
| 8 | Sensitivity analyses and additional analyses | Describe any sensitivity analyses or additional analyses performed (e.g. comparison of effect estimates from different approaches, independent replication, bias analytic techniques, validation of instruments, simulations) | Page 4 |
| 9 | Software and pre- registration |  |  |
|  | a) | Name statistical software and package(s), including version and settings used | Page 4 |
|  | b) | State whether the study protocol and details were pre-registered (as well as when and where) | Not mentioned |
|  | RESULTS |  |  |
| 10 | Descriptive data |  |  |
|  | a) | Report the numbers of individuals at each stage of included studies and reasons for exclusion. Consider use of a flow diagram | Figure 1 |
|  | b) | Report summary statistics for phenotypic exposure(s), outcome(s), and other relevant variables (e.g. means, SDs, proportions) | Page 4-5 |
|  | c) | If the data sources include meta-analyses of previous studies, provide the assessments of heterogeneity across these studies | Not mentioned |
|  | d) | For two-sample MR: i. Provide justification of the similarity of the genetic variant-exposure associations between the exposure and outcome samples | Page 3-5 |
|  |  | ii. Provide information on the number of individuals who overlap between the exposure and outcome studies | Page 3-5 |
| 11 | Main results |  |  |
|  | a) | Report the associations between genetic variant and exposure, and between genetic variant and outcome, preferably on an interpretable scale | Page 4-5 |
|  | b) | Report MR estimates of the relationship between exposure and outcome, and the measures of uncertainty from the MR analysis, on an interpretable scale, such as odds ratio or relative risk per SD difference | Table 1 |
|  | c) | If relevant, consider translating estimates of relative risk into absolute risk for a meaningful time period | Not mentioned |
|  | d) | Consider plots to visualize results (e.g. forest plot, scatterplot of associations between genetic variants and outcome versus between genetic variants and exposure) | Additional file : Figure 1-17 |
| 12 | Assessment of assumptions |  |  |
|  | a) | Report the assessment of the validity of the assumptions | Page 4-5 |
|  | b) | Report any additional statistics (e.g., assessments of heterogeneity across genetic variants, such as I2, Q statistic or E-value) | Page 4-5 |
| 13 | Sensitivity analyses and additional analyses |  |  |
|  | a) | Report any sensitivity analyses to assess the robustness of the main results to violations of the assumptions | Page 4-5 |
|  | b) | Report results from other sensitivity analyses or additional analyses | Page 4-5 |
|  | c) | Report any assessment of direction of causal relationship (e.g., bidirectional MR) | Not mentioned |
|  | d) | When relevant, report and compare with estimates from non-MR analyses | Not mentioned |
|  | e) | Consider additional plots to visualize results (e.g., leave-one-out analyses) | Not mentioned |
|  | DISCUSSION |  |  |
| 14 | Key results | Summarize key results with reference to study objectives | Page 4-5 |
| 15 | Limitations | Discuss limitations of the study, taking into account the validity of the IV assumptions, other sources of potential bias, and imprecision. Discuss both direction and magnitude of any potential bias and any efforts to address them | Page 5-9 |
| 16 | Interpretation |  |  |
|  | a) | Meaning: Give a cautious overall interpretation of results in the context of their limitations and in comparison with other studies | Page 5-9 |
|  | b) | Mechanism: Discuss underlying biological mechanisms that could drive a potential causal relationship between the investigated exposure and the outcome, and whether the gene-environment equivalence assumption is reasonable. Use causal language carefully, clarifying that IV estimates may provide causal effects only under certain assumptions | Page 5-9 |
|  | c) | Clinical relevance: Discuss whether the results have clinical or public policy relevance, and to what extent they inform effect sizes of possible interventions | Page 5-9 |
| 17 | Generalizability | Discuss the generalizability of the study results (a) to other populations, (b) across other exposure periods/timings, and (c) across other levels of exposure | Page 9-10 |
|  | OTHER INFORMATION |  |  |
| 18 | Funding | Describe sources of funding and the role of funders in the present study and, if applicable, sources of funding for the databases and original study or studies on which the present study is based | Title page |
| 19 | Data and data sharing | Provide the data used to perform all analyses or report where and how the data can be accessed, and reference these sources in the article. Provide the statistical code needed to reproduce the results in the article, or report whether the code is publicly accessible and if so, where | Page 2-4 |
| 20 | Conflicts of Interest | All authors should declare all potential conflicts of interest | Conflict of Interest |

| Table S2. Characteristics of the GWAS summary data of exposures (daily habits) | | | | | | |
| --- | --- | --- | --- | --- | --- | --- |
| Exposures | Consortiums | Ethnicity | Sample size | | PMID | SNPs |
|  |  |  | cases | controls |  |  |
| Time Spent Watching Television (TV) | Neale Lab | European | 319,740 | | —— | 10,894,596 |
| Frequency of Friend/Family Visits | MRC-IEU | European | 459,830 | | —— | 9,851,867 |
| Physical Activity | Neale Lab | European | 310,749 | | —— | 10,894,596 |
| Smoking History | MRC-IEU | European | 462,346 | | —— | 9,851,867 |
| Alcohol Intake Frequency | NA | European | 89,683 | | —— | 8,669,219 |
| SNP, single nucleotide polymorphisms. | | | | | | |

| **Table S3. Characteristics of the GWAS summary data of exposures (disease history)** | | | | | | |
| --- | --- | --- | --- | --- | --- | --- |
| Exposures | Consortiums | Ethnicity | Sample size | | PMID | SNPs |
|  |  |  | cases | controls |  |  |
| Coronary Atherosclerosis | NA | European | 23,363 | 195,429 | —— | 16,380,466 |
| Hypertension | NA | European | 55,917 | 162,837 | —— | 16,380,466 |
| Gastroesophageal Reflux Disease | NA | European | 129,080 | 473,524 | 34187846 | 2,320,781 |
| Hypercholesterolemia | Neale Lab | European | 41,296 | 295,863 | —— | 10,894,596 |
| Type 2 Diabetes | NA | European | 48,286 | 250,671 | 29632382 | 190,486 |
| Hypothyroidism | NA | European | 30,155 | 379,986 | 34594039 | 24,138,872 |
| SNP, single nucleotide polymorphisms. |  |  |  |  |  |  |

| **Table S4. Characteristics of the GWAS summary data of exposures (drug history)** | | | | | | |
| --- | --- | --- | --- | --- | --- | --- |
| Exposures | Consortiums | Ethnicity | Sample size | | PMID | SNPs |
|  |  |  | cases | controls |  |  |
| Antithrombotic Agents | NA | European | 67,653 | 85,986 | 34594039 | 14,241,708 |
| Beta Blocking Agents | NA | European | 31,700 | 192,324 | 34594039 | 14,250,787 |
| Antihypertensives | NA | European | 6,431 | 145,949 | 34594039 | 14,265,107 |
| Drugs for Peptic Ulcer and Gastro-Oesophageal Reflux Disease | NA | European | 53,137 | 79,230 | 34594039 | 14,242,640 |
| Statin Medication | NA | European | 68,782 | 150,010 | —— | 16,380,466 |
| Drugs Used in Diabetes | NA | European | 15,272 | 290,641 | 34594039 | 14,254,974 |
| Thyroid Preparations | NA | European | 24,832 | 280,750 | 34594039 | 14,259,552 |
| SNP, single nucleotide polymorphisms. |  |  |  |  |  |  |

| **Table S5. Basis for deleting confounding factors** | | | |
| --- | --- | --- | --- |
| Order Number | Article Title | PMID | Influence Factor |
| 1 | Evidence on risk factors for knee osteoarthritis in middle-older aged: a systematic review and meta analysis | 37641050 | trauma history in knee, body mass index (BMI) ≥ 24 kg/m2, gender (female), age ≥ 40, more exercise, a high school education background, an university education background |
| 2 | Risk factors of knee osteoarthritis: A case-control study | 31258567 | body mass index, lodging (living in highland vs. plain), type of used toilet (regular vs. toilet), history of using high-heeled shoes (>3 cm) , history of knee Injury, history of lower limb fracture, Number of pregnanciesand history of pain, swelling (lasting for one months) |
| 3 | Modifiable risk factors in knee osteoarthritis: treatment implications | 30911813 | living in highland area, using regular toilet, having knee injury, lower limb fracture in the past |
| 4 | Risk factors of knee osteoarthritis in Bangladeshi adults: a national survey | 35395747 | increasing age, low educational level , overweight |
| 5 | A systems view of risk factors for knee osteoarthritis reveals insights into the pathogenesis of the disease | 25224078 | aging, obesity, joint trauma |

| **Table S6. Removed SNPs of daily habits and related traits (confounding factors)** | | | |
| --- | --- | --- | --- |
| Exposures | SNPs | PMID | traits |
| Time Spent Watching Television (TV) | rs62199883 | 30038396 | Educational attainment (years of education) |
|  | rs10189857 | 34855049 | Educational attainment |
| Frequency of Friend/Family Visits | rs12044657 | 35361970 | Educational attainment |
|  | rs862999 | 25201988 | Educational attainment |
|  | rs74967575 | 26426971 | Body mass index |
|  | rs1441098 | 35361970 | Educational attainment |
|  | rs35497369 | 35361970 | Educational attainment |
|  | rs2410941 | 36581621 | Body mass index |
|  | rs2179517 | 36581621 | Body mass index |
|  | rs17614190 | 25673413 | Body mass index |
| Physical Activity | rs34517439 | 25673413 | Body mass index |
| Smoking History | rs2613499 | 25673413 | Body mass index |
|  | rs528301 | 35361970 | Educational attainment |
|  | rs1558957 | 30038396 | Educational attainment |
|  | rs4233974 | 30239722 | Body mass index |
|  | rs67402191 | 36581621 | Body mass index |
|  | rs986391 | 31669095 | Body mass index |
|  | rs12529130 | 34021172 | Body mass index |
|  | rs9381919 | 31669095 | Body mass index |
|  | rs13237637 | 30038396 | Educational attainment |
|  | rs7870475 | 28892062 | Body mass index |
|  | rs12244388 | 30239722 | Body mass index |
|  | rs6265 | 28892062 | Body mass index |
|  | rs8034783 | 30595370 | Educational attainment |
|  | rs7216173 | 35361970 | Educational attainment |
|  | rs76608582 | 30595370 | Educational attainment |
| Alcohol Intake Frequency | rs780569 | 30038396 | Educational attainment (years of education) |
|  | rs2244598 | 30595370 | Educational attainment (years of education) |
|  | rs7514579 | 30038396 | Educational attainment (years of education) |
|  | rs780094 | 30239722 | Body mass index |
|  | rs10188314 | 36581621 | Body mass index |
|  | rs17662759 | 30038396 | Educational attainment (MTAG) |
|  | rs76082653 | 36581621 | Body mass index |
|  | rs7610856 | 30038396 | Educational attainment (MTAG) |
|  | rs1228589 | 26426971 | Body mass index |
|  | rs13135092 | 25673413 | Body mass index |
|  | rs362307 | 30038396 | Educational attainment (years of education) |
|  | rs1229984 | 28892062 | Body mass index |
|  | rs62339673 | 35361970 | Educational attainment |
|  | rs34811474 | 20966902 | Weight |
|  | rs4916723 | 31669095 | Body mass index |
|  | rs56194430 | 35361970 | Educational attainment |
|  | rs9403297 | 36581621 | Body mass index |
|  | rs12153855 | 30038396 | Educational attainment (MTAG) |
|  | rs9372625 | 27225129 | Educational attainment (years of education) |
|  | rs73050128 | 35361970 | Educational attainment |
|  | rs9648478 | 30038396 | Educational attainment (years of education) |
|  | rs11787216 | 30595370 | Body mass index |
|  | rs2977454 | 30038396 | Educational attainment (MTAG) |
|  | rs489062 | 35361970 | Educational attainment |
|  | rs61873510 | 31669095 | Body mass index |
|  | rs4242715 | 30595370 | Body mass index |
|  | rs11223617 | 35361970 | Educational attainment |
|  | rs1666658 | 35361970 | Educational attainment |
|  | rs7302200 | 35361970 | Educational attainment |
|  | rs28768122 | 30038396 | Educational attainment (years of education) |
|  | rs58905411 | 35361970 | Educational attainment |
|  | rs2535911 | 33414549 | Cognitive aspects of educational attainment |
|  | rs117799466 | 30038396 | Educational attainment (years of education) |
|  | rs34631026 | 35361970 | Educational attainment |
|  | rs72787062 | 35361970 | Educational attainment |
|  | rs35105141 | 38116116 | Body mass index |
|  | rs1421085 | 26961502 | Body mass index |
|  | rs8043563 | 28552196 | Body mass index |
|  | rs2411453 | 30595370 | Educational attainment (years of education) |
|  | rs9906502 | 35361970 | Educational attainment |
|  | rs8614 | 35361970 | Educational attainment |
|  | rs650558 | 30595370 | Body mass index |
|  | rs5022348 | 30038396 | Educational attainment (years of education) |
|  | rs4940926 | 23669352 | Body mass index |
|  | rs11700855 | 35361970 | Educational attainment |
|  | rs1894544 | 37106081 | BMI (standard GWA) |

| **Table S7. Removed SNPs of disease history and related traits (confounding factors)** | | | |
| --- | --- | --- | --- |
| Exposures | SNPs | PMID | traits |
| Coronary Atherosclerosis | rs629301 | 37844115 | Pain (pleiotropy) |
|  | rs72664324 | 36581621 | Body mass index |
|  | rs9349379 | 37844115 | Pain (pleiotropy) |
|  | rs964184 | 23251661 | Obesity-related traits |
|  | rs7412 | 30038396 | Educational attainment (years of education) |
| Hypertension | rs10206833 | 30038396 | Educational attainment |
|  | rs2190994 | 35361970 | Educational attainment |
|  | rs62455829 | 30595370 | Body mass index |
|  | rs557675 | 35361970 | Educational attainment |
|  | rs303949 | 35361970 | Educational attainment |
|  | rs28567725 | 28448500 | Body mass index |
| Gastroesophageal Reflux Disease | rs1011407 | 36581621 | Body mass index |
|  | rs11762636 | 30038396 | Educational attainment (years of education) |
|  | rs12204714 | 34855049 | Educational attainment |
|  | rs12357321 | 30108127 | Body mass index |
|  | rs12453010 | 35361970 | Educational attainment |
|  | rs12967855 | 35361970 | Educational attainment |
|  | rs12997558 | 36376304 | Body mass index |
|  | rs13107325 | 25673413 | Body mass index |
|  | rs1334297 | 34855049 | Educational attainment |
|  | rs1510719 | 27225129 | Educational attainment (years of education) |
|  | rs1596747 | 30595370 | Body mass index |
|  | rs1883842 | 30038396 | Educational attainment |
|  | rs2016933 | 35361970 | Educational attainment |
|  | rs2023878 | 30239722 | Body mass index |
|  | rs2106353 | 34855049 | Educational attainment |
|  | rs215614 | 28892062 | Body mass index |
|  | rs2164300 | 30595370 | Body mass index |
|  | rs2240326 | 35361970 | Educational attainment |
|  | rs2358016 | 30038396 | Educational attainment (years of education) |
|  | rs2734839 | 30038396 | Educational attainment |
|  | rs2744961 | 34594039 | Body mass index |
|  | rs2834005 | 34855049 | Educational attainment |
|  | rs2838771 | 31669095 | Body mass index |
|  | rs329122 | 34594039 | Body mass index |
|  | rs3828917 | 34021172 | Body mass index |
|  | rs3863241 | 35361970 | Educational attainment |
|  | rs4382592 | 34594039 | Body mass index |
|  | rs4713692 | 36581621 | Body mass index |
|  | rs6722661 | 31669095 | Body mass index |
|  | rs6780459 | 25673413 | Body mass index |
|  | rs7032155 | 35361970 | Educational attainment |
|  | rs7206608 | 30595370 | Body mass index |
|  | rs7241572 | 34855049 | Educational attainment |
|  | rs7600261 | 34855049 | Educational attainment |
|  | rs7612999 | 30595370 | Body mass index |
|  | rs7685686 | 35361970 | Educational attainment |
|  | rs773109 | 30038396 | Educational attainment (years of education) |
|  | rs7942368 | 36581621 | Body mass index |
|  | rs903959 | 31669095 | Body mass index |
|  | rs9372625 | 34855049 | Educational attainment |
|  | rs9373363 | 30239722 | Body mass index |
|  | rs9396740 | 35361970 | Educational attainment |
|  | rs942065 | 35361970 | Educational attainment |
|  | rs9517313 | 25201988 | Educational attainment |
|  | rs9529055 | 35361970 | Educational attainment |
|  | rs9542729 | 35361970 | Educational attainment |
|  | rs9615905 | 30595370 | Body mass index |
|  | rs9636202 | 35361970 | Educational attainment |
|  | rs9940128 | 31669095 | Body mass index |
|  | rs12598916 | 30038396 | Educational attainment (MTAG) |
|  | rs13409451 | 34855049 | Educational attainment |
|  | rs17379561 | 29273807 | Body mass index |
|  | rs1937450 | 30038396 | Educational attainment (years of education) |
|  | rs2782641 | 30038396 | Educational attainment (years of education) |
|  | rs2815749 | 26426971 | Body mass index |
|  | rs3766823 | 30239722 | Body mass index |
|  | rs569356 | 30038396 | Educational attainment |
|  | rs6711584 | 30595370 | Body mass index |
|  | rs903678 | 30595370 | Body mass index |
| Hypercholesterolemia | rs12740374 | 37844115 | Pain (pleiotropy) |
| Type 2 Diabetes | rs10146997 | 30239722 | Body mass index |
|  | rs1077394 | 30239722 | Body mass index |
|  | rs10906115 | 28892062 | Body mass index |
|  | rs10965250 | 28892062 | Body mass index |
|  | rs11603334 | 36581621 | Body mass index |
|  | rs11708067 | 36581621 | Body mass index |
|  | rs12602912 | 28892062 | Body mass index |
|  | rs1260326 | 30108127 | Body mass index |
|  | rs13266634 | 28892062 | Body mass index |
|  | rs13389219 | 36581621 | Body mass index |
|  | rs1558902 | 26961502 | Body mass index |
|  | rs17782313 | 37280435 | Body mass index |
|  | rs1801282 | 29273807 | Body mass index |
|  | rs2206277 | 26426971 | Body mass index |
|  | rs2237895 | 28892062 | Body mass index |
|  | rs2296172 | 37280435 | Body mass index |
|  | rs2307111 | 30595370 | Body mass index |
|  | rs2395163 | 37280435 | Body mass index |
|  | rs2796441 | 36581621 | Body mass index |
|  | rs2943641 | 25673413 | Body mass index |
|  | rs4607103 | 25673413 | Body mass index |
|  | rs5015480 | 34594039 | Body mass index |
|  | rs5219 | 29273807 | Body mass index |
|  | rs58542926 | 37280435 | Body mass index |
|  | rs7501939 | 28892062 | Body mass index |
|  | rs7633675 | 29381148 | Body mass index |
|  | rs769449 | 36581621 | Body mass index |
|  | rs7756992 | 29381148 | Body mass index |
|  | rs781831 | 35361970 | Educational attainment |
|  | rs7903146 | 25673413 | Body mass index |
|  | rs8108269 | 36581621 | Body mass index |
|  | rs864745 | 30239722 | Body mass index |
|  | rs9388489 | 35361970 | Educational attainment |
|  | rs972283 | 36581621 | Body mass index |
| Hypothyroidism | rs2234167 | 30239722 | Body mass index |
|  | rs434294 | 35361970 | Educational attainment |
|  | rs1432806 | 30595370 | Educational attainment (years of education) |
|  | rs1065386 | 33414549 | Noncognitive aspects of educational attainment |
|  | rs1079418 | 35361970 | Educational attainment |
|  | rs853305 | 35361970 | Educational attainment |
|  | rs11171710 | 30038396 | Educational attainment (years of education) |
|  | rs3184504 | 29273807 | Body mass index |
|  | rs79490353 | 26426971 | Body mass index |
|  | rs12593201 | 35361970 | Educational attainment |

| **Table S8. Removed SNPs of drug history and related traits (confounding factors)** | | | |
| --- | --- | --- | --- |
| Exposures | SNPs | PMID | traits |
| Antithrombotic Agents | rs28601761 | 34594039 | Body mass index |
|  | rs7412 | 36581612 | Body mass index |
|  | rs8126001 | 34021172 | Body mass index (MTAG) |
|  | rs9266344 | 35361970 | Educational attainment |
|  | rs9937053 | 26961502 | Body mass index |
| Beta Blocking Agents | rs1275923 | 36581621 | Body mass index |
|  | rs115699278 | 34021172 | Body mass index |
|  | rs114697479 | 34021172 | Body mass index |
|  | rs7746061 | 28552196 | Weight |
|  | rs143439747 | 34021172 | Body mass index |
|  | rs1930948 | 34021172 | Body mass index |
|  | rs12537785 | 35361970 | Educational attainment |
|  | rs3735533 | 28892062 | Body mass index |
|  | rs972284 | 36581621 | Body mass index |
|  | rs3918226 | 36581621 | Body mass index |
|  | rs7070847 | 35361970 | Educational attainment |
|  | rs505372 | 35361970 | Educational attainment |
|  | rs12945851 | 36581621 | Body mass index |
|  | rs4277405 | 36581621 | Body mass index |
|  | rs11649807 | 36581621 | Body mass index |
| Antihypertensives | rs1731243 | 36581621 | Body mass index |
|  | rs11130360 | 30595370 | Educational attainment (years of education) |
| Drugs for Peptic Ulcer and Gastro-Oesophageal Reflux Disease | —— | —— | —— |
| Statin Medication | rs629301 | 37844115 | Pain (pleiotropy) |
|  | rs1260326 | 30108127 | Body mass index |
|  | rs7608993 | 35361970 | Educational attainment |
|  | rs9844128 | 29273807 | Body mass index |
|  | rs7707394 | 37280435 | Body mass index |
|  | rs1391373 | 35361970 | Educational attainment |
|  | rs998584 | 30239722 | Body mass index |
|  | rs326 | 33500527 | Metabolically unhealthy in obesity |
|  | rs12112877 | 20112360 | Osteoarthritis |
|  | rs28601761 | 34594039 | Weight |
|  | rs7857118 | 37844115 | Pain (pleiotropy) |
|  | rs11196211 | 36581621 | Body mass index |
|  | rs964184 | 23251661 | Obesity-related traits |
|  | rs76895963 | 30595370 | Body mass index |
|  | rs12149545 | 33500527 | Metabolically unhealthy in obesity |
|  | rs217184 | 35361970 | Educational attainment |
|  | rs72831817 | 23517042 | Body mass index in non-asthmatics |
|  | rs143473297 | 30595370 | Body mass index |
|  | rs7412 | 30038396 | Educational attainment (years of education) |
|  | rs1081105 | 35361970 | Educational attainment |
|  | rs1883711 | 36581621 | Body mass index |
| Drugs Used in Diabetes | rs12463617 | 28552196 | Body mass index |
|  | rs2293476 | 31669095 | Body mass index |
|  | rs1260326 | 33619380 | Body mass index and LDL-C (pairwise) |
|  | rs2972143 | 33619380 | Body mass index and triglycerides (pairwise) |
|  | rs10184004 | 29381148 | Body mass index |
|  | rs11720108 | 33619380 | Body mass index and fasting glucose (pairwise) |
|  | rs17036160 | 37280435 | Body mass index |
|  | rs7630554 | 36581621 | Body mass index |
|  | rs116782923 | 33414549 | Cognitive aspects of educational attainment |
|  | rs253413 | 30038396 | Educational attainment (years of education) |
|  | rs10514301 | 37106081 | BMI (standard GWA) |
|  | rs12654493 | 30239722 | Body mass index |
|  | rs7766070 | 22344221 | Body mass index (SNP x SNP interaction) |
|  | rs3798519 | 26426971 | Body mass index |
|  | rs9264942 | 35361970 | Educational attainment |
|  | rs197482 | 35361970 | Educational attainment |
|  | rs860262 | 30239722 | Body mass index |
|  | rs35589574 | 35361970 | Educational attainment |
|  | rs9410573 | 36581621 | Body mass index |
|  | rs7018475 | 28892062 | Body mass index |
|  | rs10965246 | 28892062 | Body mass index |
|  | rs11257655 | 28892062 | Body mass index |
|  | rs7903146 | 25673413 | Body mass index |
|  | rs34744311 | 34594039 | Body mass index |
|  | rs35723063 | 36581621 | Body mass index |
|  | rs2237897 | 28892062 | Body mass index |
|  | rs757110 | 29273807 | Body mass index |
|  | rs116861488 | 30239722 | Body mass index |
|  | rs76895963 | 30595370 | Body mass index |
|  | rs6567160 | 35538205 | Body mass index |
|  | rs10407429 | 36581621 | Body mass index |
|  | rs1801645 | 35361970 | Educational attainment |
|  | rs72631105 | 34594039 | Weight |
|  | rs2510078 | 30038396 | Educational attainment (years of education) |
|  | rs77464186 | 34594039 | Weight |
|  | rs2583938 | 34594039 | Weight |
|  | rs1898885 | 35361970 | Educational attainment |
| Thyroid Preparations | rs2234167 | 30239722 | Body mass index |
|  | rs17020110 | 36581621 | Body mass index |
|  | rs2969903 | 30038396 | Educational attainment (years of education) |
|  | rs2347101 | 30595370 | Body mass index |
|  | rs7441808 | 34021172 | Body mass index |
|  | rs13358767 | 30038396 | Educational attainment (years of education) |
|  | rs479777 | 34021172 | Body mass index |
|  | rs3184504 | 29273807 | Body mass index |
|  | rs705704 | 30239722 | Body mass index |
|  | rs79490353 | 26426971 | Body mass index |
|  | rs11666808 | 31669095 | Body mass index |

| **Table S9. Removed SNPs of daily habits (no confounding factors)** | | | |
| --- | --- | --- | --- |
| Trait | Removing the following SNPs for incompatible alleles | Removing the following SNPs for being palindromic with intermediate allele frequencies | Removing the following SNPs for horizontal pleiotropy |
| Time Spent Watching Television (TV) | —— | —— | —— |
| Frequency of Friend/Family Visits | —— | rs9962650 | —— |
| Physical Activity | —— | —— | —— |
| Smoking History | —— | rs264974 | rs12924052 |
| Alcohol Intake Frequency | rs9958320 | rs1104608, rs62097995 | —— |

| **Table S10. Removed SNPs of disease history (no confounding factors)** | | | |
| --- | --- | --- | --- |
| Trait | Removing the following SNPs for incompatible alleles | Removing the following SNPs for being palindromic with intermediate allele frequencies | Removing the following SNPs for horizontal pleiotropy |
| Coronary Atherosclerosis | —— | —— | —— |
| Hypertension | —— | rs2274224, rs4371736, rs7310615 | —— |
| Gastroesophageal Reflux Disease | —— | rs2145318, rs957345 | rs3793577 |
| Hypercholesterolemia | —— | rs28601761 | —— |
| Type 2 Diabetes | —— | —— | rs3764002,rs35658696,rs10758593 |
| Hypothyroidism | —— | rs2412976, rs2921053 | rs7990020,rs9277559 |

| **Table S11. Removed SNPs of drug history (no confounding factors)** | | | |
| --- | --- | --- | --- |
| Trait | Removing the following SNPs for incompatible alleles | Removing the following SNPs for being palindromic with intermediate allele frequencies | Removing the following SNPs for horizontal pleiotropy |
| Antithrombotic Agents | —— | —— | rs10406816 |
| Beta Blocking Agents | —— | rs415895, rs73029563, rs7310615 | rs56094641,rs12909307 |
| Antihypertensives | —— | —— | —— |
| Drugs for Peptic Ulcer and Gastro-Oesophageal Reflux Disease | —— | —— | rs1619179,rs11611029 |
| Statin Medication | rs73410781 | —— | rs507666 |
| Drugs Used in Diabetes | —— | rs10406327, rs8037894 | rs13064022,rs1574285,rs2494195,rs3764002,rs56094641 |
| Thyroid Preparations | —— | rs11264798, rs2921053, rs7754251 | rs12419272,rs1790588,rs7990020,rs9469899 |

| **Table S12. Characteristics of the GWAS summary data of outcome** | | | | | | | | | |
| --- | --- | --- | --- | --- | --- | --- | --- | --- | --- |
| Phenotypes | Consortium | Ethnicity | Sample size | | Case definition | Sample overlap with exposures data | PMID | SNPs | URL or PMID |
|  |  |  | cases | controls |  |  |  |  |  |
| Knee osteoarthritis | NA | European | 24,955 | 4 378,169 | ICD10:M17.900 | None | 30664745 | 29,999,696 | 30664745 |

| **Table S13. SNPs used as instrumental variable for daily habits** | | | | | | | | | | |
| --- | --- | --- | --- | --- | --- | --- | --- | --- | --- | --- |
| Exposures | SNP | Chr | Pos | Effect Allele | Other Allele | Eaf | Beta | SE | F | P value |
| Time Spent Watching Television (TV) | rs2230590 | 3 | 49936102 | C | T | 0.517564 | 0.0193 | 0.00187 | 21.409199 | 0 |
|  | rs1561915 | 4 | 152479724 | A | G | 0.471373 | 0.013347 | 0.001872 | 10.218031 | 0 |
|  | rs75499503 | 6 | 26145217 | T | C | 0.219955 | -0.018106 | 0.002294 | 12.947077 | 0 |
|  | rs12553324 | 9 | 23347865 | G | C | 0.416036 | -0.016229 | 0.001897 | 14.729463 | 0 |
|  | rs801738 | 11 | 65924217 | G | C | 0.35841 | -0.013909 | 0.001949 | 10.239451 | 0 |
|  | rs7184800 | 16 | 53509131 | A | G | 0.303036 | -0.014525 | 0.002032 | 10.256008 | 0 |
| Frequency of Friend/Family Visits | rs12326476 | 18 | 63486229 | C | T | 0.277914 | 0.016177 | 0.002588 | 12.087885 | 0 |
|  | rs1933720 | 6 | 98297832 | C | T | 0.366054 | 0.018566 | 0.00242 | 18.412737 | 0 |
|  | rs2008156 | 8 | 65254903 | C | T | 0.192329 | -0.019315 | 0.002941 | 13.338908 | 0 |
|  | rs2151502 | 13 | 60484175 | A | G | 0.749095 | -0.016243 | 0.002675 | 11.414105 | 0 |
|  | rs2737226 | 8 | 116639474 | C | T | 0.607741 | 0.017212 | 0.002375 | 16.257375 | 0 |
|  | rs35393280 | 20 | 47543634 | C | A | 0.246454 | 0.0217 | 0.002684 | 20.130744 | 0 |
|  | rs3772912 | 3 | 81698481 | G | A | 0.362794 | -0.016537 | 0.002407 | 14.552013 | 0 |
| Physical Activity | rs10067451 | 5 | 87942506 | A | G | 0.113071 | -0.333144 | 0.060153 | 2619.965989 | 0 |
|  | rs1268539 | 9 | 128195657 | A | C | 0.418775 | 0.213697 | 0.03796 | 2616.376331 | 0 |
|  | rs34719019 | 10 | 21885577 | T | A | 0.272944 | -0.241562 | 0.042142 | 2728.284975 | 0 |
|  | rs2696625 | 17 | 44326864 | G | A | 0.22959 | 0.310074 | 0.044922 | 4051.80676 | 0 |
| Smoking History | rs10819662 | 9 | 102158306 | T | C | 0.531045 | -0.019898 | 0.003179 | 22.697923 | 0 |
|  | rs10956808 | 8 | 92775372 | G | T | 0.421431 | 0.020449 | 0.003216 | 23.471784 | 0 |
|  | rs11129183 | 3 | 25172544 | G | A | 0.528783 | -0.018643 | 0.00317 | 19.935387 | 0 |
|  | rs11611651 | 12 | 133380790 | A | G | 0.087807 | -0.032675 | 0.0056 | 19.685433 | 0 |
|  | rs118203 | 6 | 111658220 | G | T | 0.81739 | 0.035207 | 0.004101 | 42.597097 | 0 |
|  | rs12186738 | 5 | 103816655 | T | G | 0.143008 | 0.024775 | 0.004519 | 17.316298 | 0 |
|  | rs1246265 | 9 | 86761745 | C | T | 0.695761 | -0.020837 | 0.003448 | 21.155702 | 0 |
|  | rs12714592 | 3 | 84387950 | C | A | 0.273518 | -0.020193 | 0.003551 | 18.650806 | 0 |
|  | rs13245145 | 7 | 1645843 | T | C | 0.391822 | 0.018096 | 0.003239 | 17.963115 | 0 |
|  | rs13393501 | 2 | 146143630 | C | T | 0.517559 | 0.028438 | 0.003167 | 46.494821 | 0 |
|  | rs1449390 | 3 | 85577703 | A | G | 0.628276 | 0.027822 | 0.003275 | 41.621377 | 0 |
|  | rs1503055 | 17 | 50174076 | A | C | 0.382116 | 0.018521 | 0.003261 | 18.643417 | 0 |
|  | rs16951001 | 15 | 67854241 | T | G | 0.417857 | -0.017891 | 0.003216 | 17.922328 | 0 |
|  | rs2155290 | 11 | 112851068 | G | C | 0.382697 | -0.040947 | 0.003258 | 91.23575 | 0 |
|  | rs2412773 | 4 | 57893297 | A | C | 0.301288 | 0.019419 | 0.003451 | 18.273509 | 0 |
|  | rs2675609 | 10 | 63636531 | C | T | 0.627407 | 0.018557 | 0.003285 | 18.530777 | 0 |
|  | rs3099769 | 11 | 132188422 | A | G | 0.410763 | -0.021366 | 0.00324 | 25.435158 | 0 |
|  | rs35891966 | 11 | 20129311 | A | G | 0.072035 | 0.034491 | 0.006111 | 18.304729 | 0 |
|  | rs4671379 | 2 | 60495038 | C | T | 0.584392 | -0.017743 | 0.003216 | 17.60123 | 0 |
|  | rs597808 | 12 | 111973358 | G | A | 0.516619 | 0.021577 | 0.003177 | 26.76566 | 0 |
|  | rs62264764 | 3 | 117639575 | A | G | 0.149498 | 0.028529 | 0.004449 | 23.822061 | 0 |
|  | rs73229090 | 8 | 27442127 | A | C | 0.117597 | 0.027169 | 0.004958 | 17.631863 | 0 |
|  | rs77304846 | 4 | 70495353 | C | T | 0.183977 | 0.023338 | 0.004083 | 18.822734 | 0 |
|  | rs77878475 | 16 | 18058548 | A | T | 0.084706 | 0.033097 | 0.005875 | 19.549541 | 0 |
|  | rs9375371 | 6 | 98751680 | A | G | 0.268942 | -0.023889 | 0.003574 | 25.830522 | 0 |
|  | rs9388686 | 6 | 129425564 | C | A | 0.825959 | -0.024495 | 0.004211 | 19.855113 | 0 |
|  | rs9835772 | 3 | 85766025 | T | A | 0.242763 | -0.020805 | 0.003691 | 18.315598 | 0 |
|  | rs9988851 | 11 | 59189233 | C | G | 0.670122 | -0.018607 | 0.003368 | 17.616995 | 0 |
| Alcohol Intake Frequency | rs10792669 | 11 | 82688356 | G | A | 0.505254 | 0.0174322 | 0.00304065 | 17.48546515 | 9.90011E-09 |
|  | rs11039429 | 11 | 47867059 | T | C | 0.454624 | -0.0235595 | 0.00303737 | 31.68216301 | 8.69961E-15 |
|  | rs11750777 | 5 | 166830787 | A | G | 0.209454 | -0.020493 | 0.00372613 | 16.00670873 | 3.79997E-08 |
|  | rs11940694 | 4 | 39414993 | G | A | 0.604193 | -0.0437138 | 0.00311609 | 105.2708534 | 1E-44 |
|  | rs12312693 | 12 | 57511734 | C | T | 0.451772 | -0.0176811 | 0.00305026 | 17.82301185 | 6.80002E-09 |
|  | rs13102973 | 4 | 135900688 | C | T | 0.61881 | -0.0194072 | 0.00311875 | 20.45109253 | 4.90004E-10 |
|  | rs13178443 | 5 | 145615275 | T | C | 0.276349 | -0.0186516 | 0.00338983 | 16.01380128 | 3.79997E-08 |
|  | rs13390019 | 2 | 97797680 | C | T | 0.134041 | 0.0296116 | 0.00449182 | 23.42943978 | 4.30031E-11 |
|  | rs1515591 | 3 | 174213976 | G | T | 0.383186 | 0.0182303 | 0.00311635 | 18.08150076 | 4.90004E-09 |
|  | rs17690703 | 17 | 43925297 | T | C | 0.262687 | 0.0250342 | 0.00343021 | 27.94332648 | 2.90001E-13 |
|  | rs186347 | 14 | 59072226 | T | G | 0.463343 | 0.0179489 | 0.00305071 | 18.43993646 | 0.000000004 |
|  | rs1893659 | 18 | 21080859 | A | C | 0.459939 | -0.029326 | 0.00305313 | 49.18685527 | 7.59976E-22 |
|  | rs1937522 | 13 | 68080817 | G | A | 0.528054 | 0.0168979 | 0.00303207 | 16.37995424 | 0.000000025 |
|  | rs1991083 | 2 | 23887437 | T | C | 0.679886 | -0.0223925 | 0.00325813 | 25.1220737 | 6.29941E-12 |
|  | rs2043677 | 18 | 38313195 | T | C | 0.145599 | 0.0261133 | 0.0043272 | 19.52684583 | 1.6E-09 |
|  | rs2159935 | 4 | 55521017 | A | G | 0.490369 | -0.0185742 | 0.00302582 | 19.84671932 | 8.30004E-10 |
|  | rs2160935 | 8 | 30840651 | T | C | 0.604293 | -0.018718 | 0.00309117 | 19.2853583 | 1.40001E-09 |
|  | rs2622167 | 7 | 153486704 | A | G | 0.428653 | -0.0191155 | 0.0030674 | 20.60011449 | 4.60002E-10 |
|  | rs262240 | 3 | 68408109 | T | C | 0.468553 | -0.017207 | 0.00303483 | 16.9710142 | 1.40001E-08 |
|  | rs2717063 | 2 | 58110969 | A | C | 0.585731 | -0.0203704 | 0.00308457 | 23.1783213 | 4.00037E-11 |
|  | rs28622224 | 4 | 55088093 | T | C | 0.280364 | -0.0186203 | 0.00336834 | 16.10215468 | 0.000000032 |
|  | rs28787109 | 1 | 51218695 | A | G | 0.40423 | 0.0178107 | 0.00308461 | 17.58531953 | 7.69999E-09 |
|  | rs2924321 | 18 | 53125435 | A | G | 0.539592 | -0.0195131 | 0.00305022 | 21.77498315 | 1.6E-10 |
|  | rs34440851 | 8 | 87214346 | T | C | 0.157151 | -0.0226831 | 0.00415058 | 15.68720934 | 4.60002E-08 |
|  | rs34473884 | 10 | 133761285 | A | G | 0.24819 | -0.0203615 | 0.00350346 | 17.80712741 | 6.19998E-09 |
|  | rs4241258 | 2 | 74226102 | T | C | 0.13763 | 0.0250636 | 0.00440325 | 17.16220834 | 1.29999E-08 |
|  | rs4417025 | 1 | 35363679 | A | G | 0.361153 | -0.0188379 | 0.00316516 | 18.84685702 | 2.69998E-09 |
|  | rs4503294 | 1 | 940096 | T | C | 0.565333 | 0.0181476 | 0.00307049 | 18.62878178 | 3.40001E-09 |
|  | rs461599 | 5 | 144136931 | C | A | 0.462259 | -0.0191888 | 0.00303977 | 21.06921813 | 2.69998E-10 |
|  | rs4726481 | 7 | 141668403 | T | G | 0.400576 | 0.0217614 | 0.00310188 | 26.17617871 | 2.29985E-12 |
|  | rs473098 | 2 | 45139779 | T | C | 0.557689 | -0.0217406 | 0.00304345 | 26.83977705 | 9.09913E-13 |
|  | rs4968391 | 17 | 57780943 | T | G | 0.674892 | -0.0192695 | 0.0032265 | 18.75375992 | 2.30001E-09 |
|  | rs550942 | 11 | 58394154 | T | C | 0.823865 | 0.022401 | 0.00398884 | 16.76156384 | 0.00000002 |
|  | rs6030200 | 20 | 35554361 | A | G | 0.31415 | -0.019529 | 0.00327075 | 18.91526156 | 2.39999E-09 |
|  | rs62305780 | 4 | 100290815 | G | C | 0.102253 | -0.0485216 | 0.00506585 | 49.76256562 | 9.8992E-22 |
|  | rs62466318 | 7 | 73042085 | T | C | 0.202827 | -0.0254919 | 0.0037742 | 24.18737771 | 1.39991E-11 |
|  | rs6727281 | 2 | 65558588 | T | C | 0.184023 | -0.024322 | 0.00391964 | 20.44751366 | 5.49997E-10 |
|  | rs6943160 | 7 | 99872071 | C | T | 0.208646 | 0.020627 | 0.00372774 | 16.17069847 | 3.09999E-08 |
|  | rs71651683 | 22 | 24828853 | T | C | 0.0142 | -0.0704589 | 0.0127906 | 15.99646407 | 3.59998E-08 |
|  | rs72769229 | 2 | 2220795 | T | A | 0.154942 | -0.0231352 | 0.00419154 | 16.13161397 | 3.40001E-08 |
|  | rs728538 | 16 | 51205819 | G | T | 0.168868 | 0.0228752 | 0.00406259 | 16.90541017 | 1.79999E-08 |
|  | rs7298932 | 12 | 23727301 | G | A | 0.147849 | -0.0237214 | 0.00431166 | 16.31893901 | 3.79997E-08 |
|  | rs7330939 | 13 | 49971400 | T | C | 0.720352 | -0.0213301 | 0.0034046 | 21.09777398 | 3.69999E-10 |
|  | rs74679146 | 9 | 16287769 | C | T | 0.074515 | -0.0320735 | 0.00575757 | 16.32984929 | 0.000000025 |
|  | rs80292319 | 15 | 76508632 | C | T | 0.057704 | -0.0393728 | 0.00649583 | 19.40322156 | 1.40001E-09 |
|  | rs838145 | 19 | 49248730 | A | G | 0.542982 | 0.0219548 | 0.00305549 | 27.53571568 | 6.70039E-13 |
|  | rs9349379 | 6 | 12903957 | G | A | 0.405493 | -0.0193455 | 0.00308215 | 20.76793786 | 3.50002E-10 |
|  | rs9814516 | 3 | 85407980 | T | G | 0.237423 | -0.0251117 | 0.00355589 | 26.28286185 | 1.59993E-12 |
|  | rs9829192 | 3 | 38569463 | T | G | 0.435133 | 0.0169324 | 0.00305032 | 16.2211347 | 2.80001E-08 |
|  | rs9912298 | 17 | 29735752 | C | A | 0.239585 | 0.0205894 | 0.00358993 | 17.77786167 | 9.69996E-09 |

| **Table S14. SNPs used as instrumental variable for disease history** | | | | | | | | | | |
| --- | --- | --- | --- | --- | --- | --- | --- | --- | --- | --- |
| Exposures | SNP | Chr | Pos | Effect Allele | Other Allele | Eaf | Beta | SE | F | P value |
| Coronary Atherosclerosis | rs10455872 | 6 | 161010118 | G | A | 0.04575 | 0.3289 | 0.03 | 1097.279483 | 5.098E-28 |
|  | rs113113862 | 19 | 11183577 | A | G | 0.2147 | -0.1006 | 0.0152 | 394.0605804 | 3.5059E-11 |
|  | rs11591147 | 1 | 55505647 | T | G | 0.03609 | -0.2204 | 0.0337 | 390.239806 | 6.33432E-11 |
|  | rs117733303 | 6 | 160922870 | G | A | 0.0113 | 0.5536 | 0.0605 | 793.4767061 | 5.73456E-20 |
|  | rs118092637 | 14 | 100058036 | C | T | 0.0183 | 0.3029 | 0.047 | 380.6073887 | 1.17201E-10 |
|  | rs11852887 | 15 | 79030578 | C | A | 0.6411 | -0.0959 | 0.0129 | 489.0948901 | 1.19702E-13 |
|  | rs12705390 | 7 | 106410777 | A | G | 0.2981 | 0.0802 | 0.0135 | 310.5783983 | 2.94904E-09 |
|  | rs191156695 | 15 | 89447985 | T | C | 0.02854 | -0.2902 | 0.0378 | 539.9099179 | 1.75106E-14 |
|  | rs28451064 | 21 | 35593827 | A | G | 0.1534 | 0.1089 | 0.0173 | 355.5608593 | 2.73502E-10 |
|  | rs34537042 | 6 | 134017855 | A | G | 0.183 | 0.0886 | 0.0161 | 270.754676 | 3.37699E-08 |
|  | rs3918226 | 7 | 150690176 | T | C | 0.0699 | 0.1353 | 0.0243 | 274.5694107 | 2.69898E-08 |
|  | rs4835377 | 4 | 148039045 | G | A | 0.7774 | -0.1057 | 0.015 | 446.7010788 | 1.70805E-12 |
|  | rs56225615 | 15 | 79139370 | T | C | 0.1925 | 0.0963 | 0.0157 | 332.7319491 | 9.22805E-10 |
|  | rs59415853 | 4 | 148378225 | A | G | 0.1275 | 0.1132 | 0.0185 | 329.0206628 | 1.035E-09 |
|  | rs653178 | 12 | 112007756 | T | C | 0.584 | -0.0773 | 0.0126 | 335.0754807 | 8.11708E-10 |
|  | rs72661887 | 1 | 38416310 | T | C | 0.5369 | 0.0777 | 0.0125 | 346.5219723 | 4.54904E-10 |
|  | rs750597 | 13 | 111029256 | A | T | 0.3204 | -0.0809 | 0.0133 | 328.9252858 | 1.21501E-09 |
|  | rs9285863 | 5 | 108071655 | C | T | 0.3137 | -0.0815 | 0.0134 | 330.0672024 | 1.258E-09 |
|  | rs9644861 | 9 | 22090935 | T | C | 0.4183 | 0.2062 | 0.0125 | 2431.41744 | 9.89008E-61 |
| Hypertension | rs10059884 | 5 | 32832474 | A | C | 0.5856 | 0.0651 | 0.0092 | 237.187482 | 1.92398E-12 |
|  | rs10185395 | 2 | 164447403 | T | G | 0.243 | 0.0587 | 0.0107 | 146.0642735 | 3.539E-08 |
|  | rs10217559 | 9 | 112751571 | T | C | 0.6936 | 0.0584 | 0.0099 | 167.0583973 | 3.87302E-09 |
|  | rs10757274 | 9 | 22096055 | G | A | 0.432 | 0.0507 | 0.0092 | 145.3486328 | 3.51803E-08 |
|  | rs10786736 | 10 | 104849116 | C | G | 0.09173 | -0.1089 | 0.0158 | 227.8536242 | 5.48656E-12 |
|  | rs11636952 | 15 | 75114322 | C | T | 0.6071 | -0.055 | 0.0094 | 166.3066359 | 4.20901E-09 |
|  | rs117515309 | 17 | 56404934 | A | C | 0.06182 | -0.1088 | 0.019 | 158.228407 | 1.10499E-08 |
|  | rs12509595 | 4 | 81182554 | C | T | 0.3122 | 0.1157 | 0.0099 | 665.3964842 | 1.3369E-31 |
|  | rs1275984 | 2 | 26911509 | C | A | 0.549 | -0.0953 | 0.0092 | 519.8847239 | 3.37987E-25 |
|  | rs12828438 | 12 | 12883570 | G | A | 0.5354 | -0.0606 | 0.0092 | 210.625886 | 5.00956E-11 |
|  | rs13112725 | 4 | 106911742 | C | G | 0.8285 | 0.0697 | 0.0121 | 159.0878183 | 9.34308E-09 |
|  | rs1317181 | 1 | 230873488 | T | G | 0.2099 | 0.0701 | 0.0112 | 187.868394 | 4.03999E-10 |
|  | rs1358350 | 12 | 90101892 | A | T | 0.7541 | 0.0663 | 0.0106 | 187.9047895 | 4.59103E-10 |
|  | rs1374264 | 2 | 164999883 | C | A | 0.4146 | -0.0603 | 0.0092 | 203.4691655 | 6.94704E-11 |
|  | rs143439093 | 1 | 11981282 | G | A | 0.07436 | -0.1373 | 0.0175 | 299.4096689 | 5.00956E-15 |
|  | rs145153053 | 17 | 45138033 | G | A | 0.1963 | 0.0707 | 0.0115 | 181.7830362 | 7.54397E-10 |
|  | rs149150643 | 4 | 185877562 | C | T | 0.04503 | -0.1328 | 0.0222 | 174.8082 | 2.27002E-09 |
|  | rs167479 | 19 | 11526765 | T | G | 0.4249 | -0.0833 | 0.0093 | 391.5706444 | 2.66011E-19 |
|  | rs16853076 | 3 | 168774754 | C | T | 0.06089 | -0.1096 | 0.0192 | 158.3052547 | 1.064E-08 |
|  | rs188155432 | 5 | 60972519 | T | C | 0.02363 | 0.1977 | 0.0303 | 207.9170991 | 7.04369E-11 |
|  | rs1888693 | 10 | 18440444 | A | G | 0.386 | 0.06 | 0.0094 | 196.7046631 | 1.45399E-10 |
|  | rs189498068 | 19 | 12018867 | G | A | 0.01165 | -0.2554 | 0.0436 | 173.1195401 | 4.689E-09 |
|  | rs2032451 | 6 | 26092170 | T | G | 0.1098 | 0.0886 | 0.0146 | 176.8638584 | 1.28499E-09 |
|  | rs2392929 | 7 | 106414069 | G | T | 0.2979 | 0.062 | 0.01 | 185.33887 | 5.20703E-10 |
|  | rs2627313 | 15 | 81006712 | T | C | 0.4006 | 0.054 | 0.0093 | 161.375856 | 7.35106E-09 |
|  | rs2643826 | 3 | 27562988 | T | C | 0.4047 | 0.0627 | 0.0093 | 218.3948326 | 1.75106E-11 |
|  | rs2782981 | 10 | 115781547 | C | T | 0.6546 | 0.0799 | 0.0096 | 333.1671667 | 8.71365E-17 |
|  | rs34071855 | 1 | 10798489 | G | C | 0.415 | 0.1033 | 0.0093 | 599.3429446 | 1.04304E-28 |
|  | rs35427 | 12 | 115556307 | G | T | 0.3644 | -0.0592 | 0.0096 | 187.1225153 | 5.91398E-10 |
|  | rs35619711 | 17 | 59482169 | G | C | 0.7217 | 0.0614 | 0.0102 | 174.5338204 | 1.65299E-09 |
|  | rs3790604 | 1 | 113046879 | A | C | 0.1709 | 0.1321 | 0.0121 | 571.9029951 | 1.40314E-27 |
|  | rs3796585 | 4 | 156639174 | A | G | 0.3465 | -0.0751 | 0.0096 | 294.6823753 | 4.48126E-15 |
|  | rs3918226 | 7 | 150690176 | T | C | 0.0699 | 0.1721 | 0.0179 | 444.8966212 | 7.08435E-22 |
|  | rs4685218 | 3 | 14894140 | T | C | 0.09873 | 0.0984 | 0.0153 | 198.6360198 | 1.29799E-10 |
|  | rs4932373 | 15 | 91429287 | C | A | 0.2674 | 0.0837 | 0.0103 | 316.7288857 | 5.58085E-16 |
|  | rs604723 | 11 | 100610546 | C | T | 0.741 | 0.0632 | 0.0104 | 176.6986484 | 1.358E-09 |
|  | rs62426324 | 6 | 127142458 | T | C | 0.5009 | 0.0528 | 0.0091 | 160.630123 | 7.13198E-09 |
|  | rs62434119 | 6 | 150984431 | T | C | 0.07595 | -0.1345 | 0.0172 | 292.9454364 | 6.23017E-15 |
|  | rs629929 | 13 | 22318331 | T | G | 0.5795 | 0.0595 | 0.0093 | 198.8923563 | 1.36201E-10 |
|  | rs6668768 | 1 | 25136203 | T | C | 0.2144 | 0.0668 | 0.0111 | 173.2396291 | 1.865E-09 |
|  | rs6860901 | 5 | 127871750 | T | C | 0.2979 | 0.0682 | 0.01 | 224.335908 | 7.67892E-12 |
|  | rs6918791 | 6 | 126218961 | G | C | 0.728 | 0.0574 | 0.0103 | 150.3508446 | 2.15402E-08 |
|  | rs6961048 | 7 | 27328187 | G | C | 0.1355 | 0.0735 | 0.0133 | 145.8289441 | 3.38797E-08 |
|  | rs7123467 | 11 | 10155174 | G | C | 0.202 | 0.0682 | 0.0114 | 172.8182036 | 2.474E-09 |
|  | rs7134677 | 12 | 54441498 | T | C | 0.3819 | -0.0748 | 0.0094 | 304.771978 | 2.13304E-15 |
|  | rs71554083 | 7 | 27245993 | G | GAGCA | 0.9044 | 0.1376 | 0.0157 | 378.0021775 | 1.92309E-18 |
|  | rs72779268 | 9 | 136454663 | A | C | 0.06436 | -0.1222 | 0.0187 | 207.3307596 | 6.31539E-11 |
|  | rs7483477 | 11 | 1920255 | G | T | 0.2348 | 0.0759 | 0.0109 | 238.7103854 | 3.12176E-12 |
|  | rs7545442 | 1 | 27260783 | T | C | 0.07246 | 0.098 | 0.0176 | 148.75066 | 2.73602E-08 |
|  | rs76778513 | 1 | 11833976 | T | A | 0.113 | -0.1253 | 0.0145 | 363.3188715 | 4.6153E-18 |
|  | rs78412528 | 20 | 57734912 | A | G | 0.1674 | 0.1406 | 0.0122 | 637.6422138 | 1.0311E-30 |
|  | rs79873333 | 17 | 47357173 | T | C | 0.04758 | -0.1364 | 0.0215 | 194.3700597 | 2.10902E-10 |
|  | rs9899012 | 17 | 61545486 | A | G | 0.08387 | -0.1068 | 0.0166 | 202.061193 | 1.409E-10 |
| Gastroesophageal Reflux Disease | rs10010963 | 4 | 159839313 | T | C | 0.616433 | -0.02698 | 0.004947 | 39.626429 | 0 |
|  | rs10133111 | 14 | 103377321 | A | G | 0.162996 | 0.041787 | 0.006508 | 54.855349 | 0 |
|  | rs1021363 | 10 | 106610839 | G | A | 0.641992 | -0.031217 | 0.005022 | 51.572005 | 0 |
|  | rs10837002 | 11 | 38565727 | G | C | 0.35122 | 0.027649 | 0.005037 | 40.105578 | 0 |
|  | rs11953061 | 5 | 120144025 | T | C | 0.338908 | 0.02816 | 0.005087 | 40.904872 | 0 |
|  | rs1431196 | 18 | 50832102 | G | A | 0.428432 | 0.03242 | 0.004864 | 59.266074 | 0 |
|  | rs1479405 | 12 | 15387519 | T | C | 0.3217 | 0.031484 | 0.005151 | 49.803932 | 0 |
|  | rs1592757 | 5 | 103889998 | C | G | 0.355772 | 0.031105 | 0.005025 | 51.060137 | 0 |
|  | rs1716171 | 12 | 123716376 | T | C | 0.790024 | 0.038398 | 0.005904 | 56.319224 | 0 |
|  | rs2043539 | 7 | 12253880 | A | G | 0.41866 | 0.027206 | 0.004865 | 41.474955 | 0 |
|  | rs2396133 | 7 | 109197067 | G | A | 0.475329 | 0.029355 | 0.004818 | 49.48096 | 0 |
|  | rs2396766 | 7 | 114318071 | A | G | 0.47308 | 0.032206 | 0.004819 | 59.536616 | 0 |
|  | rs324769 | 12 | 83969240 | T | C | 0.449179 | -0.02677 | 0.004833 | 40.821806 | 0 |
|  | rs3793577 | 9 | 23737627 | G | A | 0.538279 | 0.027031 | 0.00485 | 41.810041 | 0 |
|  | rs3863241 | 8 | 73890335 | T | C | 0.52696 | 0.032498 | 0.004815 | 60.623026 | 0 |
|  | rs7527682 | 1 | 189172684 | G | A | 0.53725 | -0.026684 | 0.004822 | 40.75616 | 0 |
|  | rs7541875 | 1 | 190957589 | G | A | 0.426069 | 0.027397 | 0.00485 | 42.259679 | 0 |
|  | rs761777 | 10 | 134938075 | G | A | 0.254034 | 0.034534 | 0.005545 | 52.03772 | 0 |
|  | rs7675588 | 4 | 80734978 | A | C | 0.794635 | -0.033523 | 0.005954 | 42.222969 | 0 |
| Hypercholesterolemia | rs1367117 | 2 | 21263900 | A | G | 0.338871 | 0.0155585 | 0.000840047 | 12.48296985 | 1.52651E-76 |
|  | rs6511720 | 19 | 11202306 | T | G | 0.118816 | -0.0261322 | 0.00122887 | 16.45774531 | 2.77332E-100 |
|  | rs7412 | 19 | 45412079 | T | C | 0.0809878 | -0.0374088 | 0.00145797 | 23.97693494 | 4.7315E-145 |
|  | rs964184 | 11 | 116648917 | C | G | 0.867927 | -0.021563 | 0.00117398 | 12.26808165 | 2.60435E-75 |
| Type 2 Diabetes | rs10830963 | 11 | 92708710 | G | C | 0.2849 | 0.0849 | 0.007 | 338.9742922 | 1.34586E-32 |
|  | rs10842994 | 12 | 27965150 | T | C | 0.1784 | -0.0629 | 0.0079 | 133.6211841 | 9.43192E-16 |
|  | rs12571751 | 10 | 80942631 | G | A | 0.4614 | -0.0541 | 0.0061 | 167.6430076 | 3.39313E-21 |
|  | rs1359790 | 13 | 80717156 | A | G | 0.2678 | -0.0593 | 0.0071 | 158.9145871 | 2.89601E-17 |
|  | rs1531343 | 12 | 66174894 | C | G | 0.1263 | 0.0657 | 0.0095 | 109.7299294 | 1.30707E-13 |
|  | rs1801212 | 4 | 6302519 | A | G | 0.7481 | 0.0683 | 0.0074 | 202.6787492 | 1.09597E-24 |
|  | rs2191349 | 7 | 15064309 | T | G | 0.5498 | 0.0488 | 0.006 | 135.8241258 | 1.166E-18 |
|  | rs243021 | 2 | 60584819 | A | G | 0.479 | 0.0457 | 0.0063 | 120.0807346 | 4.64622E-14 |
|  | rs2925979 | 16 | 81534790 | C | T | 0.6949 | -0.0375 | 0.0069 | 68.65953383 | 2.488E-08 |
|  | rs328 | 8 | 19819724 | G | C | 0.0966 | -0.0524 | 0.0102 | 55.17504797 | 6.82402E-09 |
|  | rs340874 | 1 | 214159256 | C | T | 0.5032 | 0.0454 | 0.0061 | 118.7125041 | 1.41286E-14 |
|  | rs35720761 | 2 | 43519977 | T | C | 0.105 | -0.0733 | 0.0101 | 116.3252043 | 4.58142E-15 |
|  | rs41278853 | 22 | 30416527 | G | A | 0.0738 | -0.0826 | 0.0121 | 107.4338775 | 5.61048E-13 |
|  | rs4457053 | 5 | 76424949 | A | G | 0.7298 | -0.0402 | 0.0073 | 73.38933369 | 8.80846E-11 |
|  | rs4502156 | 15 | 62383155 | C | T | 0.4759 | -0.036 | 0.0063 | 74.44413347 | 1.20099E-09 |
|  | rs459193 | 5 | 55806751 | G | A | 0.6992 | 0.0529 | 0.0069 | 135.6178536 | 2.82488E-15 |
|  | rs4812831 | 20 | 43018260 | A | G | 0.1307 | 0.0617 | 0.0096 | 99.63368686 | 8.48399E-10 |
|  | rs505922 | 9 | 136149229 | C | T | 0.371 | 0.0376 | 0.0065 | 75.98041338 | 9.07006E-10 |
|  | rs516946 | 8 | 41519248 | C | T | 0.7785 | 0.0655 | 0.0074 | 170.5189931 | 1.86595E-20 |
|  | rs55834942 | 12 | 121437114 | A | G | 0.1702 | -0.0578 | 0.0083 | 108.6959691 | 5.45255E-13 |
|  | rs60980157 | 9 | 139235415 | T | C | 0.2286 | -0.062 | 0.008 | 156.2223523 | 3.19301E-16 |
|  | rs6813195 | 4 | 153520475 | T | C | 0.3102 | -0.0485 | 0.0069 | 115.9579734 | 1.10103E-13 |
|  | rs7177055 | 15 | 77832762 | A | G | 0.6575 | 0.0539 | 0.007 | 150.7708038 | 7.5405E-15 |
|  | rs7202877 | 16 | 75247245 | G | T | 0.1111 | -0.064 | 0.0097 | 93.17366086 | 5.62989E-12 |
|  | rs730497 | 7 | 44223721 | A | G | 0.1624 | 0.0476 | 0.0081 | 70.97724472 | 4.01098E-10 |
|  | rs731839 | 19 | 33899065 | A | G | 0.6351 | -0.0379 | 0.0066 | 76.66521994 | 5.153E-10 |
|  | rs738409 | 22 | 44324727 | G | C | 0.2388 | 0.0396 | 0.0071 | 65.64261753 | 2.121E-10 |
|  | rs7572857 | 2 | 65296798 | A | G | 0.1541 | -0.0524 | 0.009 | 82.43468568 | 8.25201E-09 |
|  | rs8042680 | 15 | 91521337 | A | C | 0.4335 | 0.0439 | 0.0067 | 109.0295175 | 2.32488E-11 |
|  | rs9379084 | 6 | 7231843 | A | G | 0.1157 | -0.0795 | 0.0121 | 149.0198179 | 1.14393E-13 |
| Hypothyroidism | rs10075764 | 5 | 35841449 | G | A | 0.302413 | -0.057 | 0.0104 | 157.9643356 | 4.25598E-08 |
|  | rs10126000 | 9 | 21578617 | A | C | 0.688692 | -0.0683 | 0.0104 | 230.6434111 | 5.13334E-11 |
|  | rs10424978 | 19 | 4837557 | A | C | 0.624328 | -0.0775 | 0.0102 | 325.1360494 | 2.76694E-14 |
|  | rs10917477 | 1 | 19861106 | G | A | 0.386901 | 0.064 | 0.01 | 224.0525592 | 1.75299E-10 |
|  | rs11406335 | 13 | 99934479 | TG | T | 0.417169 | -0.057 | 0.0103 | 182.0982735 | 3.43598E-08 |
|  | rs11420448 | 2 | 55854285 | GT | G | 0.0467006 | 0.1533 | 0.0275 | 241.3017978 | 2.39199E-08 |
|  | rs114285740 | 1 | 107947273 | C | G | 0.0273776 | 0.1669 | 0.0301 | 170.9668123 | 3.05999E-08 |
|  | rs11675342 | 2 | 1407628 | T | C | 0.387729 | 0.0906 | 0.01 | 450.234722 | 1.39605E-19 |
|  | rs11875260 | 18 | 77179239 | G | A | 0.172925 | 0.0751 | 0.0135 | 185.9509982 | 2.53998E-08 |
|  | rs12117927 | 1 | 236629134 | A | C | 0.476541 | 0.0627 | 0.0105 | 226.1441741 | 2.28802E-09 |
|  | rs12379417 | 9 | 127029736 | A | G | 0.317559 | 0.0583 | 0.0103 | 169.7780988 | 1.51001E-08 |
|  | rs12582330 | 12 | 103892941 | T | G | 0.624365 | -0.061 | 0.0109 | 201.2045061 | 2.05201E-08 |
|  | rs12984428 | 19 | 50199616 | A | G | 0.35604 | -0.0659 | 0.0102 | 229.6196141 | 1.10601E-10 |
|  | rs13090803 | 3 | 105934953 | T | G | 0.190624 | 0.0829 | 0.0128 | 244.5533832 | 9.00119E-11 |
|  | rs13109179 | 4 | 10727528 | A | G | 0.461895 | 0.0647 | 0.01 | 239.9596739 | 9.4189E-11 |
|  | rs1364450 | 6 | 109389508 | C | A | 0.126235 | 0.0886 | 0.0139 | 199.6224328 | 1.97102E-10 |
|  | rs141232332 | 6 | 31075601 | G | GTTT | 0.451125 | 0.2243 | 0.0127 | 2940.364148 | 4.47507E-70 |
|  | rs142997491 | 16 | 50729820 | G | A | 0.0135637 | 0.2385 | 0.0412 | 175.4285609 | 7.01504E-09 |
|  | rs1479565 | 5 | 76544157 | A | G | 0.498866 | 0.0975 | 0.0101 | 549.580008 | 7.52835E-22 |
|  | rs1534430 | 2 | 12644736 | T | C | 0.428394 | -0.086 | 0.0101 | 418.3384202 | 1.44012E-17 |
|  | rs187707293 | 4 | 179647723 | A | T | 0.0133864 | 0.2419 | 0.044 | 178.1431693 | 3.98602E-08 |
|  | rs2111485 | 2 | 163110536 | G | A | 0.479023 | 0.0813 | 0.0102 | 380.895533 | 1.42692E-15 |
|  | rs2114702 | 14 | 81405135 | A | T | 0.26671 | 0.07 | 0.0111 | 220.9831099 | 2.99902E-10 |
|  | rs2247314 | 6 | 167370230 | C | T | 0.376495 | -0.086 | 0.0104 | 400.978849 | 1.06488E-16 |
|  | rs229528 | 22 | 37581677 | T | C | 0.496016 | 0.0903 | 0.01 | 471.0606568 | 2.31206E-19 |
|  | rs2445608 | 8 | 128181302 | A | G | 0.430366 | -0.0593 | 0.0101 | 198.7501334 | 3.78704E-09 |
|  | rs244685 | 5 | 133423890 | G | T | 0.79915 | -0.0858 | 0.0132 | 272.5944555 | 7.0583E-11 |
|  | rs28418426 | 6 | 32619654 | C | T | 0.501169 | 0.1877 | 0.0133 | 2063.476148 | 2.20902E-45 |
|  | rs2988277 | 1 | 167431352 | T | C | 0.287022 | 0.0593 | 0.0106 | 165.8597308 | 2.48702E-08 |
|  | rs307558 | 3 | 12095130 | A | G | 0.742625 | -0.0688 | 0.0119 | 208.599899 | 8.01493E-09 |
|  | rs3087243 | 2 | 204738919 | A | G | 0.386088 | -0.1466 | 0.0102 | 1184.465512 | 4.77309E-47 |
|  | rs3118469 | 10 | 6101129 | T | A | 0.295174 | 0.0803 | 0.0106 | 309.5798174 | 3.8212E-14 |
|  | rs34536443 | 19 | 10463118 | C | G | 0.0440279 | -0.1863 | 0.0263 | 337.1975168 | 1.46083E-12 |
|  | rs3775291 | 4 | 187004074 | T | C | 0.287788 | -0.0649 | 0.0108 | 199.0382902 | 1.646E-09 |
|  | rs4409785 | 11 | 95311422 | C | T | 0.142566 | 0.1069 | 0.0133 | 322.4050813 | 8.04452E-16 |
|  | rs4529854 | 10 | 64043975 | T | C | 0.723438 | -0.0768 | 0.0107 | 272.243328 | 6.55843E-13 |
|  | rs4835534 | 4 | 149636388 | C | T | 0.156356 | -0.1421 | 0.0132 | 616.30601 | 7.05667E-27 |
|  | rs5912815 | X | 78466147 | G | T | 0.576761 | -0.0511 | 0.0084 | 146.8899644 | 1.05499E-09 |
|  | rs61759532 | 17 | 7240391 | T | C | 0.188941 | 0.0905 | 0.0122 | 289.5890254 | 1.42102E-13 |
|  | rs61877856 | 11 | 617378 | T | C | 0.197495 | -0.0658 | 0.0115 | 158.1491399 | 1.137E-08 |
|  | rs6679677 | 1 | 114303808 | A | C | 0.108444 | 0.3637 | 0.0159 | 3020.706566 | 2.3878E-115 |
|  | rs6908626 | 6 | 91005743 | T | G | 0.17075 | 0.1441 | 0.0141 | 680.6913863 | 2.03704E-24 |
|  | rs7030280 | 9 | 100535267 | T | C | 0.745664 | 0.2075 | 0.0108 | 1910.52646 | 1.02E-82 |
|  | rs71508903 | 10 | 63779871 | T | C | 0.210816 | 0.0934 | 0.0125 | 335.0061779 | 9.33684E-14 |
|  | rs7223956 | 17 | 45373844 | C | T | 0.897005 | -0.0902 | 0.0144 | 173.2575676 | 4.26904E-10 |
|  | rs73192661 | 3 | 188128794 | T | C | 0.428858 | -0.1061 | 0.01 | 638.1235131 | 4.05229E-26 |
|  | rs736374 | 11 | 35266944 | A | G | 0.375103 | 0.0832 | 0.0103 | 374.6554274 | 6.00344E-16 |
|  | rs7441808 | 4 | 26090375 | G | A | 0.21091 | 0.0766 | 0.0111 | 225.1878881 | 5.16654E-12 |
|  | rs7488011 | 12 | 9925336 | T | C | 0.356485 | 0.1052 | 0.0111 | 587.2959069 | 2.51826E-21 |
|  | rs7574865 | 2 | 191964633 | G | T | 0.74252 | -0.1321 | 0.0117 | 773.0001483 | 1.66686E-29 |
|  | rs7742626 | 6 | 136132496 | C | T | 0.267343 | 0.0686 | 0.0116 | 212.5366713 | 3.41201E-09 |
|  | rs78765971 | 1 | 108362169 | G | GAC | 0.146942 | 0.2444 | 0.0162 | 1749.420693 | 1.67919E-51 |
|  | rs881858 | 6 | 43806609 | A | G | 0.747899 | 0.0665 | 0.0108 | 192.2208073 | 8.45707E-10 |
|  | rs911760 | 9 | 5438435 | A | C | 0.212143 | 0.0879 | 0.0125 | 297.9831703 | 1.95389E-12 |
|  | rs926103 | 1 | 156784982 | C | T | 0.697985 | -0.0678 | 0.0104 | 223.4556 | 7.65244E-11 |
|  | rs9264277 | 6 | 31224667 | C | T | 0.645071 | -0.0862 | 0.0111 | 392.8786957 | 9.03233E-15 |
|  | rs9271365 | 6 | 32586794 | G | T | 0.443466 | 0.2484 | 0.0105 | 3614.954465 | 4.9091E-123 |
|  | rs9273371 | 6 | 32626565 | T | C | 0.193385 | 0.0791 | 0.014 | 225.0636486 | 1.64502E-08 |
|  | rs9497965 | 6 | 148521292 | T | C | 0.409035 | 0.0827 | 0.0102 | 381.7564296 | 3.70681E-16 |
|  | rs9511151 | 13 | 24786576 | A | G | 0.279567 | -0.0976 | 0.0106 | 443.2651137 | 3.07822E-20 |
|  | rs9902341 | 17 | 40278522 | T | C | 0.172257 | 0.0801 | 0.0129 | 210.9344501 | 4.68198E-10 |

| **Table S15. SNPs used as instrumental variable for drug history** | | | | | | | | | | |
| --- | --- | --- | --- | --- | --- | --- | --- | --- | --- | --- |
| Exposures | SNP | Chr | Pos | Effect Allele | Other Allele | Eaf | Beta | SE | F | P value |
| Antithrombotic Agents | rs10757274 | 9 | 22096055 | G | A | 0.48365 | 0.0666 | 0.0051 | 255.5064054 | 1.02896E-38 |
|  | rs12212242 | 6 | 31768971 | G | A | 0.0717414 | 0.1002 | 0.0153 | 154.0884771 | 5.56288E-11 |
|  | rs12293813 | 11 | 110224141 | A | G | 0.276068 | 0.038 | 0.0068 | 66.45788685 | 2.56201E-08 |
|  | rs12509595 | 4 | 81182554 | C | T | 0.296618 | 0.0324 | 0.0056 | 50.42940034 | 8.37703E-09 |
|  | rs12639654 | 4 | 111705295 | T | C | 0.292343 | 0.0591 | 0.0064 | 166.545655 | 3.67621E-20 |
|  | rs142158911 | 19 | 11190534 | A | G | 0.113105 | -0.0919 | 0.0107 | 195.3153057 | 8.04452E-18 |
|  | rs1894401 | 15 | 91429042 | A | G | 0.685426 | -0.0318 | 0.0058 | 50.20440464 | 3.44802E-08 |
|  | rs1894692 | 1 | 169467654 | A | G | 0.978978 | -0.1501 | 0.0235 | 106.8134706 | 1.761E-10 |
|  | rs2107595 | 7 | 19049388 | A | G | 0.257217 | 0.0414 | 0.0062 | 75.41566824 | 2.84315E-11 |
|  | rs2865827 | 16 | 75304366 | G | A | 0.541273 | 0.0485 | 0.0081 | 134.5787524 | 2.57099E-09 |
|  | rs3134988 | 6 | 32640772 | A | G | 0.777872 | -0.0663 | 0.0097 | 175.0706993 | 6.58719E-12 |
|  | rs4299376 | 2 | 44072576 | T | G | 0.850372 | -0.0417 | 0.0072 | 50.94492281 | 6.94704E-09 |
|  | rs4629495 | 4 | 185305366 | T | C | 0.895671 | 0.0547 | 0.0089 | 64.3851755 | 9.61302E-10 |
|  | rs545971 | 9 | 136143372 | T | C | 0.388525 | 0.038 | 0.0053 | 79.00920613 | 1.23994E-12 |
|  | rs56214516 | 17 | 43836953 | C | A | 0.0893014 | 0.0487 | 0.0086 | 44.40922347 | 1.60498E-08 |
|  | rs57828240 | 16 | 73041940 | G | T | 0.269073 | 0.0362 | 0.0062 | 59.34715947 | 4.42395E-09 |
|  | rs629301 | 1 | 109818306 | T | G | 0.858018 | 0.0683 | 0.0071 | 130.9422325 | 1.10994E-21 |
|  | rs662799 | 11 | 116663707 | A | G | 0.782436 | -0.0515 | 0.0072 | 104.005764 | 8.8247E-13 |
|  | rs6823438 | 4 | 148392728 | T | C | 0.232888 | 0.0378 | 0.0064 | 58.77951749 | 3.34003E-09 |
|  | rs6838973 | 4 | 111765495 | T | C | 0.485451 | -0.0311 | 0.0052 | 55.63109005 | 1.75598E-09 |
|  | rs74617384 | 6 | 160997118 | T | A | 0.0815418 | 0.1149 | 0.0123 | 228.0101366 | 1.0539E-20 |
|  | rs75802599 | 8 | 19843579 | C | G | 0.0896813 | -0.075 | 0.0118 | 105.7868519 | 2.01298E-10 |
|  | rs7989823 | 13 | 110959643 | C | A | 0.625868 | 0.0627 | 0.0085 | 212.25491 | 1.46285E-13 |
| Beta Blocking Agents | rs10434005 | 4 | 111367841 | G | A | 0.643501 | -0.0423 | 0.0075 | 94.549603 | 0 |
|  | rs10757272 | 9 | 22088260 | T | C | 0.560243 | 0.0714 | 0.0067 | 289.7962 | 0 |
|  | rs10776752 | 1 | 113044328 | T | G | 0.172002 | 0.0789 | 0.0097 | 204.410076 | 0 |
|  | rs10857147 | 4 | 81181072 | T | A | 0.290888 | 0.0844 | 0.0073 | 339.170778 | 0 |
|  | rs114815710 | 6 | 30942121 | A | G | 0.039811 | 0.1068 | 0.018 | 100.437756 | 0 |
|  | rs13409330 | 2 | 43381472 | G | A | 0.644467 | -0.0548 | 0.0086 | 158.582239 | 0 |
|  | rs167479 | 19 | 11526765 | T | G | 0.48612 | -0.051 | 0.0066 | 149.735591 | 0 |
|  | rs180940 | 10 | 115722411 | G | A | 0.577577 | 0.0383 | 0.0069 | 82.429131 | 0 |
|  | rs1926032 | 10 | 104829469 | T | C | 0.150696 | -0.091 | 0.0103 | 244.446594 | 0 |
|  | rs2493135 | 1 | 230851428 | G | C | 0.586438 | 0.0406 | 0.0072 | 92.082464 | 0 |
|  | rs258317 | 16 | 89732238 | T | C | 0.246063 | -0.055 | 0.0082 | 129.303184 | 0 |
|  | rs2625268 | 4 | 156478647 | T | C | 0.632832 | 0.0401 | 0.007 | 86.05605 | 0 |
|  | rs2681485 | 12 | 90025622 | A | G | 0.602354 | 0.0407 | 0.0067 | 91.389595 | 0 |
|  | rs34687996 | 3 | 48175411 | A | C | 0.659369 | 0.0498 | 0.0087 | 128.34242 | 0 |
|  | rs35441 | 12 | 115553115 | T | C | 0.326684 | -0.0548 | 0.007 | 152.228791 | 0 |
|  | rs36060036 | 16 | 20361950 | T | C | 0.167201 | -0.0658 | 0.011 | 138.921536 | 0 |
|  | rs41305070 | 1 | 150666860 | G | A | 0.193895 | 0.047 | 0.0083 | 79.518594 | 0 |
|  | rs4684242 | 3 | 14895105 | C | G | 0.252821 | 0.0437 | 0.0079 | 83.086197 | 0 |
|  | rs487987 | 8 | 102902997 | C | A | 0.290053 | -0.0424 | 0.0074 | 85.265124 | 0 |
|  | rs4923536 | 11 | 28422496 | G | A | 0.570695 | 0.0409 | 0.0068 | 94.403373 | 0 |
|  | rs4970834 | 1 | 109814880 | T | C | 0.127497 | -0.0655 | 0.0097 | 109.945886 | 0 |
|  | rs569550 | 11 | 1887068 | G | T | 0.550885 | 0.059 | 0.0071 | 198.55736 | 0 |
|  | rs59980837 | 1 | 115827266 | T | G | 0.017514 | 0.1959 | 0.0313 | 152.182398 | 0 |
|  | rs6039211 | 20 | 8616588 | G | A | 0.43501 | -0.0482 | 0.0068 | 131.566426 | 0 |
|  | rs62043959 | 16 | 81525204 | C | A | 0.195673 | -0.0482 | 0.0084 | 84.215124 | 0 |
|  | rs62434125 | 6 | 151002371 | C | T | 0.051779 | -0.0819 | 0.0145 | 75.846423 | 0 |
|  | rs629042 | 13 | 22318506 | C | G | 0.688994 | 0.0409 | 0.0072 | 82.557596 | 0 |
|  | rs648103 | 3 | 27550301 | C | T | 0.736839 | -0.0506 | 0.0076 | 114.377701 | 0 |
|  | rs664485 | 11 | 107184296 | G | A | 0.808939 | 0.0506 | 0.0084 | 91.148067 | 0 |
|  | rs6838973 | 4 | 111765495 | T | C | 0.476277 | -0.038 | 0.0066 | 82.957598 | 0 |
|  | rs6892983 | 5 | 127845030 | A | C | 0.408844 | 0.0545 | 0.0084 | 165.459413 | 0 |
|  | rs6932812 | 6 | 166180947 | G | C | 0.079035 | -0.068 | 0.0125 | 77.51551 | 0 |
|  | rs6988985 | 8 | 144007104 | C | T | 0.420048 | -0.0411 | 0.0068 | 94.7866 | 0 |
|  | rs7183988 | 15 | 91428589 | G | T | 0.659503 | -0.0738 | 0.0073 | 282.176711 | 0 |
|  | rs72821788 | 10 | 63444428 | A | G | 0.098467 | -0.0755 | 0.0109 | 116.579638 | 0 |
|  | rs7412 | 19 | 45412079 | T | C | 0.06286 | -0.0735 | 0.0134 | 73.289794 | 0 |
|  | rs7442660 | 5 | 157803630 | A | G | 0.258058 | -0.0512 | 0.0076 | 115.632025 | 0 |
|  | rs74617384 | 6 | 160997118 | T | A | 0.080049 | 0.1102 | 0.0152 | 206.194236 | 0 |
|  | rs75245746 | 20 | 57730418 | C | T | 0.106182 | 0.0937 | 0.0134 | 192.095988 | 0 |
|  | rs7543029 | 1 | 193305723 | C | T | 0.409878 | 0.0388 | 0.0069 | 83.866982 | 0 |
|  | rs75725917 | 4 | 111710921 | A | C | 0.259361 | 0.0775 | 0.0085 | 266.153822 | 0 |
|  | rs7733331 | 5 | 32828846 | C | T | 0.591847 | 0.0411 | 0.0067 | 93.990815 | 0 |
|  | rs80157433 | 4 | 148370548 | G | A | 0.215502 | 0.0537 | 0.0084 | 112.313027 | 0 |
|  | rs830450 | 8 | 76928831 | C | T | 0.561797 | 0.0464 | 0.0068 | 122.114178 | 0 |
|  | rs880315 | 1 | 10796866 | C | T | 0.48985 | 0.0627 | 0.0071 | 226.550484 | 0 |
|  | rs9285476 | 6 | 134159976 | G | C | 0.372646 | -0.0396 | 0.007 | 84.437002 | 0 |
|  | rs9286351 | 4 | 138441530 | G | A | 0.411756 | 0.0445 | 0.0067 | 110.496629 | 0 |
|  | rs9895661 | 17 | 59456589 | T | C | 0.66573 | 0.0451 | 0.0078 | 104.269358 | 0 |
| Antihypertensives | rs17035646 | 1 | 10796547 | A | G | 0.52048 | 0.1001 | 0.014 | 578.457288 | 8.2054E-13 |
|  | rs12485763 | 3 | 27583317 | A | G | 0.0252372 | 0.2321 | 0.0424 | 305.8142768 | 4.40301E-08 |
|  | rs9375461 | 6 | 127159310 | A | C | 0.437442 | 0.0784 | 0.0132 | 349.1808907 | 2.99102E-09 |
|  | rs4980379 | 11 | 1888614 | T | C | 0.503026 | 0.0816 | 0.0135 | 384.3858742 | 1.61599E-09 |
|  | rs11105352 | 12 | 90026462 | A | G | 0.278886 | -0.0858 | 0.0153 | 341.7494416 | 1.98802E-08 |
| Drugs for Peptic Ulcer and Gastro-Oesophageal Reflux Disease | rs1384103 | 8 | 73852753 | G | A | 0.815609 | -0.0404 | 0.0071 | 56.52134274 | 1.373E-08 |
|  | rs2891168 | 9 | 22098619 | G | A | 0.481025 | 0.0315 | 0.0053 | 57.03811362 | 3.35498E-09 |
|  | rs4358685 | 7 | 21589083 | G | T | 0.552773 | -0.0447 | 0.0077 | 113.7978075 | 6.55602E-09 |
| Statin Medication | rs10045497 | 5 | 74636484 | A | C | 0.4394 | 0.1008 | 0.009 | 578.9330825 | 4.97279E-29 |
|  | rs10798336 | 1 | 175111946 | A | G | 0.2021 | 0.0623 | 0.0113 | 144.2280055 | 3.08603E-08 |
|  | rs11134475 | 5 | 156399950 | G | A | 0.669 | 0.0783 | 0.0095 | 313.3092412 | 2.25216E-16 |
|  | rs112108223 | 12 | 4271088 | A | G | 0.02237 | -0.1847 | 0.0306 | 171.9640055 | 1.58899E-09 |
|  | rs113414093 | 2 | 219859171 | A | G | 0.08536 | 0.106 | 0.0162 | 202.2524109 | 5.64157E-11 |
|  | rs114165349 | 1 | 27021913 | C | G | 0.03745 | 0.2449 | 0.0236 | 499.7460751 | 3.96643E-25 |
|  | rs115415310 | 4 | 74312759 | C | T | 0.03822 | 0.3496 | 0.0235 | 1043.383561 | 3.90661E-50 |
|  | rs115864880 | 2 | 20947443 | A | G | 0.01914 | -0.224 | 0.0328 | 217.2094515 | 7.97811E-12 |
|  | rs11591147 | 1 | 55505647 | T | G | 0.03609 | -0.6142 | 0.0245 | 3101.764039 | 6.6222E-139 |
|  | rs1169288 | 12 | 121416650 | C | A | 0.3741 | 0.0721 | 0.0093 | 280.8254613 | 8.54083E-15 |
|  | rs117733303 | 6 | 160922870 | G | A | 0.0113 | 0.4908 | 0.0427 | 622.7456924 | 1.56099E-30 |
|  | rs11783515 | 8 | 59320389 | G | A | 0.6215 | -0.0566 | 0.0092 | 173.7039496 | 8.87708E-10 |
|  | rs12047069 | 1 | 2205548 | C | A | 0.5899 | -0.058 | 0.0091 | 187.6058385 | 2.026E-10 |
|  | rs12154632 | 7 | 44939191 | T | C | 0.3642 | -0.0544 | 0.0093 | 157.931456 | 5.60106E-09 |
|  | rs13255048 | 8 | 18276640 | A | G | 0.1802 | 0.0778 | 0.0116 | 206.1645605 | 2.13698E-11 |
|  | rs1367117 | 2 | 21263900 | A | G | 0.281 | 0.1782 | 0.0099 | 1495.80202 | 3.52371E-72 |
|  | rs17248720 | 19 | 11198187 | T | C | 0.102 | -0.3993 | 0.0149 | 3462.292801 | 2.2856E-157 |
|  | rs1727736 | 19 | 45039709 | C | T | 0.852 | 0.0917 | 0.0128 | 244.5550872 | 6.77954E-13 |
|  | rs1766 | 6 | 32627795 | G | A | 0.4509 | 0.055 | 0.0097 | 172.6326094 | 1.64399E-08 |
|  | rs182695896 | 4 | 74813227 | C | A | 0.02574 | 0.3852 | 0.0283 | 862.809271 | 3.4033E-42 |
|  | rs187270432 | 19 | 45514061 | A | G | 0.0336 | 0.221 | 0.0252 | 366.1636254 | 1.58599E-18 |
|  | rs2497318 | 10 | 94432000 | T | C | 0.4498 | 0.0548 | 0.009 | 171.3017959 | 1.13799E-09 |
|  | rs2642442 | 1 | 220973563 | T | C | 0.7058 | 0.0557 | 0.0099 | 148.4599773 | 1.64199E-08 |
|  | rs2727270 | 11 | 61603237 | T | C | 0.2681 | -0.1088 | 0.0101 | 537.0853904 | 5.06757E-27 |
|  | rs2740486 | 9 | 107666513 | G | T | 0.4262 | -0.0519 | 0.009 | 151.8084698 | 9.27598E-09 |
|  | rs3005923 | 1 | 56801542 | A | G | 0.02593 | -0.4409 | 0.0288 | 1141.230579 | 8.85727E-53 |
|  | rs34931250 | 17 | 66879927 | T | C | 0.06623 | 0.1052 | 0.0181 | 157.7378062 | 6.32295E-09 |
|  | rs3808348 | 7 | 1028448 | T | C | 0.2791 | -0.0563 | 0.01 | 146.9672956 | 1.71902E-08 |
|  | rs41279633 | 7 | 44580876 | T | G | 0.2099 | 0.0993 | 0.011 | 377.59868 | 1.7398E-19 |
|  | rs4240624 | 8 | 9184231 | A | G | 0.8572 | 0.1205 | 0.0135 | 410.5308466 | 3.93822E-19 |
|  | rs4809404 | 20 | 62753138 | G | A | 0.5725 | -0.0533 | 0.0091 | 160.2452345 | 4.101E-09 |
|  | rs4927235 | 1 | 55907494 | G | A | 0.5912 | 0.0555 | 0.0091 | 171.5904561 | 1.00399E-09 |
|  | rs494198 | 1 | 55518528 | A | C | 0.5707 | 0.1273 | 0.009 | 921.0912634 | 5.57571E-45 |
|  | rs59888912 | 1 | 219795761 | G | A | 0.2629 | -0.0561 | 0.0102 | 140.5359809 | 3.81997E-08 |
|  | rs62130040 | 19 | 10774809 | G | C | 0.08902 | 0.0947 | 0.0158 | 167.6267416 | 2.24999E-09 |
|  | rs6459450 | 6 | 16124560 | C | T | 0.3784 | -0.0541 | 0.0092 | 158.6608539 | 4.84596E-09 |
|  | rs646356 | 1 | 55167773 | A | T | 0.8317 | 0.0882 | 0.0121 | 251.1594821 | 2.60016E-13 |
|  | rs6883310 | 5 | 74298032 | A | C | 0.1164 | 0.0912 | 0.0142 | 197.2226521 | 1.56899E-10 |
|  | rs73561834 | 9 | 136758967 | G | C | 0.05665 | 0.1142 | 0.0193 | 160.6295783 | 3.59898E-09 |
|  | rs74617384 | 6 | 160997118 | T | A | 0.04577 | 0.258 | 0.0215 | 673.0086815 | 3.33426E-33 |
|  | rs76128868 | 4 | 71390321 | G | C | 0.02498 | 0.1691 | 0.0287 | 160.5141346 | 3.59302E-09 |
|  | rs76224003 | 2 | 44020982 | G | A | 0.1591 | 0.0772 | 0.0122 | 183.8048676 | 2.65601E-10 |
|  | rs76643124 | 9 | 136356448 | A | G | 0.1039 | -0.0905 | 0.0149 | 175.7708229 | 1.16501E-09 |
|  | rs76866386 | 2 | 44075483 | C | T | 0.08372 | -0.2227 | 0.0162 | 882.3274672 | 5.02343E-43 |
|  | rs77645768 | 4 | 73668828 | A | G | 0.02676 | 0.4487 | 0.028 | 1219.583148 | 6.52229E-58 |
|  | rs79915079 | 19 | 11229066 | A | G | 0.02969 | -0.2609 | 0.0265 | 453.0960058 | 7.5544E-23 |
|  | rs8821 | 17 | 18148534 | T | C | 0.03439 | -0.1353 | 0.0247 | 140.0788637 | 4.41499E-08 |
|  | rs9849171 | 3 | 12622623 | C | G | 0.3889 | -0.0528 | 0.0092 | 152.6893301 | 1.00501E-08 |
| Drugs Used in Diabetes | rs1002389 | 7 | 156968323 | G | C | 0.419056 | -0.0574 | 0.0076 | 184.9022215 | 5.91289E-14 |
|  | rs10134560 | 14 | 91930152 | T | C | 0.48685 | 0.0411 | 0.0075 | 97.20858309 | 4.82603E-08 |
|  | rs10771369 | 12 | 27960152 | C | G | 0.238895 | -0.0639 | 0.0086 | 171.1249945 | 1.33999E-13 |
|  | rs10803625 | 2 | 234249735 | C | T | 0.470759 | -0.0469 | 0.0078 | 126.2666965 | 2.082E-09 |
|  | rs10872467 | 6 | 137298243 | A | G | 0.609884 | -0.07 | 0.0102 | 268.9463469 | 8.63376E-12 |
|  | rs112023979 | 10 | 64923227 | G | A | 0.0891637 | -0.0686 | 0.0117 | 88.02853778 | 5.07598E-09 |
|  | rs112674299 | 3 | 63982670 | T | C | 0.206172 | -0.0717 | 0.0089 | 193.9726926 | 8.50159E-16 |
|  | rs115521560 | 6 | 32019382 | C | A | 0.026107 | 0.219 | 0.0278 | 281.3401226 | 3.1369E-15 |
|  | rs11820002 | 11 | 128389259 | C | G | 0.199959 | 0.0567 | 0.0097 | 118.4898144 | 5.665E-09 |
|  | rs11853073 | 15 | 91530158 | C | T | 0.326645 | 0.0599 | 0.008 | 181.9175142 | 6.97108E-14 |
|  | rs11947917 | 4 | 71751764 | C | G | 0.89645 | -0.0822 | 0.0111 | 144.5372213 | 1.53993E-13 |
|  | rs12052410 | 2 | 111859287 | A | C | 0.169929 | -0.0538 | 0.0098 | 94.04090742 | 4.33301E-08 |
|  | rs12910361 | 15 | 77782335 | G | A | 0.60301 | 0.0742 | 0.0079 | 304.1395841 | 4.41164E-21 |
|  | rs13059456 | 3 | 122010284 | C | T | 0.568757 | -0.0552 | 0.0099 | 172.2627405 | 2.76299E-08 |
|  | rs13094957 | 3 | 23457080 | C | T | 0.186849 | -0.1118 | 0.0099 | 438.7458362 | 1.06194E-29 |
|  | rs13266634 | 8 | 118184783 | T | C | 0.344257 | -0.1044 | 0.0078 | 569.0816778 | 1.09799E-40 |
|  | rs1355619 | 9 | 98270171 | G | A | 0.292279 | -0.0474 | 0.0085 | 107.0619116 | 2.186E-08 |
|  | rs1359790 | 13 | 80717156 | A | G | 0.280945 | -0.08 | 0.0084 | 298.3338922 | 1.48286E-21 |
|  | rs1377807 | 17 | 4045440 | C | G | 0.29362 | 0.0532 | 0.0084 | 135.2608831 | 2.844E-10 |
|  | rs1575972 | 9 | 22301092 | A | T | 0.0424023 | -0.1171 | 0.0177 | 128.2876759 | 3.59087E-11 |
|  | rs17168486 | 7 | 14898282 | T | C | 0.255837 | 0.0562 | 0.0084 | 138.5611322 | 2.44174E-11 |
|  | rs17458 | 19 | 55693015 | T | C | 0.124815 | 0.0627 | 0.0103 | 98.92126193 | 1.38998E-09 |
|  | rs1800961 | 20 | 43042364 | T | C | 0.0231856 | 0.2064 | 0.0277 | 222.4863544 | 9.20238E-14 |
|  | rs2090670 | 2 | 196948592 | A | G | 0.249726 | 0.0494 | 0.0085 | 105.3294067 | 6.63697E-09 |
|  | rs2280613 | 7 | 69755741 | A | G | 0.26625 | 0.0521 | 0.0084 | 122.1768092 | 7.12705E-10 |
|  | rs243024 | 2 | 60583665 | A | G | 0.536174 | 0.0502 | 0.0079 | 144.4201276 | 1.62701E-10 |
|  | rs2648731 | 5 | 52072194 | A | G | 0.254072 | 0.0504 | 0.0085 | 110.9041737 | 3.16796E-09 |
|  | rs2905798 | 17 | 29536990 | G | A | 0.364798 | -0.0437 | 0.008 | 101.9357367 | 4.08301E-08 |
|  | rs3130931 | 6 | 31134888 | C | T | 0.659404 | 0.0735 | 0.0079 | 279.921132 | 1.01111E-20 |
|  | rs34072724 | 7 | 130432469 | A | G | 0.422627 | -0.0466 | 0.0079 | 122.0845754 | 3.63898E-09 |
|  | rs340874 | 1 | 214159256 | C | T | 0.50136 | 0.0488 | 0.0076 | 137.1856294 | 1.66702E-10 |
|  | rs34165267 | 13 | 91942919 | T | C | 0.230009 | -0.0511 | 0.0092 | 106.5341645 | 3.111E-08 |
|  | rs34584161 | 13 | 26776999 | G | A | 0.314814 | -0.0669 | 0.0081 | 222.6224038 | 1.35394E-16 |
|  | rs348330 | 1 | 229672955 | A | G | 0.504937 | -0.0569 | 0.0083 | 186.5694322 | 8.4879E-12 |
|  | rs35399713 | 14 | 69437141 | A | G | 0.46679 | 0.0604 | 0.0101 | 209.3620055 | 2.536E-09 |
|  | rs35452727 | 7 | 44266184 | T | C | 0.107685 | 0.0642 | 0.0115 | 91.22258003 | 2.05802E-08 |
|  | rs3887925 | 3 | 186665645 | T | C | 0.504398 | 0.0514 | 0.0076 | 152.202374 | 1.07103E-11 |
|  | rs3935875 | 9 | 139238824 | G | A | 0.799255 | 0.0865 | 0.01 | 276.9625584 | 5.01765E-18 |
|  | rs410504 | 4 | 85301055 | G | A | 0.953369 | 0.1479 | 0.0239 | 224.2499462 | 5.68604E-10 |
|  | rs4273712 | 6 | 126964510 | G | A | 0.340677 | 0.0524 | 0.0079 | 142.1197365 | 2.68473E-11 |
|  | rs464605 | 5 | 55807370 | T | C | 0.644247 | 0.0717 | 0.008 | 271.8182847 | 4.44734E-19 |
|  | rs4689394 | 4 | 6291003 | G | C | 0.742745 | 0.1054 | 0.0114 | 490.6222035 | 2.55211E-20 |
|  | rs4719433 | 7 | 15065003 | C | T | 0.603421 | 0.06 | 0.0079 | 198.6169542 | 3.13329E-14 |
|  | rs4736819 | 8 | 41509915 | T | C | 0.532015 | 0.0673 | 0.0075 | 260.1245182 | 3.13329E-19 |
|  | rs4793090 | 17 | 40686342 | A | G | 0.611233 | -0.0442 | 0.0077 | 106.9446092 | 8.56505E-09 |
|  | rs57017296 | 4 | 1351509 | C | T | 0.600451 | -0.0588 | 0.01 | 191.2221053 | 4.67401E-09 |
|  | rs5762925 | 22 | 29369398 | C | A | 0.412709 | 0.0421 | 0.0077 | 98.95768659 | 4.06097E-08 |
|  | rs58432198 | 1 | 51256091 | T | C | 0.106977 | -0.0806 | 0.0124 | 143.0135842 | 9.06776E-11 |
|  | rs6679677 | 1 | 114303808 | A | C | 0.102872 | 0.1073 | 0.0191 | 245.0697438 | 1.826E-08 |
|  | rs68088379 | 12 | 121448059 | C | T | 0.165379 | 0.063 | 0.0097 | 126.2239094 | 9.98849E-11 |
|  | rs697239 | 10 | 80947438 | C | T | 0.454275 | -0.0802 | 0.0075 | 368.1657707 | 2.08689E-26 |
|  | rs72951506 | 6 | 118011723 | T | C | 0.190681 | -0.0577 | 0.0093 | 118.3695595 | 6.47098E-10 |
|  | rs730831 | 4 | 1240299 | G | T | 0.138783 | -0.1016 | 0.0101 | 284.6583992 | 8.10401E-24 |
|  | rs7313668 | 12 | 71449521 | T | G | 0.402362 | 0.0427 | 0.0077 | 100.9965775 | 2.59801E-08 |
|  | rs73541271 | 16 | 20337186 | T | C | 0.0418389 | 0.121 | 0.0167 | 135.2428739 | 4.82836E-13 |
|  | rs7409148 | 19 | 13094298 | T | C | 0.814171 | 0.0829 | 0.0151 | 239.8043232 | 3.75198E-08 |
|  | rs7501939 | 17 | 36101156 | C | T | 0.6297 | -0.1002 | 0.0078 | 541.347137 | 2.40492E-37 |
|  | rs7528167 | 1 | 154285487 | G | A | 0.349217 | 0.047 | 0.008 | 115.6587036 | 4.17802E-09 |
|  | rs75418188 | 17 | 6945483 | T | C | 0.0781321 | 0.1468 | 0.0182 | 358.3565802 | 8.59409E-16 |
|  | rs7686964 | 4 | 153483294 | G | A | 0.388115 | -0.0605 | 0.0078 | 200.4063179 | 1.06709E-14 |
|  | rs77059113 | 2 | 43672508 | G | T | 0.0734371 | -0.1753 | 0.0224 | 483.2691134 | 4.4978E-15 |
|  | rs7947981 | 11 | 2626334 | G | A | 0.103798 | -0.1216 | 0.0165 | 317.4484361 | 1.48388E-13 |
|  | rs8017808 | 14 | 38848419 | T | G | 0.400147 | -0.0469 | 0.0083 | 121.6420334 | 1.60702E-08 |
|  | rs8614 | 17 | 27588806 | A | C | 0.304929 | -0.0496 | 0.0082 | 120.1321209 | 1.501E-09 |
|  | rs889512 | 16 | 75242012 | G | C | 0.0740814 | -0.1104 | 0.0163 | 192.735476 | 1.31886E-11 |
|  | rs924150 | 19 | 31829903 | C | A | 0.413293 | -0.0533 | 0.0082 | 158.7622493 | 9.48418E-11 |
|  | rs9273368 | 6 | 32626475 | A | G | 0.341965 | 0.1157 | 0.0078 | 697.4875855 | 2.27405E-49 |
|  | rs9358341 | 6 | 20525488 | C | A | 0.299038 | 0.0564 | 0.0082 | 153.6644321 | 6.82496E-12 |
|  | rs9379084 | 6 | 7231843 | A | G | 0.1664 | -0.0744 | 0.01 | 176.986068 | 9.75888E-14 |
|  | rs9411206 | 9 | 139405501 | G | A | 0.633729 | 0.0544 | 0.0086 | 158.3126893 | 2.59298E-10 |
|  | rs9515039 | 13 | 109940939 | C | T | 0.516273 | 0.0427 | 0.0077 | 104.8928603 | 2.52302E-08 |
|  | rs9873519 | 3 | 124921457 | T | C | 0.456285 | 0.0475 | 0.0078 | 128.9721515 | 1.009E-09 |
|  | rs9996713 | 4 | 167409871 | A | G | 0.381286 | 0.0465 | 0.0082 | 117.5180959 | 1.53102E-08 |
| Thyroid Preparations | rs10021637 | 4 | 149657642 | G | A | 0.211865 | -0.1302 | 0.0111 | 655.182345 | 0 |
|  | rs10036386 | 5 | 76543603 | T | C | 0.522063 | 0.058 | 0.009 | 193.505798 | 0 |
|  | rs10098103 | 8 | 23397081 | C | T | 0.395401 | -0.0595 | 0.0099 | 195.114528 | 0 |
|  | rs10204169 | 2 | 55890603 | T | G | 0.053698 | 0.1113 | 0.0202 | 145.057454 | 0 |
|  | rs10279209 | 7 | 37424240 | A | G | 0.235447 | 0.0756 | 0.0119 | 237.275914 | 0 |
|  | rs1032129 | 8 | 119951900 | C | A | 0.449731 | -0.0537 | 0.009 | 164.479393 | 0 |
|  | rs10489626 | 1 | 67793171 | G | C | 0.111535 | 0.0746 | 0.0122 | 127.064191 | 0 |
|  | rs10751647 | 11 | 306884 | C | T | 0.54545 | -0.0517 | 0.009 | 152.724406 | 0 |
|  | rs10759262 | 9 | 110663574 | A | C | 0.527562 | 0.0529 | 0.0087 | 160.749884 | 0 |
|  | rs10761620 | 10 | 64057202 | G | A | 0.676724 | -0.0752 | 0.009 | 285.43773 | 0 |
|  | rs10815220 | 9 | 5425847 | G | A | 0.194981 | 0.0609 | 0.01 | 134.13858 | 0 |
|  | rs11154825 | 6 | 136145479 | T | C | 0.23382 | 0.0566 | 0.0104 | 132.238789 | 0 |
|  | rs113473633 | 4 | 103449131 | G | A | 0.022128 | -0.1786 | 0.0312 | 159.072125 | 0 |
|  | rs11485999 | 1 | 38372798 | A | C | 0.38711 | -0.0604 | 0.0088 | 199.552886 | 0 |
|  | rs115266049 | 6 | 30431011 | A | G | 0.024012 | -0.2212 | 0.0348 | 264.518906 | 0 |
|  | rs11675342 | 2 | 1407628 | T | C | 0.377184 | 0.0938 | 0.0088 | 477.674939 | 0 |
|  | rs11714050 | 3 | 39357107 | G | A | 0.140714 | 0.0848 | 0.0127 | 200.464462 | 0 |
|  | rs11714843 | 3 | 119221064 | A | T | 0.182346 | 0.0849 | 0.0119 | 247.873674 | 0 |
|  | rs11783023 | 8 | 141639262 | T | C | 0.625986 | -0.0675 | 0.0094 | 246.03808 | 0 |
|  | rs12422517 | 12 | 103889031 | G | A | 0.658168 | -0.0663 | 0.0092 | 228.061058 | 0 |
|  | rs12423545 | 12 | 9939224 | T | C | 0.396483 | 0.103 | 0.0093 | 587.237618 | 0 |
|  | rs12482947 | 21 | 43852037 | C | T | 0.588784 | 0.0543 | 0.0089 | 164.535997 | 0 |
|  | rs1257926 | 14 | 98692996 | A | G | 0.550387 | 0.0493 | 0.0087 | 138.592059 | 0 |
|  | rs12634152 | 3 | 188121019 | T | C | 0.509173 | -0.1333 | 0.0087 | 1031.200846 | 0 |
|  | rs12697352 | 5 | 35837234 | A | G | 0.284982 | -0.0634 | 0.0092 | 188.816385 | 0 |
|  | rs12748718 | 1 | 200682088 | T | C | 0.126999 | 0.0837 | 0.0131 | 179.042399 | 0 |
|  | rs12980063 | 19 | 50196992 | G | A | 0.342628 | -0.0548 | 0.0089 | 155.882665 | 0 |
|  | rs13136820 | 4 | 40307564 | T | C | 0.693912 | -0.0709 | 0.0093 | 246.255703 | 0 |
|  | rs13360007 | 5 | 156577720 | G | A | 0.093795 | 0.078 | 0.0131 | 119.140637 | 0 |
|  | rs13380830 | 17 | 40297658 | G | C | 0.16929 | 0.0911 | 0.0115 | 269.244715 | 0 |
|  | rs13398375 | 2 | 8451701 | C | T | 0.277209 | -0.0611 | 0.0096 | 172.411976 | 0 |
|  | rs144309607 | 19 | 10492274 | T | C | 0.043598 | -0.1466 | 0.0225 | 206.619219 | 0 |
|  | rs1479924 | 4 | 123387600 | A | G | 0.76602 | 0.057 | 0.0097 | 134.180501 | 0 |
|  | rs1516973 | 8 | 129545257 | T | C | 0.071668 | -0.0911 | 0.0144 | 127.222164 | 0 |
|  | rs1570214 | 11 | 35289819 | G | A | 0.385522 | 0.077 | 0.0088 | 324.170433 | 0 |
|  | rs174599 | 11 | 61621556 | C | G | 0.388631 | -0.0633 | 0.0089 | 219.528411 | 0 |
|  | rs2002311 | 18 | 77173979 | G | A | 0.31646 | 0.0565 | 0.0093 | 159.145588 | 0 |
|  | rs2111485 | 2 | 163110536 | G | A | 0.45037 | 0.0664 | 0.009 | 251.732792 | 0 |
|  | rs2274780 | 9 | 127075021 | C | T | 0.513276 | 0.0513 | 0.0086 | 151.514655 | 0 |
|  | rs2445610 | 8 | 128197088 | G | A | 0.36528 | -0.073 | 0.0089 | 285.064442 | 0 |
|  | rs244690 | 5 | 133421744 | A | G | 0.799071 | -0.1052 | 0.0124 | 410.41229 | 0 |
|  | rs2673826 | 10 | 89807131 | G | A | 0.437427 | 0.0511 | 0.0086 | 148.081062 | 0 |
|  | rs2823272 | 21 | 16798586 | A | T | 0.305663 | -0.0572 | 0.0093 | 160.038161 | 0 |
|  | rs2844532 | 6 | 31351175 | C | G | 0.24614 | -0.1254 | 0.0107 | 675.49881 | 0 |
|  | rs2856698 | 6 | 32636376 | G | C | 0.249467 | 0.2523 | 0.0094 | 2810.021909 | 0 |
|  | rs2858483 | 22 | 37586672 | C | A | 0.516126 | 0.0938 | 0.0087 | 507.95031 | 0 |
|  | rs3008034 | 6 | 166043862 | C | T | 0.257235 | -0.0597 | 0.0095 | 156.941137 | 0 |
|  | rs3087243 | 2 | 204738919 | A | G | 0.383822 | -0.1734 | 0.0087 | 1660.236363 | 0 |
|  | rs3093668 | 6 | 31546495 | C | G | 0.031648 | -0.1967 | 0.0222 | 273.549212 | 0 |
|  | rs34591253 | 7 | 128721877 | T | C | 0.085283 | 0.0765 | 0.0133 | 105.168435 | 0 |
|  | rs34822076 | 3 | 105927531 | C | T | 0.233322 | 0.0902 | 0.0108 | 335.94061 | 0 |
|  | rs34965214 | 6 | 32609545 | T | C | 0.271329 | 0.1507 | 0.0108 | 1042.765394 | 0 |
|  | rs35776863 | 17 | 7226957 | A | G | 0.149843 | 0.088 | 0.0109 | 227.495814 | 0 |
|  | rs35782497 | 4 | 187001230 | A | G | 0.319389 | -0.0809 | 0.0092 | 328.373593 | 0 |
|  | rs366327 | 4 | 87980030 | T | A | 0.184729 | 0.0676 | 0.0119 | 158.614693 | 0 |
|  | rs3784099 | 14 | 68749927 | A | G | 0.219236 | -0.0654 | 0.0098 | 168.747864 | 0 |
|  | rs3792411 | 3 | 141626123 | G | A | 0.162054 | -0.0661 | 0.0119 | 136.712777 | 0 |
|  | rs4271723 | 2 | 160326049 | T | C | 0.239787 | 0.0543 | 0.0099 | 123.834723 | 0 |
|  | rs4320727 | 1 | 25351581 | A | G | 0.615376 | 0.0493 | 0.0089 | 132.551756 | 0 |
|  | rs434294 | 5 | 102599626 | G | A | 0.322449 | -0.0641 | 0.0097 | 206.973264 | 0 |
|  | rs4366932 | 2 | 191592247 | T | C | 0.437684 | 0.0514 | 0.0087 | 149.846724 | 0 |
|  | rs4409785 | 11 | 95311422 | C | T | 0.136418 | 0.1453 | 0.0117 | 575.290187 | 0 |
|  | rs4678561 | 3 | 37253945 | A | G | 0.358751 | -0.0487 | 0.0089 | 125.709084 | 0 |
|  | rs4888945 | 16 | 79316533 | G | A | 0.627275 | -0.0665 | 0.0101 | 238.453408 | 0 |
|  | rs573741 | 1 | 65455477 | A | C | 0.898982 | 0.086 | 0.0152 | 154.790821 | 0 |
|  | rs5865 | 2 | 98373006 | T | C | 0.531679 | -0.0544 | 0.0091 | 169.842433 | 0 |
|  | rs6111715 | 20 | 17860022 | C | G | 0.14709 | -0.0786 | 0.0115 | 178.656954 | 0 |
|  | rs61778692 | 1 | 38650752 | G | A | 0.158141 | -0.0654 | 0.0106 | 131.204573 | 0 |
|  | rs61907718 | 11 | 128172470 | A | G | 0.20836 | 0.067 | 0.0105 | 170.667197 | 0 |
|  | rs631408 | 8 | 61541273 | C | T | 0.457102 | 0.0498 | 0.0088 | 141.820725 | 0 |
|  | rs6505765 | 18 | 12782849 | G | C | 0.343491 | 0.0764 | 0.009 | 303.740229 | 0 |
|  | rs6584283 | 10 | 101290301 | C | T | 0.553983 | -0.0501 | 0.0086 | 142.914758 | 0 |
|  | rs6679677 | 1 | 114303808 | A | C | 0.102923 | 0.4108 | 0.0149 | 3701.407195 | 0 |
|  | rs6914622 | 6 | 148514301 | T | G | 0.362087 | 0.0859 | 0.0092 | 393.602983 | 0 |
|  | rs7088058 | 10 | 124149352 | T | C | 0.336207 | -0.0559 | 0.0089 | 160.725187 | 0 |
|  | rs7090530 | 10 | 6110875 | A | C | 0.628766 | 0.0842 | 0.0088 | 382.133847 | 0 |
|  | rs71508903 | 10 | 63779871 | T | C | 0.214221 | 0.139 | 0.0109 | 753.426624 | 0 |
|  | rs7195228 | 16 | 50353529 | G | C | 0.187192 | -0.0696 | 0.0117 | 169.882697 | 0 |
|  | rs722749 | 12 | 68500638 | G | C | 0.296169 | -0.0497 | 0.009 | 118.626847 | 0 |
|  | rs7254729 | 19 | 4832362 | C | T | 0.628618 | -0.0802 | 0.0088 | 346.638978 | 0 |
|  | rs7327883 | 13 | 24786385 | T | C | 0.209601 | 0.1197 | 0.0099 | 548.920533 | 0 |
|  | rs73316435 | 17 | 45329477 | T | C | 0.179924 | 0.0906 | 0.0119 | 279.426519 | 0 |
|  | rs744253 | 10 | 6393009 | G | A | 0.714902 | 0.0738 | 0.0097 | 256.055101 | 0 |
|  | rs75051580 | 3 | 5012125 | C | T | 0.216862 | 0.059 | 0.0107 | 136.224222 | 0 |
|  | rs75191394 | 14 | 81014720 | A | T | 0.119862 | -0.0666 | 0.0122 | 107.795873 | 0 |
|  | rs7565618 | 2 | 12640347 | T | C | 0.538094 | -0.079 | 0.0088 | 358.121284 | 0 |
|  | rs7582694 | 2 | 191970120 | G | C | 0.732867 | -0.1381 | 0.0101 | 865.784971 | 0 |
|  | rs7583027 | 2 | 62544391 | C | A | 0.732191 | 0.057 | 0.0093 | 146.81409 | 0 |
|  | rs7596240 | 2 | 242444173 | G | A | 0.186675 | 0.0626 | 0.0101 | 137.097899 | 0 |
|  | rs767593 | 22 | 41779971 | T | G | 0.158455 | -0.0613 | 0.0108 | 115.439501 | 0 |
|  | rs7850258 | 9 | 100549013 | G | A | 0.766002 | 0.1966 | 0.0095 | 1616.901297 | 0 |
|  | rs80190634 | 16 | 67660902 | A | G | 0.028286 | 0.148 | 0.0221 | 138.73081 | 0 |
|  | rs8043085 | 15 | 38828140 | T | G | 0.32815 | 0.0849 | 0.01 | 366.908155 | 0 |
|  | rs853305 | 8 | 133931183 | C | T | 0.714852 | -0.0754 | 0.0093 | 267.332431 | 0 |
|  | rs926103 | 1 | 156784982 | C | T | 0.712988 | -0.0529 | 0.0093 | 131.948987 | 0 |
|  | rs9277392 | 6 | 33050829 | C | G | 0.305343 | -0.1585 | 0.0099 | 1239.609553 | 0 |
|  | rs9291444 | 4 | 10713674 | T | C | 0.498946 | 0.0731 | 0.0086 | 308.282934 | 0 |
|  | rs9298817 | 9 | 21576591 | C | A | 0.722075 | -0.0768 | 0.0093 | 273.071554 | 0 |
|  | rs9303234 | 17 | 8864690 | G | A | 0.747503 | 0.0671 | 0.0113 | 195.915008 | 0 |
|  | rs933243 | 6 | 167403873 | A | C | 0.381337 | -0.1132 | 0.009 | 700.010442 | 0 |
|  | rs9391997 | 6 | 409119 | G | A | 0.455705 | 0.0672 | 0.0087 | 258.371993 | 0 |
|  | rs9457245 | 6 | 167340715 | T | C | 0.056703 | 0.0946 | 0.0171 | 110.272018 | 0 |
|  | rs9625921 | 22 | 30508275 | G | C | 0.288398 | -0.0637 | 0.0092 | 191.975997 | 0 |

| **Table S16. Causal effects of daily habits on knee osteoarthritis risk using two sample mendelian randomization analysis** | | | | | | | | |
| --- | --- | --- | --- | --- | --- | --- | --- | --- |
| Trait | method | nsnp | β | SE | pval | or | or_lci95 | or_uci95 |
| Time Spent Watching Television (TV) | MR Egger | 6 | 5.044161512 | 2.601370241 | 0.124514603 | 155.1141832 | 0.946935629 | 25408.70687 |
|  | Weighted median | 6 | 1.194323279 | 0.371122446 | 0.001290257 | 3.301322939 | 1.595079032 | 6.832722973 |
|  | Inverse variance weighted (multiplicative random effects) | 6 | 1.395831312 | 0.395679294 | 0.000419213 | 4.038330291 | 1.85948561 | 8.770227346 |
|  | Simple mode | 6 | 0.937277227 | 0.630403791 | 0.197220851 | 2.55302065 | 0.742068143 | 8.783444624 |
|  | Weighted mode | 6 | 0.98619933 | 0.744540534 | 0.242622556 | 2.68102539 | 0.623067688 | 11.53630221 |
| Frequency of Friend/Family Visits | MR Egger | 7 | 3.377519562 | 2.952464914 | 0.304432028 | 29.29800902 | 0.089877138 | 9550.519153 |
|  | Weighted median | 7 | -0.73223993 | 0.317795138 | 0.021215591 | 0.480830756 | 0.257916755 | 0.896406345 |
|  | Inverse variance weighted (multiplicative random effects) | 7 | -0.878832649 | 0.32686933 | 0.00717446 | 0.415267392 | 0.218822028 | 0.788069683 |
|  | Simple mode | 7 | -0.510790295 | 0.449290216 | 0.298944327 | 0.600021197 | 0.248727062 | 1.447471917 |
|  | Weighted mode | 7 | -0.499161597 | 0.402175493 | 0.260871466 | 0.60703939 | 0.275980363 | 1.335228406 |
| Physical Activity | MR Egger | 4 | 0.061920436 | 0.20795344 | 0.793968391 | 1.063877695 | 0.707747204 | 1.599209075 |
|  | Weighted median | 4 | 0.007280618 | 0.02588096 | 0.778471302 | 1.007307186 | 0.957484196 | 1.059722732 |
|  | Inverse variance weighted (fixed effects) | 4 | -0.002838419 | 0.021151497 | 0.893248619 | 0.997165606 | 0.95667136 | 1.039373903 |
|  | Simple mode | 4 | 0.034893166 | 0.046250961 | 0.505408958 | 1.035509075 | 0.945767305 | 1.133766244 |
|  | Weighted mode | 4 | 0.023572623 | 0.043091193 | 0.62241699 | 1.023852653 | 0.94093039 | 1.114082684 |
| Smoking History | MR Egger | 28 | -0.467007159 | 0.385260792 | 0.236342529 | 0.626875603 | 0.294605439 | 1.333896014 |
|  | Weighted median | 28 | -0.172723247 | 0.13127414 | 0.188259763 | 0.841370434 | 0.650495719 | 1.088253445 |
|  | Inverse variance weighted (fixed effects) | 28 | -0.247153097 | 0.083937 | 0.003234695 | 0.781021113 | 0.662543133 | 0.920685686 |
|  | Simple mode | 28 | -0.164507555 | 0.296475665 | 0.583546738 | 0.848311348 | 0.474449612 | 1.516772542 |
|  | Weighted mode | 28 | -0.112684296 | 0.213259289 | 0.601544613 | 0.893432676 | 0.588209265 | 1.357037358 |
| Alcohol Intake Frequency | MR Egger | 51 | -0.461751339 | 0.392441746 | 0.245035132 | 0.630179021 | 0.292018781 | 1.359931707 |
|  | Weighted median | 51 | 0.024362761 | 0.11564176 | 0.833141334 | 1.024661958 | 0.816853848 | 1.285336575 |
|  | Inverse variance weighted (multiplicative random effects) | 51 | 0.110076974 | 0.102409304 | 0.28243169 | 1.116363998 | 0.91334165 | 1.364515213 |
|  | Simple mode | 51 | 0.161258478 | 0.254475106 | 0.529171096 | 1.174988638 | 0.713542891 | 1.93484977 |
|  | Weighted mode | 51 | 0.076947457 | 0.190485358 | 0.687968643 | 1.079985329 | 0.743487114 | 1.568780803 |

| **Table S17. Causal effects of disease history on knee osteoarthritis risk using two sample mendelian randomization analysis** | | | | | | | | |
| --- | --- | --- | --- | --- | --- | --- | --- | --- |
| Trait | method | nsnp | β | SE | pval | or | or_lci95 | or_uci95 |
| Coronary Atherosclerosis | MR Egger | 19 | -0.053415412 | 0.049825551 | 0.298682003 | 0.947986126 | 0.859784509 | 1.045235969 |
|  | Weighted median | 19 | -0.045224804 | 0.031474591 | 0.150755759 | 0.955782594 | 0.89860205 | 1.016601695 |
|  | Inverse variance weighted (fixed effects) | 19 | -0.033190774 | 0.022436251 | 0.139050292 | 0.967353996 | 0.925736331 | 1.010842637 |
|  | Simple mode | 19 | -0.017783852 | 0.042796824 | 0.682657315 | 0.982373347 | 0.90333155 | 1.068331326 |
|  | Weighted mode | 19 | -0.033463871 | 0.032227591 | 0.312850282 | 0.967089851 | 0.907891911 | 1.030147717 |
| Hypertension | MR Egger | 51 | 0.002495511 | 0.096338667 | 0.979439495 | 1.002498628 | 0.830001081 | 1.210846011 |
|  | Weighted median | 51 | -0.015665688 | 0.032312765 | 0.627808142 | 0.984456381 | 0.92404112 | 1.048821687 |
|  | Inverse variance weighted (multiplicative random effects) | 51 | -0.038562805 | 0.028582807 | 0.1772853 | 0.962171273 | 0.909750305 | 1.017612805 |
|  | Simple mode | 51 | 0.001983545 | 0.066035217 | 0.976156575 | 1.001985514 | 0.880341411 | 1.140438195 |
|  | Weighted mode | 51 | 0.006096458 | 0.057479097 | 0.915956044 | 1.006115079 | 0.898918798 | 1.126094541 |
| Gastroesophageal Reflux Disease | MR Egger | 17 | 0.824234438 | 0.947802975 | 0.398207104 | 2.280134512 | 0.355774067 | 14.61324442 |
|  | Weighted median | 17 | 0.479109732 | 0.121141167 | 7.65E-05 | 1.614636303 | 1.273377786 | 2.047350299 |
|  | Inverse variance weighted (multiplicative random effects) | 17 | 0.418241955 | 0.102081824 | 4.18314E-05 | 1.51928823 | 1.243788024 | 1.855811988 |
|  | Simple mode | 17 | 0.567342882 | 0.217922953 | 0.019207278 | 1.763574794 | 1.150519862 | 2.703296272 |
|  | Weighted mode | 17 | 0.542217015 | 0.224252967 | 0.027907234 | 1.719815495 | 1.108138077 | 2.669130679 |
| Hypercholesterolemia | MR Egger | 4 | -0.951611737 | 0.998955566 | 0.441328156 | 0.386118201 | 0.054499451 | 2.735573689 |
|  | Weighted median | 4 | -0.91398424 | 0.32439519 | 0.004839875 | 0.400923661 | 0.21229068 | 0.757168343 |
|  | Inverse variance weighted (fixed effects) | 4 | -0.696585117 | 0.27545452 | 0.011443461 | 0.498283983 | 0.290405814 | 0.854965418 |
|  | Simple mode | 4 | -0.989230611 | 0.531336133 | 0.159548127 | 0.371862688 | 0.131250255 | 1.053574018 |
|  | Weighted mode | 4 | -1.021817112 | 0.461520453 | 0.113674326 | 0.359940294 | 0.145672082 | 0.889374364 |
| Type 2 Diabetes | MR Egger | 30 | 0.00753645 | 0.170595607 | 0.965076648 | 1.00756492 | 0.72120566 | 1.407624932 |
|  | Weighted median | 30 | -0.063982555 | 0.056181411 | 0.254763084 | 0.938021363 | 0.840214452 | 1.047213691 |
|  | Inverse variance weighted (fixed effects) | 30 | -0.02761466 | 0.037044294 | 0.455999879 | 0.972763139 | 0.90463704 | 1.046019656 |
|  | Simple mode | 30 | -0.084834658 | 0.102415632 | 0.414245149 | 0.918664165 | 0.751586257 | 1.122883556 |
|  | Weighted mode | 30 | -0.059347069 | 0.091311728 | 0.520847167 | 0.942379642 | 0.787952039 | 1.127072899 |
| Hypothyroidism | MR Egger | 61 | 0.052398434 | 0.03680238 | 0.159777882 | 1.053795527 | 0.980459216 | 1.132617242 |
|  | Weighted median | 61 | 0.02889745 | 0.02023497 | 0.153264646 | 1.029319033 | 0.989294624 | 1.070962729 |
|  | Inverse variance weighted (multiplicative random effects) | 61 | 0.046624093 | 0.017178307 | 0.006645086 | 1.047728087 | 1.013038885 | 1.083605141 |
|  | Simple mode | 61 | 0.043582923 | 0.042651752 | 0.310962892 | 1.044546608 | 0.960775497 | 1.135621817 |
|  | Weighted mode | 61 | 0.041129286 | 0.020542814 | 0.049797661 | 1.041986811 | 1.000865745 | 1.084797356 |

| **Table S18. Causal effects of drug history on knee osteoarthritis risk using two sample mendelian randomization analysis** | | | | | | | | |
| --- | --- | --- | --- | --- | --- | --- | --- | --- |
| Trait | method | nsnp | β | SE | pval | or | or_lci95 | or_uci95 |
| Antithrombotic Agents | MR Egger | 23 | -0.128035621 | 0.14923594 | 0.400611298 | 0.879822039 | 0.656693382 | 1.178764461 |
|  | Weighted median | 23 | -0.14212847 | 0.062861083 | 0.02375969 | 0.8675098 | 0.766948064 | 0.981257127 |
|  | Inverse variance weighted (fixed effects) | 23 | -0.11408165 | 0.045847846 | 0.012836757 | 0.892185106 | 0.815508505 | 0.976071076 |
|  | Simple mode | 23 | -0.184266008 | 0.098183019 | 0.073877842 | 0.831714532 | 0.686118638 | 1.008206196 |
|  | Weighted mode | 23 | -0.166769098 | 0.079064702 | 0.046530329 | 0.846395023 | 0.724889598 | 0.988267091 |
| Beta Blocking Agents | MR Egger | 48 | -0.121315924 | 0.122838972 | 0.328514481 | 0.885754084 | 0.696226452 | 1.126875165 |
|  | Weighted median | 48 | -0.052239207 | 0.044080165 | 0.235979673 | 0.949101808 | 0.870544561 | 1.034747997 |
|  | Inverse variance weighted (multiplicative random effects) | 48 | -0.046216093 | 0.034888343 | 0.185275388 | 0.954835607 | 0.891725217 | 1.02241253 |
|  | Simple mode | 48 | -0.048867422 | 0.090535388 | 0.591910543 | 0.952307377 | 0.797465439 | 1.137214599 |
|  | Weighted mode | 48 | -0.125986688 | 0.087068213 | 0.154537333 | 0.881626583 | 0.743311269 | 1.045679601 |
| Antihypertensives | MR Egger | 5 | 0.553730919 | 0.259856102 | 0.122904826 | 1.739731722 | 1.045413978 | 2.895184616 |
|  | Weighted median | 5 | -0.006567696 | 0.080427941 | 0.93491758 | 0.993453824 | 0.848566809 | 1.163079313 |
|  | Inverse variance weighted (multiplicative random effects) | 5 | -0.050526876 | 0.093884753 | 0.590452936 | 0.950728377 | 0.790933812 | 1.142806683 |
|  | Simple mode | 5 | 0.003919406 | 0.152055025 | 0.980670501 | 1.003927097 | 0.745195675 | 1.352489888 |
|  | Weighted mode | 5 | 0.029142011 | 0.107988691 | 0.800616892 | 1.029570795 | 0.833171461 | 1.27226636 |
| Drugs for Peptic Ulcer and Gastro-Oesophageal Reflux Disease | MR Egger | 3 | 1.473341647 | 1.335813762 | 0.468857856 | 4.363793061 | 0.318270596 | 59.83175985 |
|  | Weighted median | 3 | 0.037777926 | 0.171262849 | 0.825415989 | 1.038500583 | 0.742377609 | 1.452742442 |
|  | Inverse variance weighted (fixed effects) | 3 | -0.0254861 | 0.138780245 | 0.854293081 | 0.97483593 | 0.742676014 | 1.279568845 |
|  | Simple mode | 3 | 0.081513407 | 0.236774051 | 0.763474099 | 1.084927763 | 0.682110417 | 1.725627143 |
|  | Weighted mode | 3 | 0.076603641 | 0.202348388 | 0.741413168 | 1.079614077 | 0.726149618 | 1.605132779 |
| Statin Medication | MR Egger | 47 | -0.065232893 | 0.034788609 | 0.067274067 | 0.936849252 | 0.875098728 | 1.002957145 |
|  | Weighted median | 47 | -0.0287023 | 0.026966241 | 0.287157044 | 0.971705698 | 0.921680972 | 1.024445542 |
|  | Inverse variance weighted (multiplicative random effects) | 47 | -0.045106952 | 0.022088183 | 0.041138588 | 0.955895241 | 0.915394837 | 0.998187531 |
|  | Simple mode | 47 | 0.010702446 | 0.064228281 | 0.86839042 | 1.010759922 | 0.89120127 | 1.146357905 |
|  | Weighted mode | 47 | -0.048653548 | 0.028571602 | 0.095342474 | 0.952511072 | 0.90063619 | 1.007373845 |
| Drugs Used in Diabetes | MR Egger | 73 | -0.018398546 | 0.061975685 | 0.767434672 | 0.981769674 | 0.869470487 | 1.108573214 |
|  | Weighted median | 73 | 0.051771154 | 0.029287315 | 0.077111253 | 1.05313471 | 0.994383847 | 1.11535673 |
|  | Inverse variance weighted (fixed effects) | 73 | 0.03046998 | 0.019439823 | 0.117021215 | 1.030938941 | 0.992396976 | 1.070977768 |
|  | Simple mode | 73 | 0.017291247 | 0.059462368 | 0.772045556 | 1.017441606 | 0.905511779 | 1.143207019 |
|  | Weighted mode | 73 | 0.050520302 | 0.043831107 | 0.252881752 | 1.051818218 | 0.965230189 | 1.146173809 |
| Thyroid Preparations | MR Egger | 103 | 0.080917082 | 0.029818207 | 0.007825065 | 1.084280986 | 1.022727825 | 1.149538742 |
|  | Weighted median | 103 | 0.055779876 | 0.020204041 | 0.00576551 | 1.057364906 | 1.016311559 | 1.100076581 |
|  | Inverse variance weighted (multiplicative random effects) | 103 | 0.041323064 | 0.013911384 | 0.002973643 | 1.042188744 | 1.014156012 | 1.070996342 |
|  | Simple mode | 103 | 0.078111061 | 0.044203516 | 0.080206119 | 1.081242736 | 0.991508428 | 1.179098251 |
|  | Weighted mode | 103 | 0.078111061 | 0.028000239 | 0.006297471 | 1.081242736 | 1.023502525 | 1.142240321 |

| **Table S19. Sensitivity analyses (daily habits)** | | | |
| --- | --- | --- | --- |
| Exposures | method | Cochran’s Q test pval | MR-PRESSO test pval |
| Time Spent Watching Television (TV) | MR Egger | 0.079915166 | 0.229667719 |
|  | Inverse variance weighted | 0.028313816 |  |
| Frequency of Friend/Family Visits | MR Egger | 0.085004734 | 0.206973147 |
|  | Inverse variance weighted | 0.032707288 |  |
| Physical Activity | MR Egger | 0.050317125 | 0.781708036 |
|  | Inverse variance weighted | 0.098842178 |  |
| Smoking History | MR Egger | 0.131799314 | 0.560734192 |
|  | Inverse variance weighted | 0.149687805 |  |
| Alcohol Intake Frequency | MR Egger | 2.41322E-07 | 0.137966843 |
|  | Inverse variance weighted | 7.41728E-08 |  |

| **Table S20. Sensitivity analyses (disease history)** | | | |
| --- | --- | --- | --- |
| Exposures | method | Cochran’s Q test pval | MR-PRESSO test pval |
| Coronary Atherosclerosis | MR Egger | 0.22045847 | 0.644787168 |
|  | Inverse variance weighted | 0.259453919 |  |
| Hypertension | MR Egger | 0.000189904 | 0.657108103 |
|  | Inverse variance weighted | 0.000243096 |  |
| Gastroesophageal Reflux Disease | MR Egger | 0.028176245 | 0.672557005 |
|  | Inverse variance weighted | 0.037198439 |  |
| Hypercholesterolemia | MR Egger | 0.26325862 | 0.812921617 |
|  | Inverse variance weighted | 0.429120283 |  |
| Type 2 Diabetes | MR Egger | 0.214221895 | 0.83353518 |
|  | Inverse variance weighted | 0.252005808 |  |
| Hypothyroidism | MR Egger | 0.028176245 | 0.672557005 |
|  | Inverse variance weighted | 0.037198439 |  |

| **Table S21. Sensitivity analyses (drug history)** | | | |
| --- | --- | --- | --- |
| Exposures | method | Cochran’s Q test pval | MR-PRESSO test pval |
| Antithrombotic Agents | MR Egger | 0.093071709 | 0.920892062 |
|  | Inverse variance weighted | 0.119421734 |  |
| Beta Blocking Agents | MR Egger | 0.012898142 | 0.526647609 |
|  | Inverse variance weighted | 0.014590216 |  |
| Antihypertensives | MR Egger | 0.255654227 | 0.096047092 |
|  | Inverse variance weighted | 0.018695182 |  |
| Drugs for Peptic Ulcer and Gastro-Oesophageal Reflux Disease | MR Egger | 0.171919059 | 0.460214515 |
|  | Inverse variance weighted | 0.118611484 |  |
| Statin Medication | MR Egger | 0.000109824 | 0.456411417 |
|  | Inverse variance weighted | 0.000118048 |  |
| Drugs Used in Diabetes | MR Egger | 0.078921141 | 0.402742997 |
|  | Inverse variance weighted | 0.081458087 |  |
| Thyroid Preparations | MR Egger | 0.00264366 | 0.13706393 |
|  | Inverse variance weighted | 0.001861698 |  |

| **Figure S1. Visualization of mendelian randomization analysis results about time spent watching television (exposure) - knee osteoarthritis (outcome)** |
| --- |
| 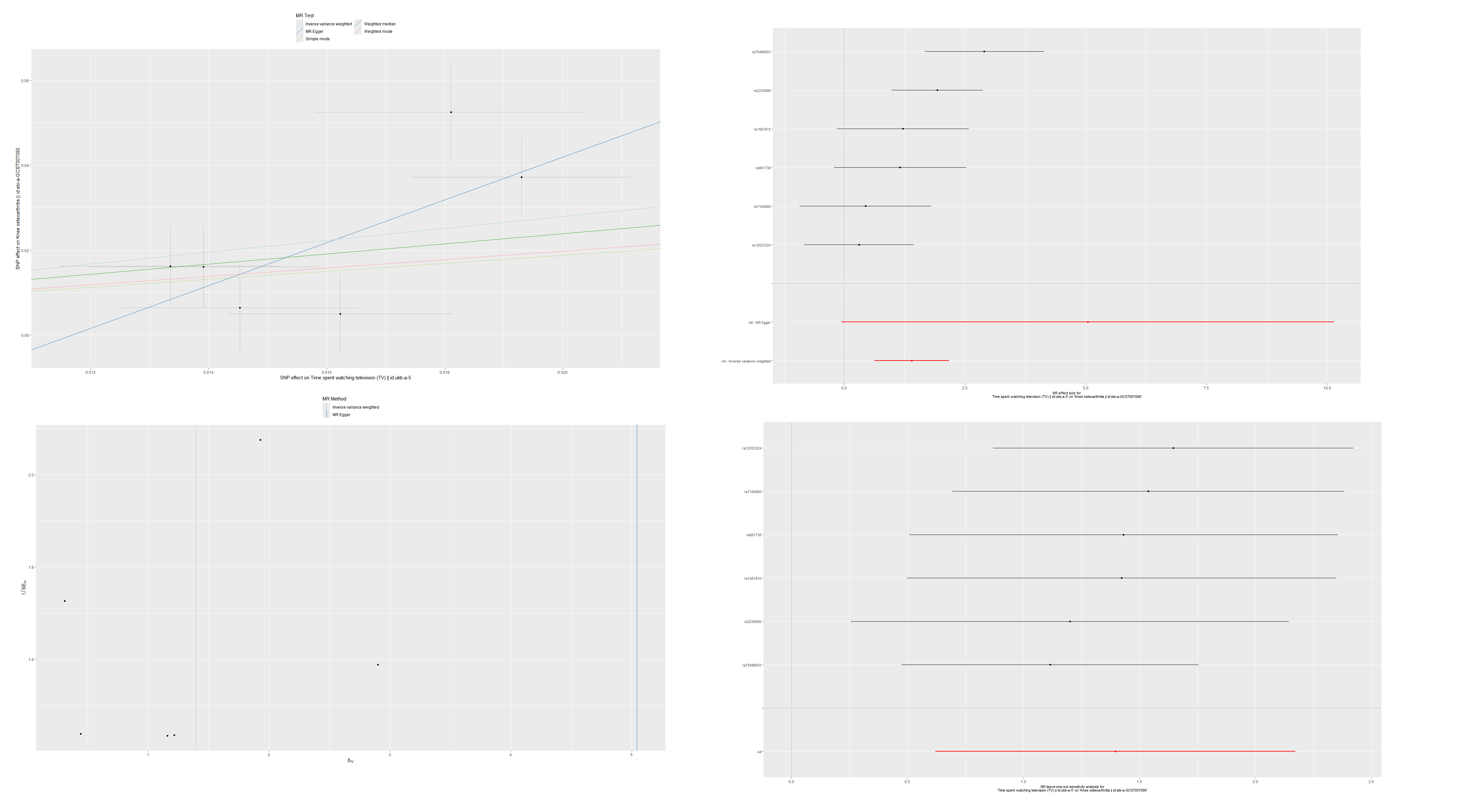 |
| Top left: scatter diagram; Top right: forest map; Bottom left: funnel plot; Bottom right: sensitivity analysis chart of leave one out method |

| **Figure S2. Visualization of mendelian randomization analysis results about frequency of friend/family visits (exposure) - knee osteoarthritis (outcome)** |
| --- |
| 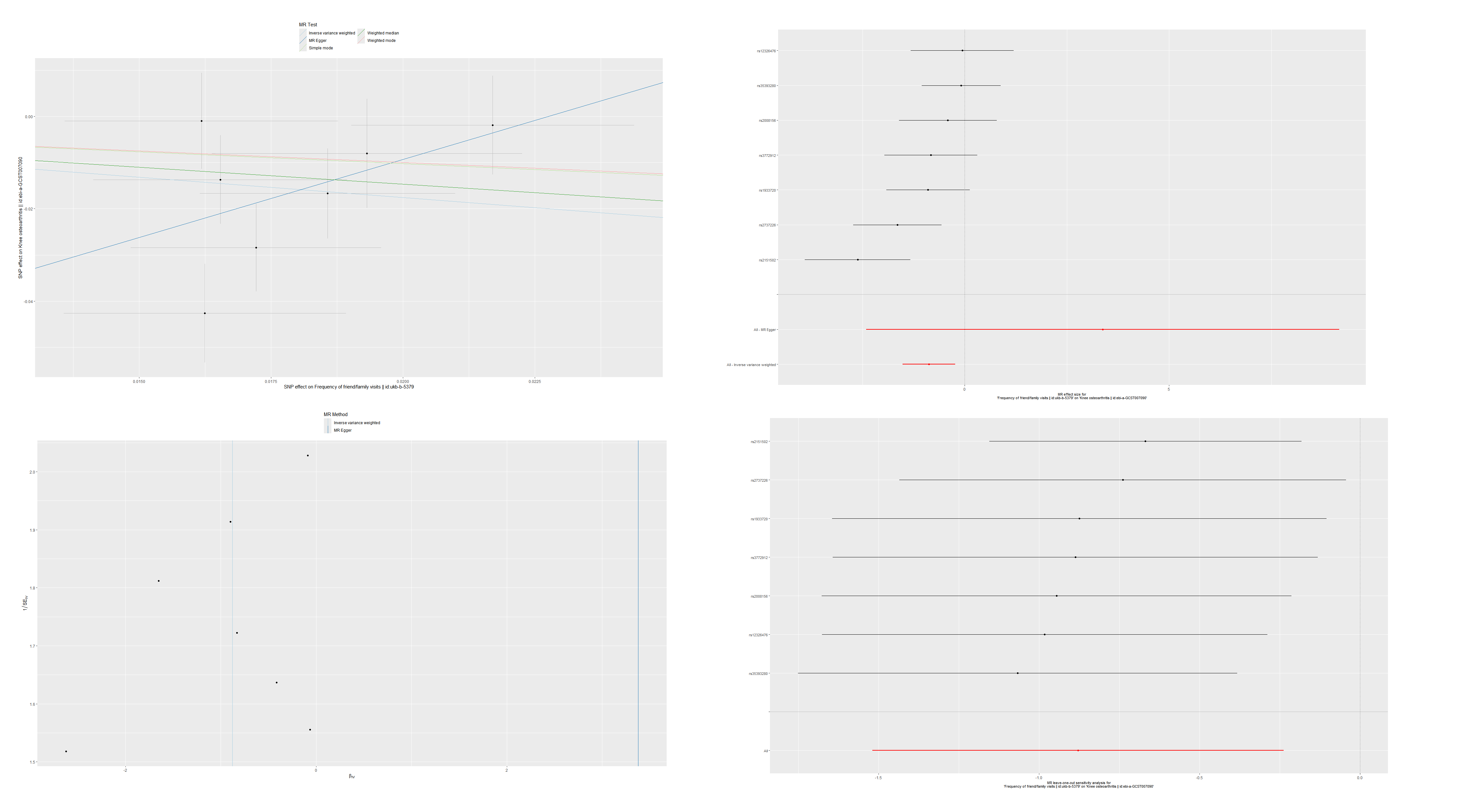 |
| Top left: scatter diagram; Top right: forest map; Bottom left: funnel plot; Bottom right: sensitivity analysis chart of leave one out method |

| **Figure S3. Visualization of mendelian randomization analysis results about physical activity (exposure) - knee osteoarthritis (outcome)** |
| --- |
| 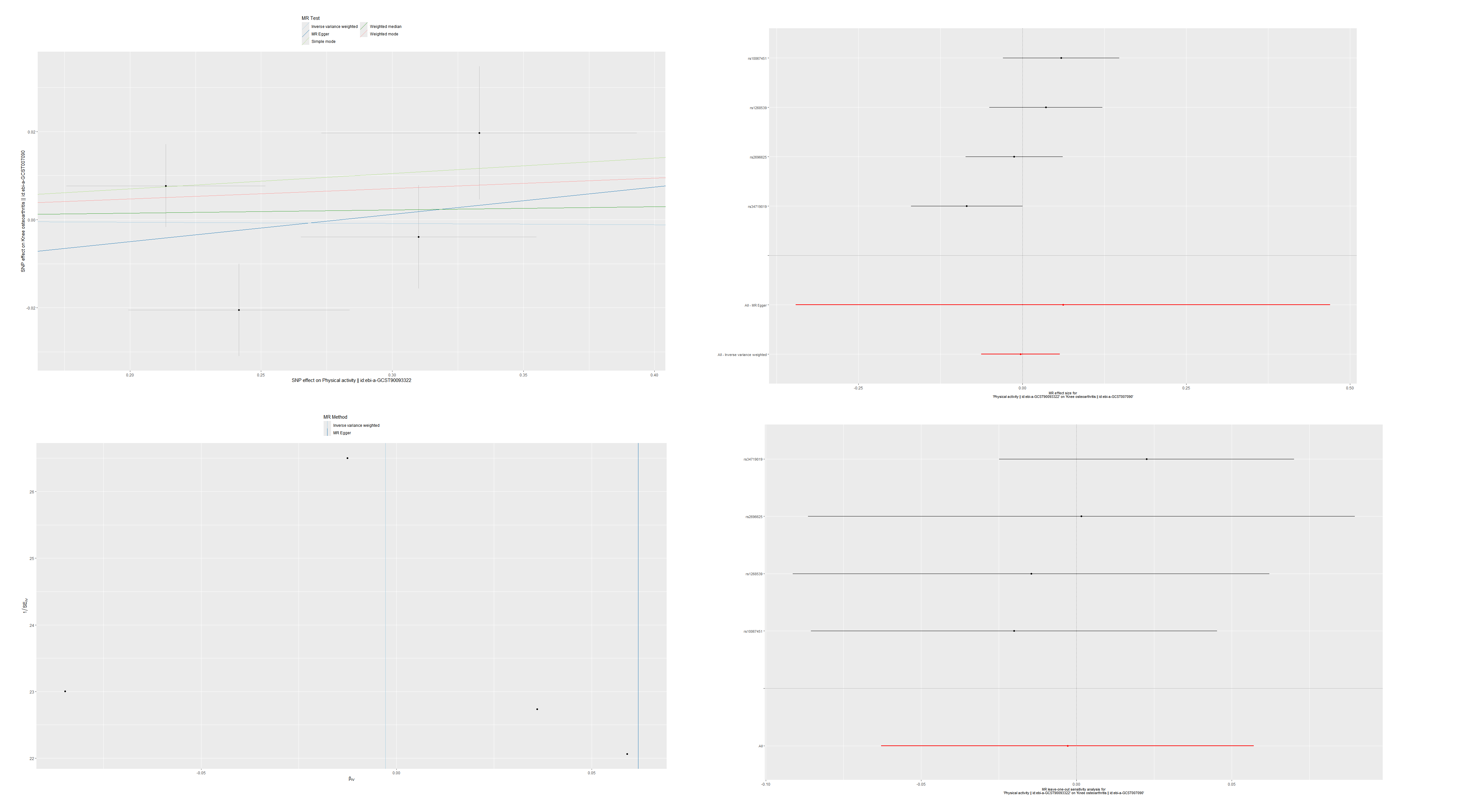 |
| Top left: scatter diagram; Top right: forest map; Bottom left: funnel plot; Bottom right: sensitivity analysis chart of leave one out method |

| **Figure S4. Visualization of mendelian randomization analysis results about smoking history (exposure) - knee osteoarthritis (outcome)** |
| --- |
| 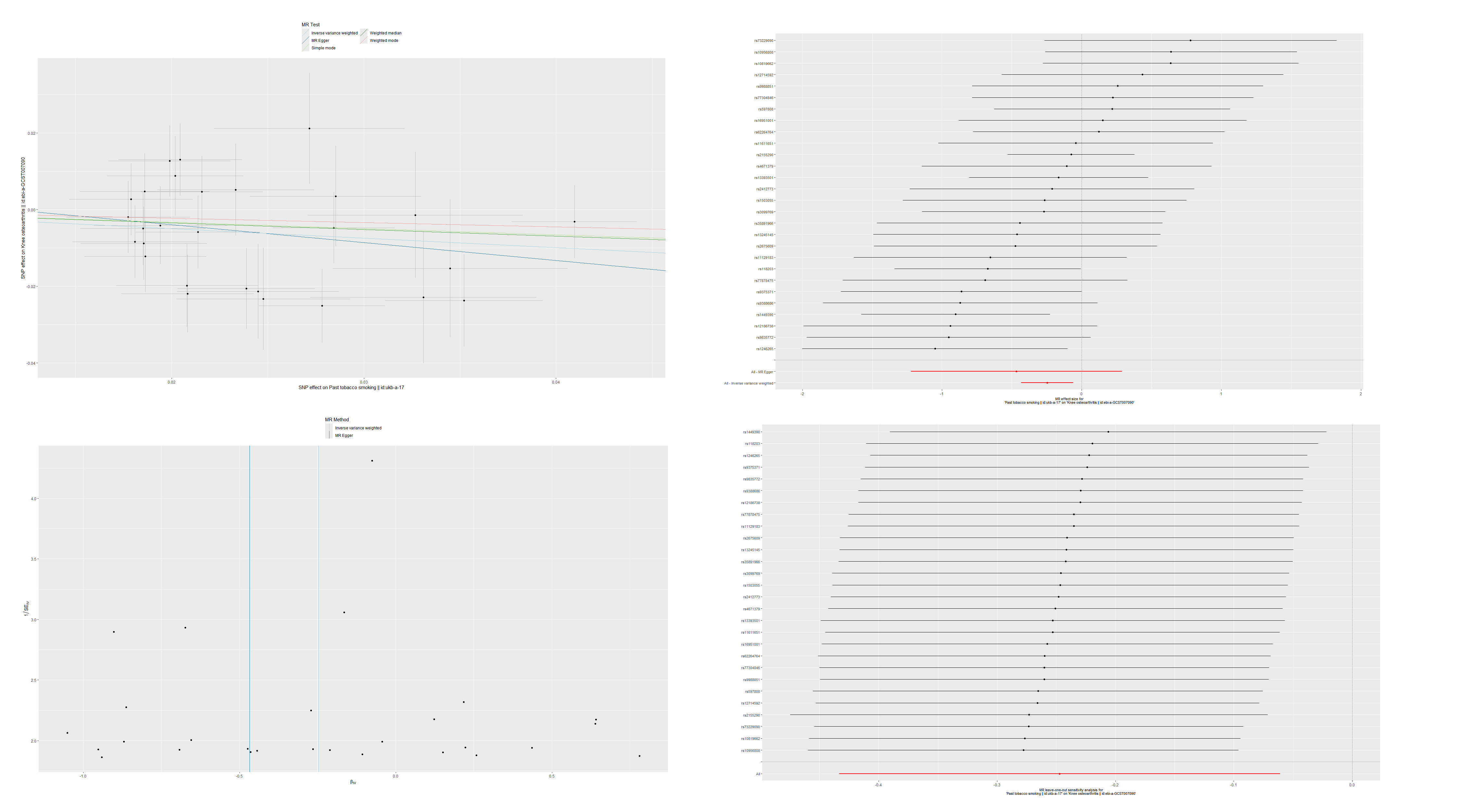 |
| Top left: scatter diagram; Top right: forest map; Bottom left: funnel plot; Bottom right: sensitivity analysis chart of leave one out method |

| **Figure S5. Visualization of mendelian randomization analysis results about alcohol intake frequency (exposure) - knee osteoarthritis (outcome)** |
| --- |
| 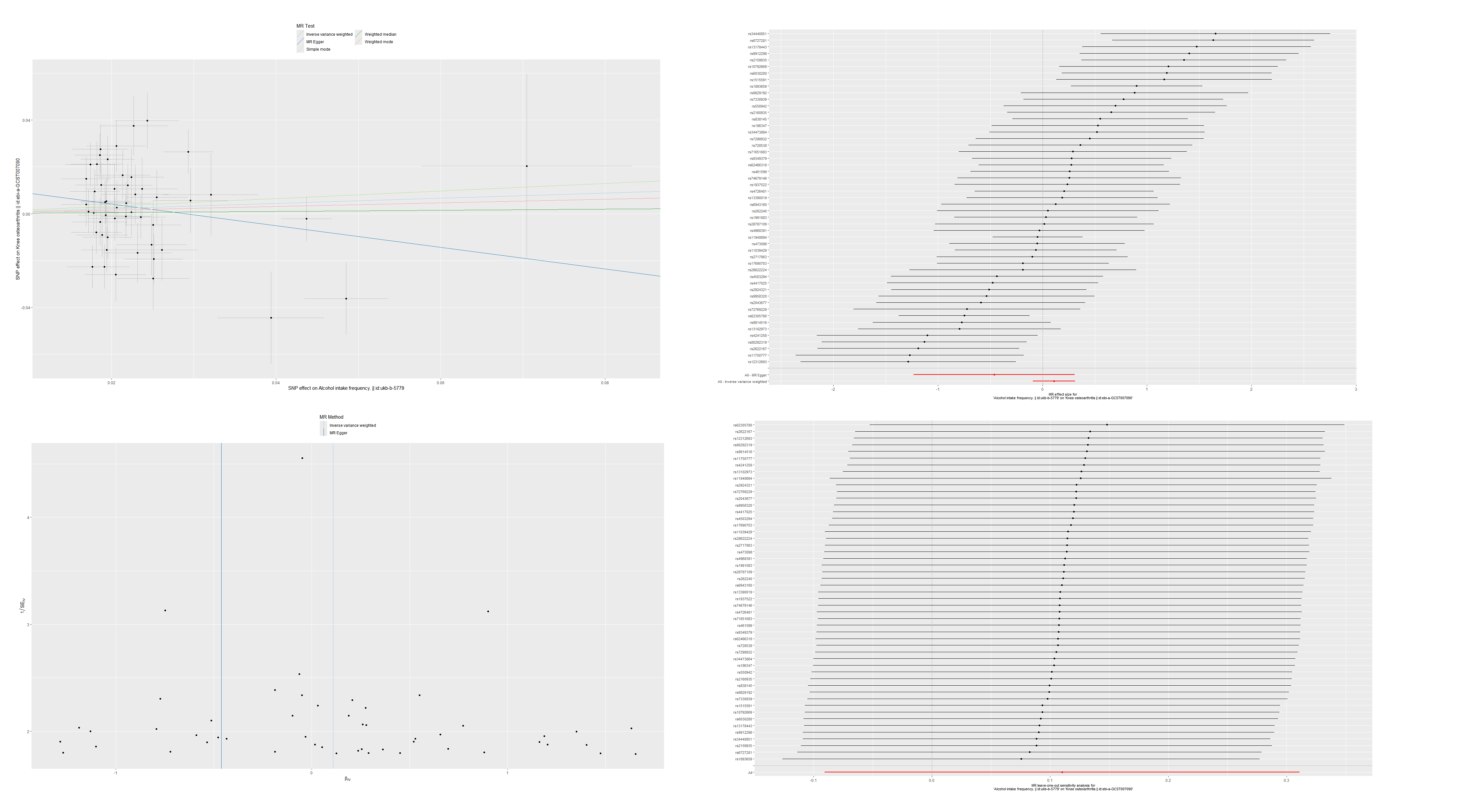 |
| Top left: scatter diagram; Top right: forest map; Bottom left: funnel plot; Bottom right: sensitivity analysis chart of leave one out method |

| **Figure S6. Visualization of mendelian randomization analysis results about coronary atherosclerosis (exposure) - knee osteoarthritis (outcome)** |
| --- |
| 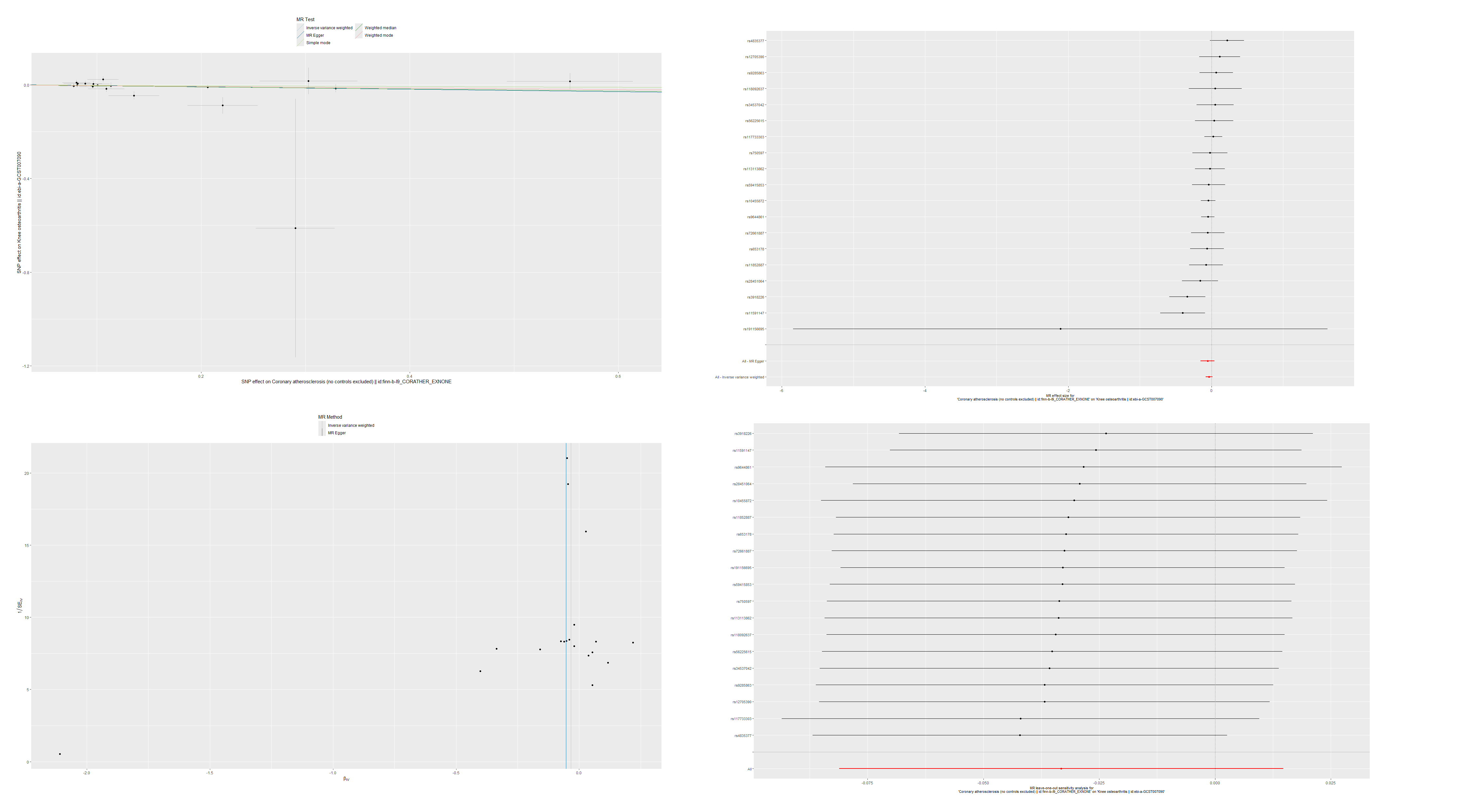 |
| Top left: scatter diagram; Top right: forest map; Bottom left: funnel plot; Bottom right: sensitivity analysis chart of leave one out method |

| **Figure S7. Visualization of mendelian randomization analysis results about hypertension (exposure) - knee osteoarthritis (outcome)** |
| --- |
| 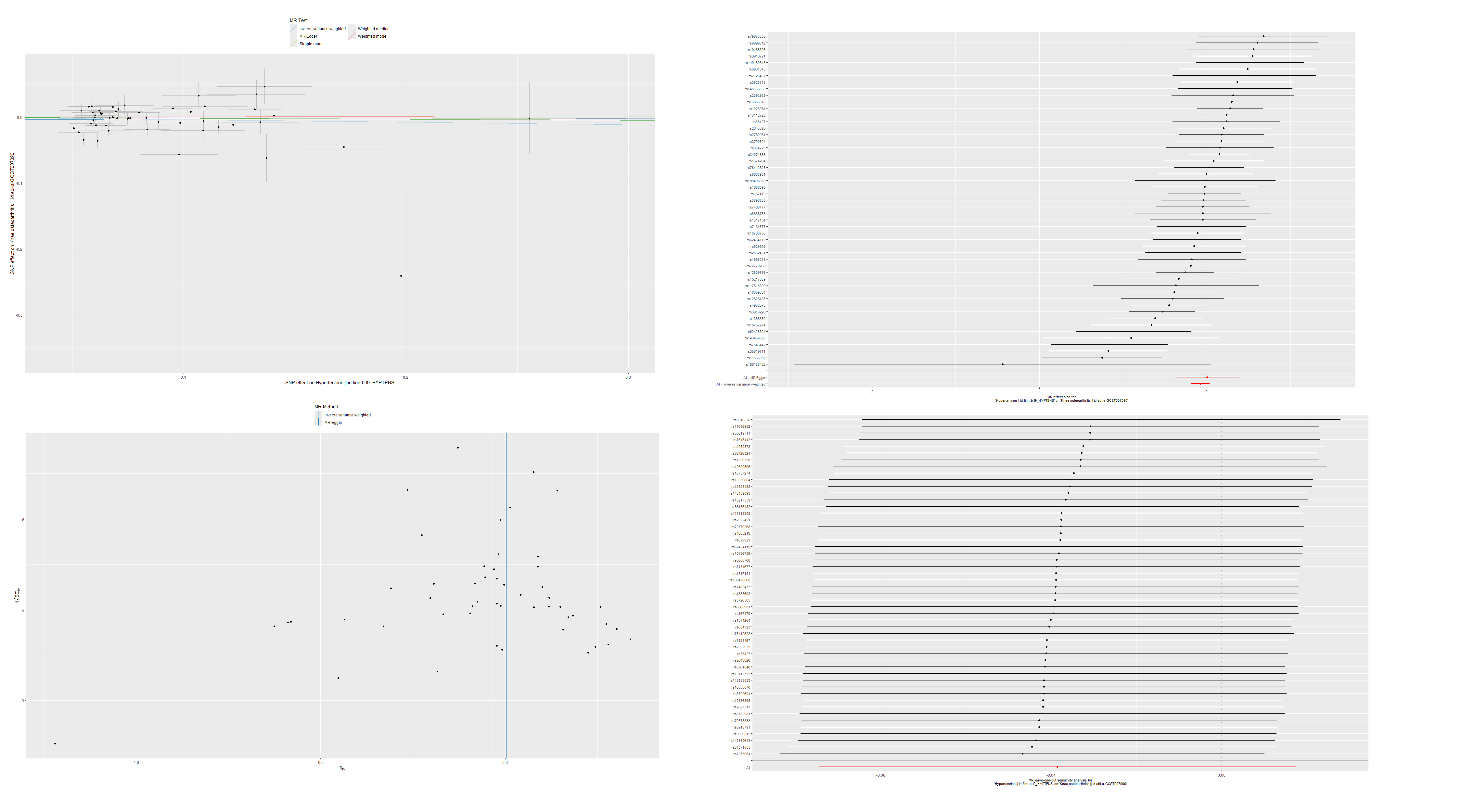 |
| Top left: scatter diagram; Top right: forest map; Bottom left: funnel plot; Bottom right: sensitivity analysis chart of leave one out method |

| **Figure S8. Visualization of mendelian randomization analysis results about gastroesophageal reflux disease (exposure) - knee osteoarthritis (outcome)** |
| --- |
| 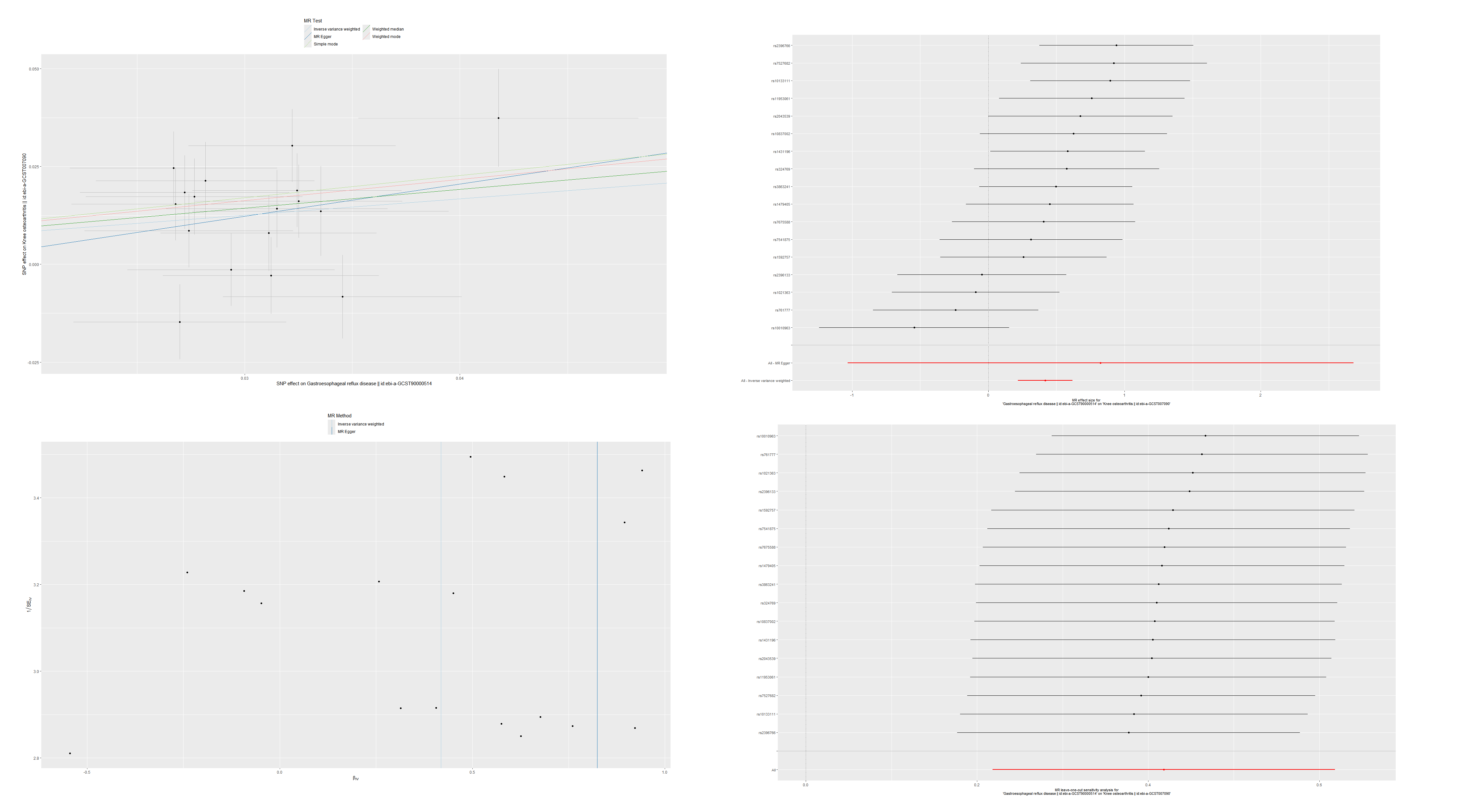 |
| Top left: scatter diagram; Top right: forest map; Bottom left: funnel plot; Bottom right: sensitivity analysis chart of leave one out method |

| **Figure S9. Visualization of mendelian randomization analysis results about hypercholesterolemia (exposure) - knee osteoarthritis (outcome)** |
| --- |
| 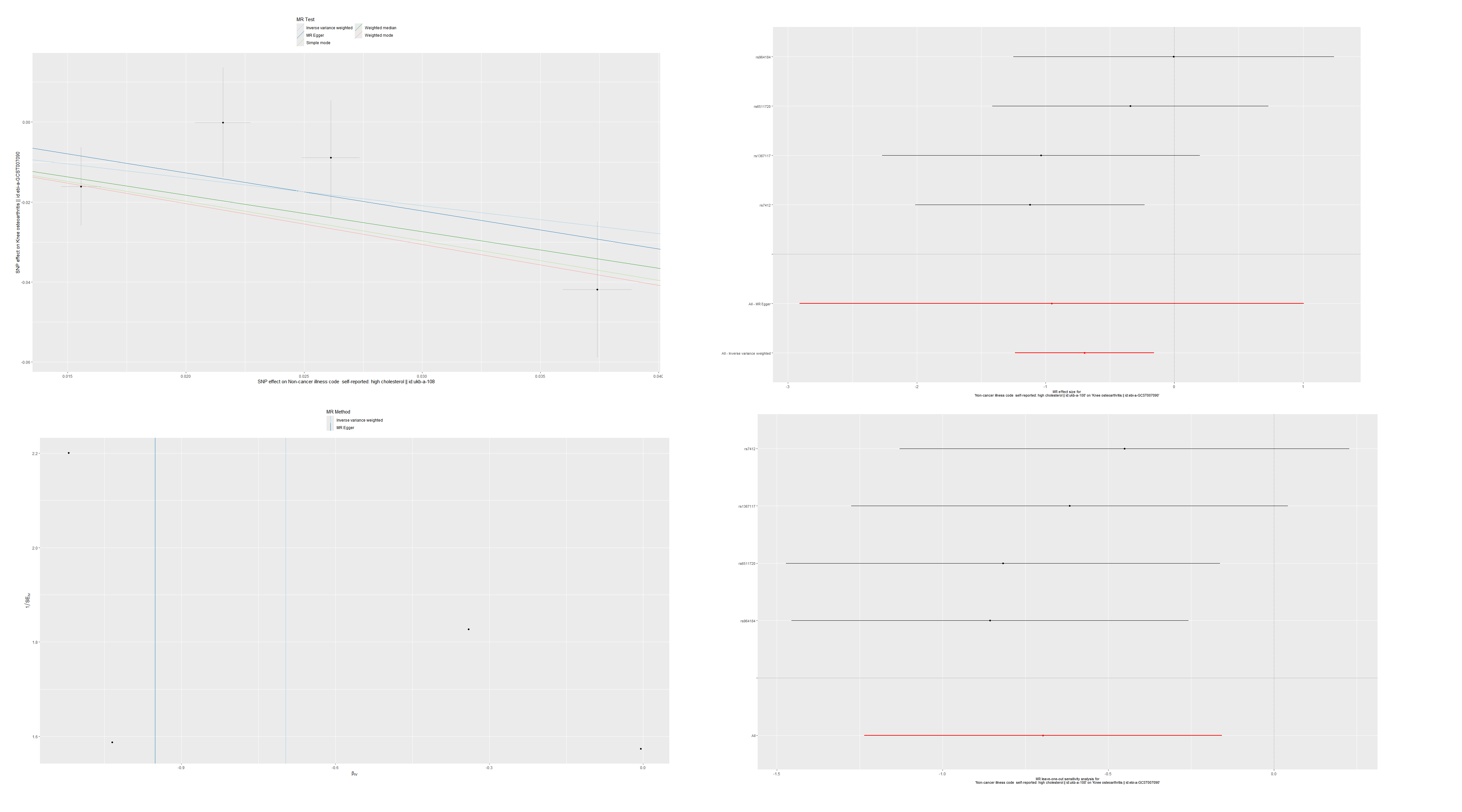 |
| Top left: scatter diagram; Top right: forest map; Bottom left: funnel plot; Bottom right: sensitivity analysis chart of leave one out method |

| **Figure S10. Visualization of mendelian randomization analysis results about type 2 diabetes (exposure) - knee osteoarthritis (outcome)** |
| --- |
| 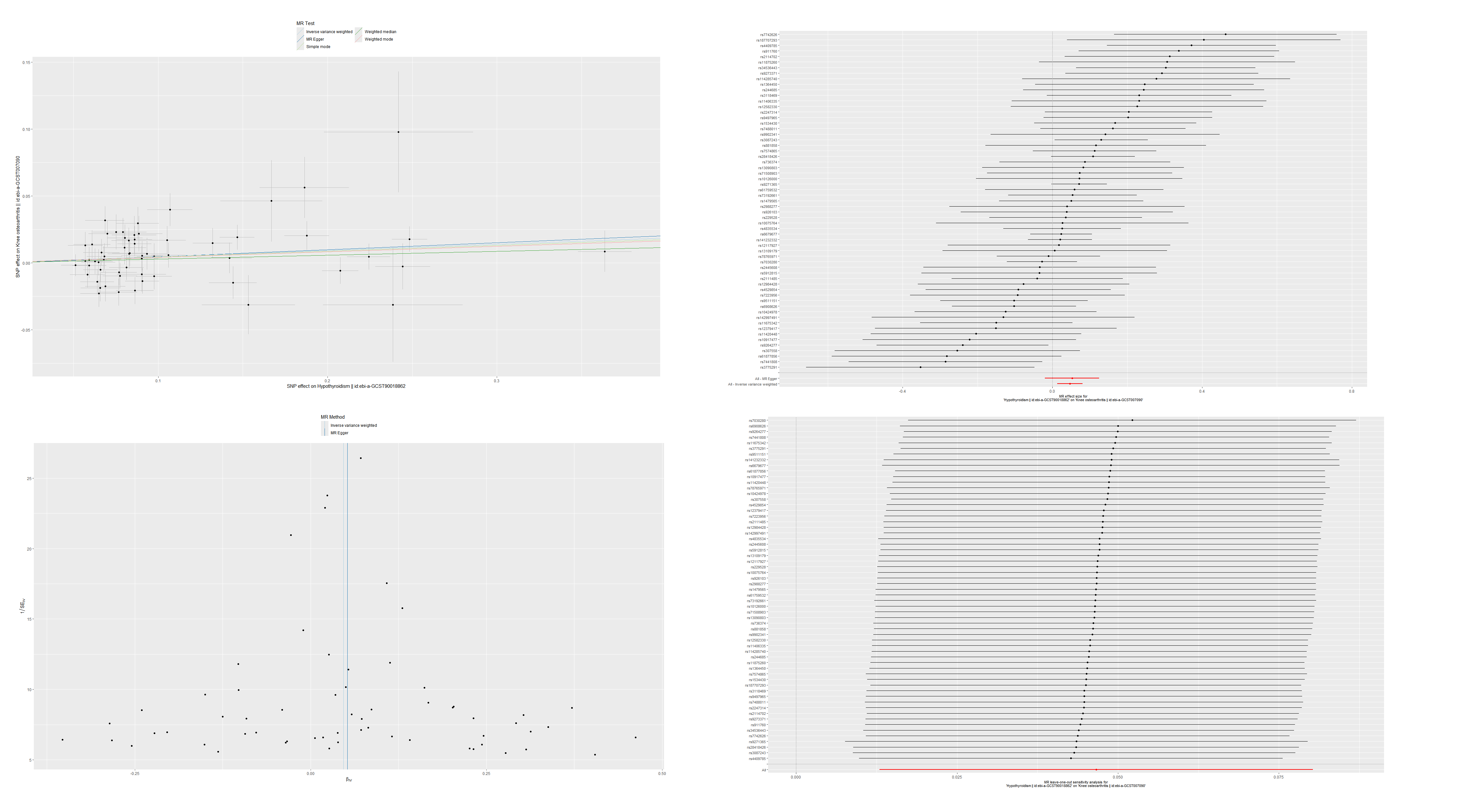 |
| Top left: scatter diagram; Top right: forest map; Bottom left: funnel plot; Bottom right: sensitivity analysis chart of leave one out method |

| **Figure S11. Visualization of mendelian randomization analysis results about antithrombotic agents (exposure) - knee osteoarthritis (outcome)** |
| --- |
| 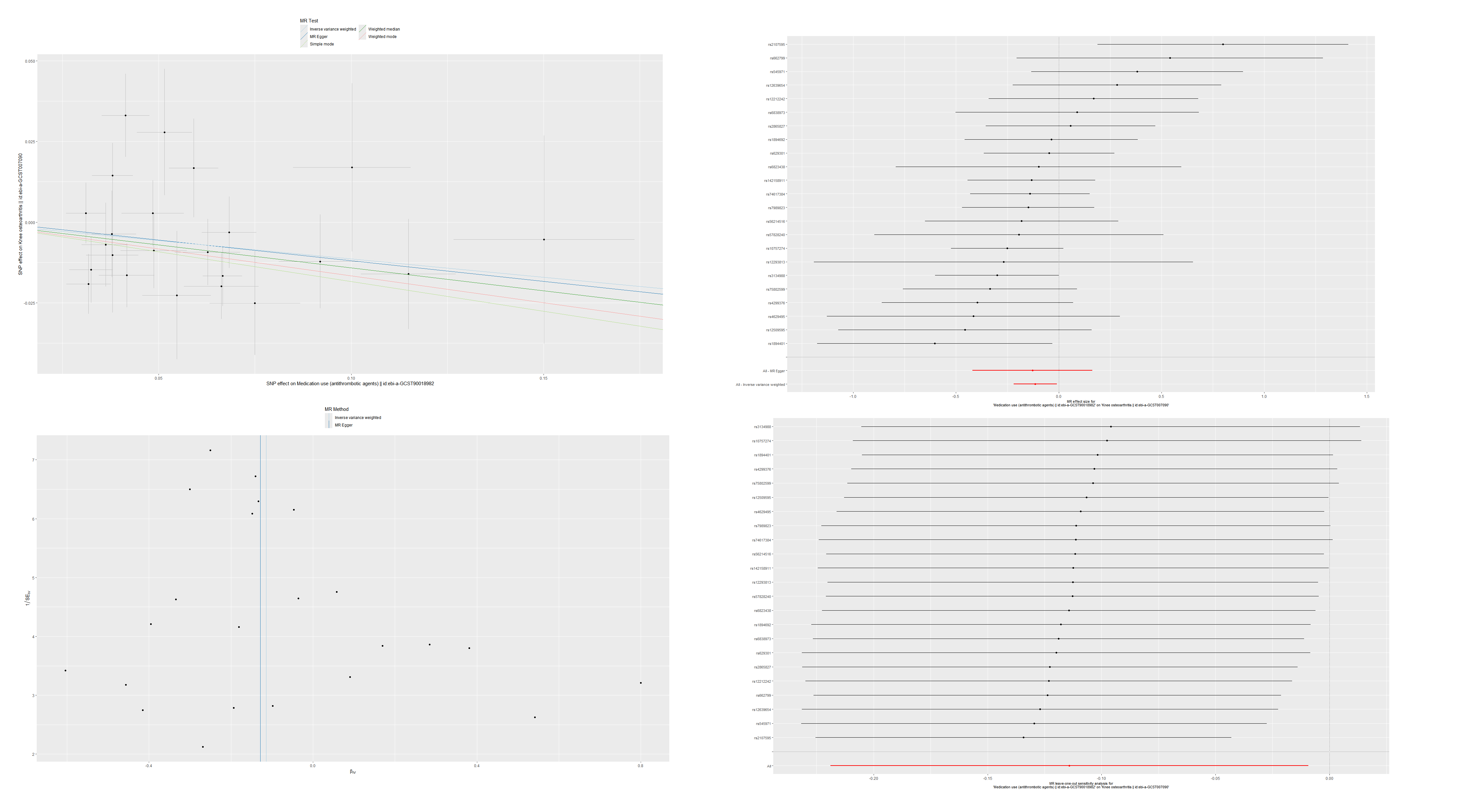 |
| Top left: scatter diagram; Top right: forest map; Bottom left: funnel plot; Bottom right: sensitivity analysis chart of leave one out method |

| **Figure S12. Visualization of mendelian randomization analysis results about beta blocking agents (exposure) - knee osteoarthritis (outcome)** |
| --- |
| 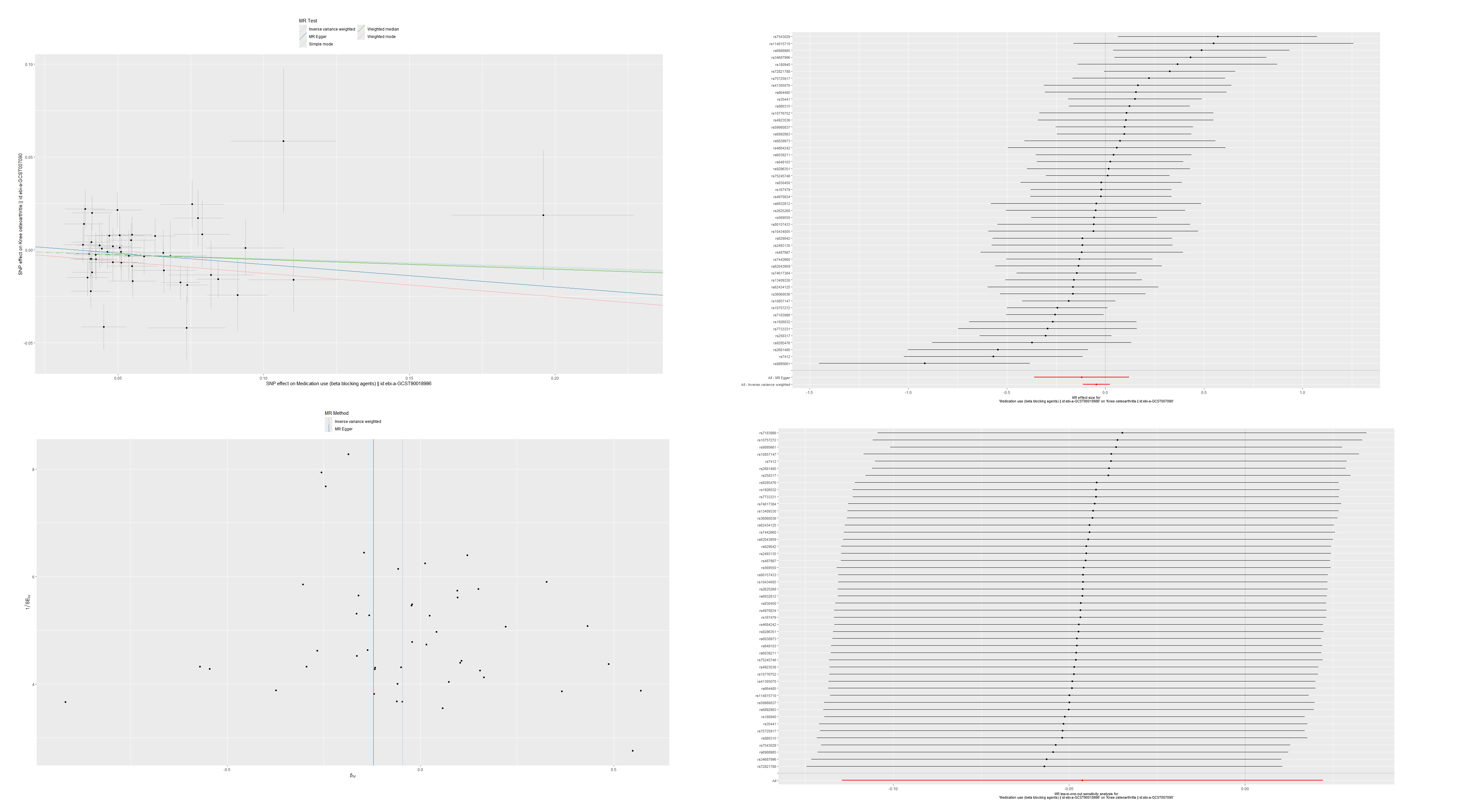 |
| Top left: scatter diagram; Top right: forest map; Bottom left: funnel plot; Bottom right: sensitivity analysis chart of leave one out method |

| **Figure S13. Visualization of mendelian randomization analysis results about antihypertensives (exposure) - knee osteoarthritis (outcome)** |
| --- |
| 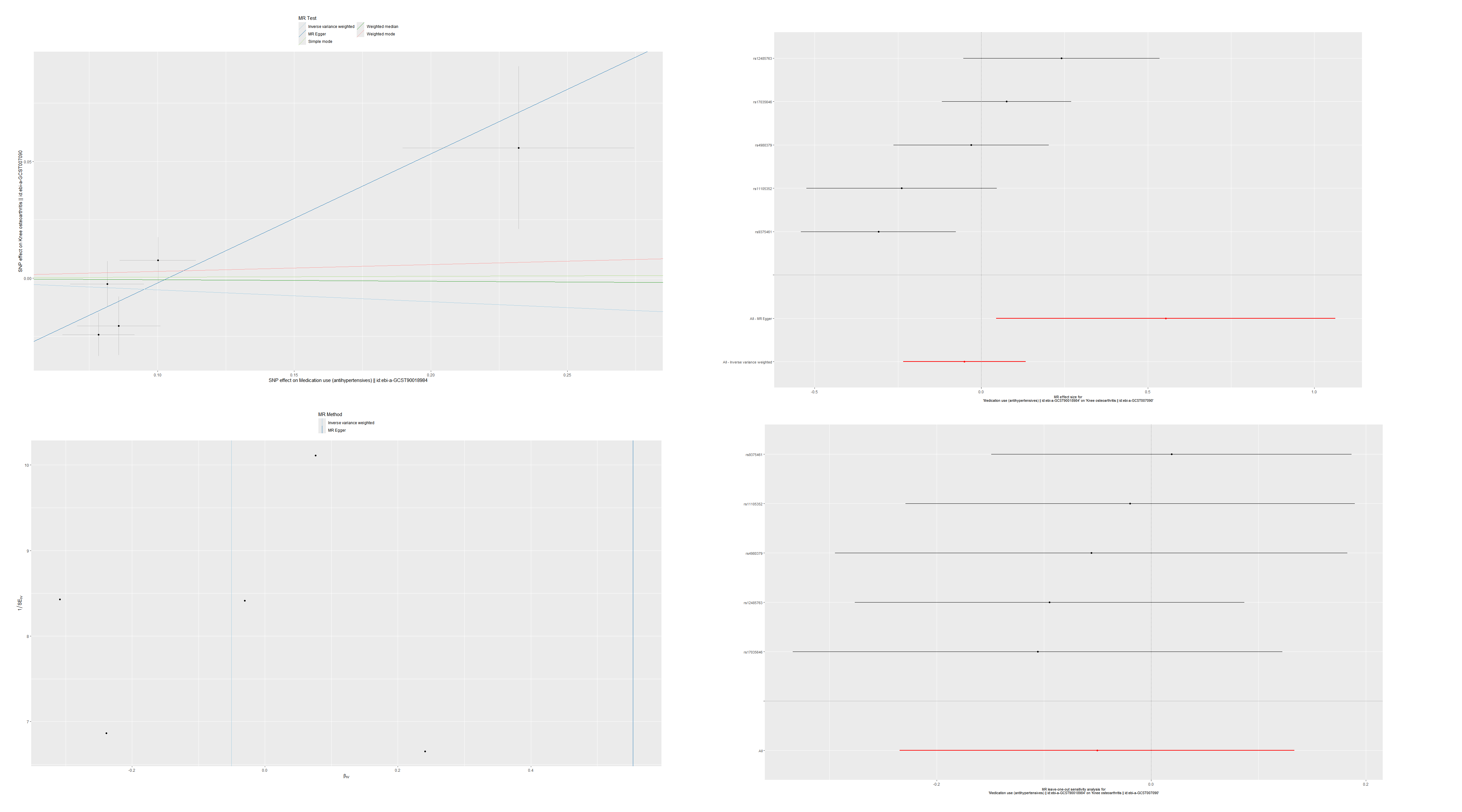 |
| Top left: scatter diagram; Top right: forest map; Bottom left: funnel plot; Bottom right: sensitivity analysis chart of leave one out method |

| **Figure S14. Visualization of mendelian randomization analysis results about drugs for peptic ulcer and gastro-oesophageal reflux disease (exposure) - knee osteoarthritis (outcome)** |
| --- |
| 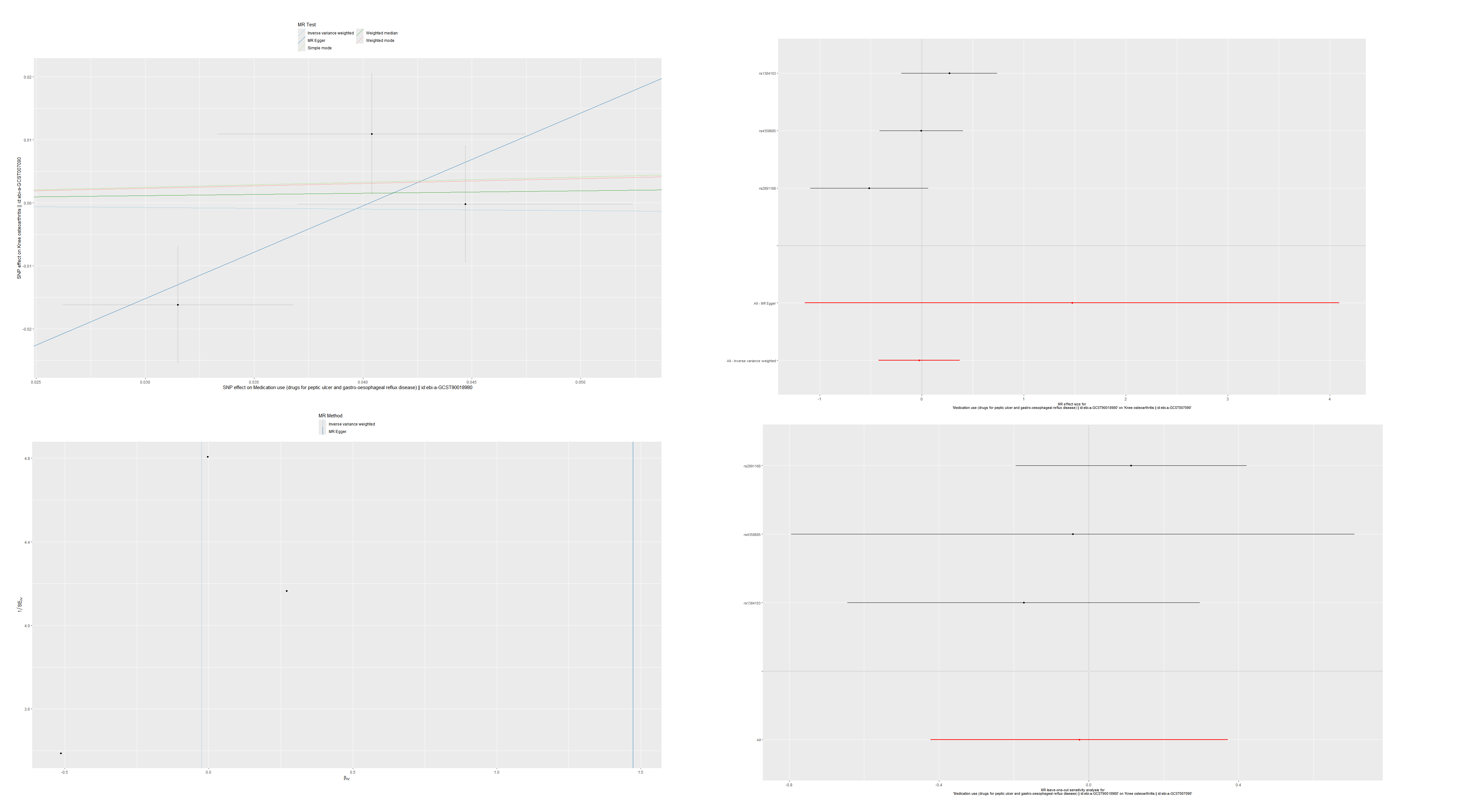 |
| Top left: scatter diagram; Top right: forest map; Bottom left: funnel plot; Bottom right: sensitivity analysis chart of leave one out method |

| **Figure S15. Visualization of mendelian randomization analysis results about statin medication (exposure) - knee osteoarthritis (outcome)** |
| --- |
| 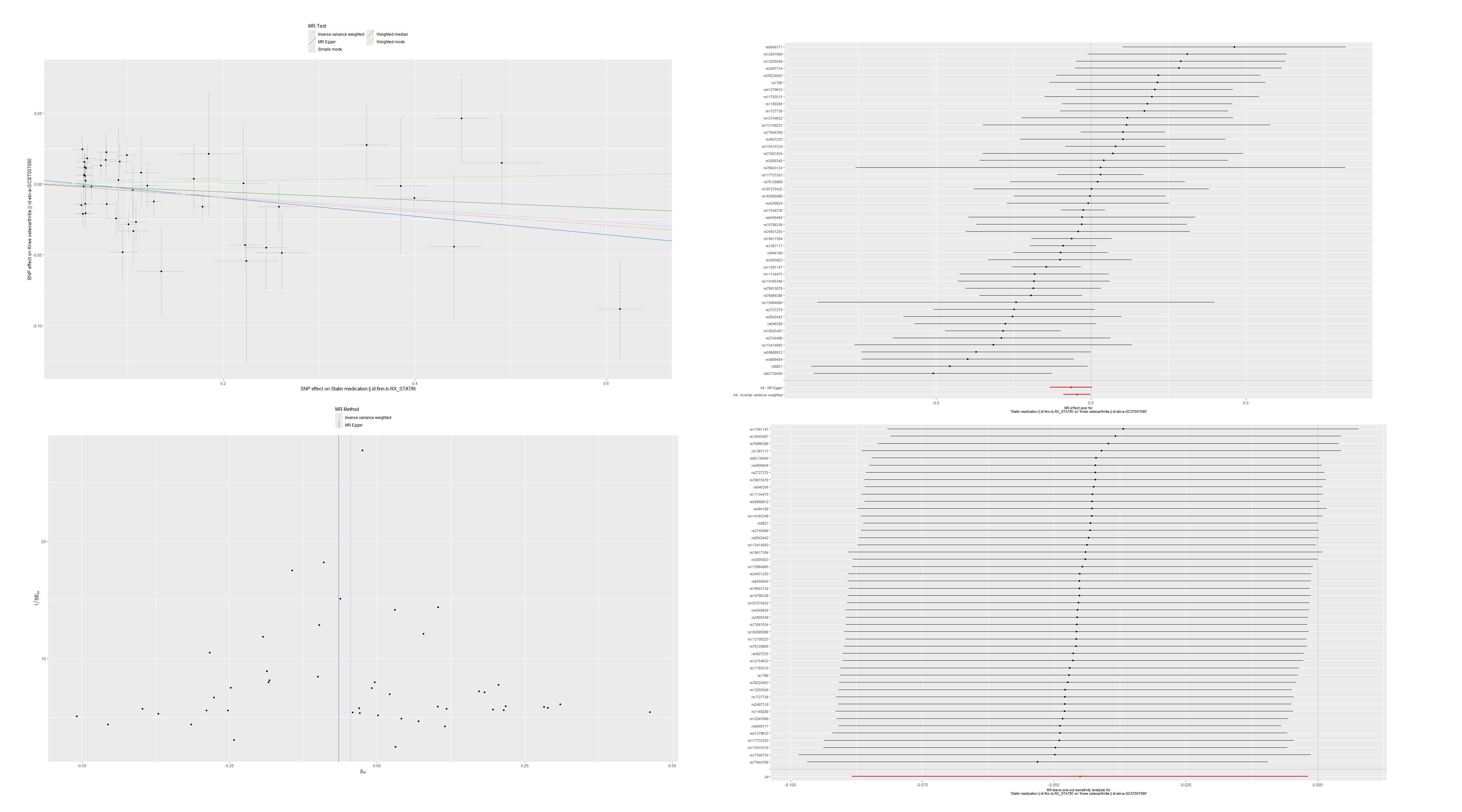 |
| Top left: scatter diagram; Top right: forest map; Bottom left: funnel plot; Bottom right: sensitivity analysis chart of leave one out method |

| **Figure S16. Visualization of mendelian randomization analysis results about drugs used in diabetes (exposure) - knee osteoarthritis (outcome)** |
| --- |
| 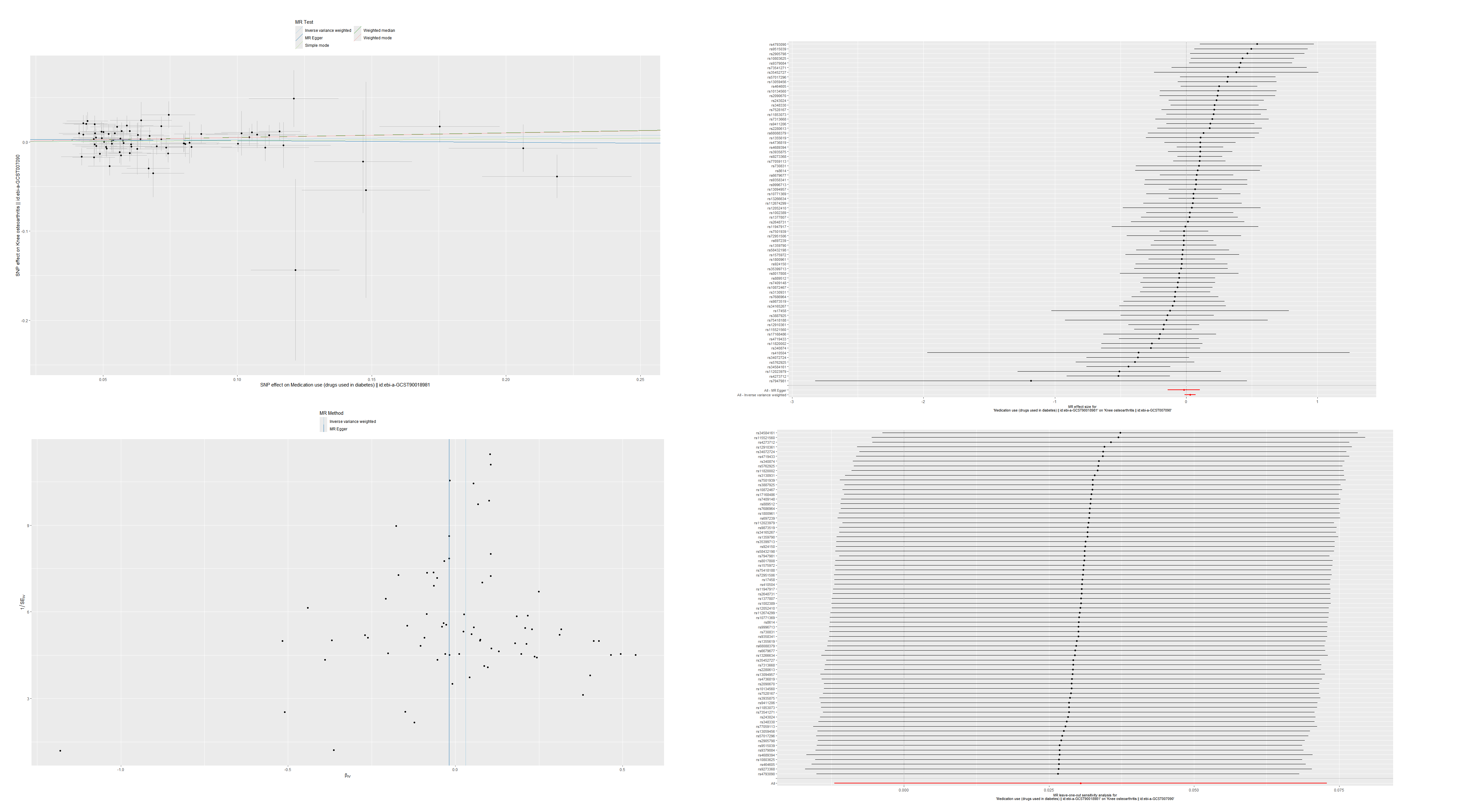 |
| Top left: scatter diagram; Top right: forest map; Bottom left: funnel plot; Bottom right: sensitivity analysis chart of leave one out method |

| **Figure S17. Visualization of mendelian randomization analysis results about thyroid preparations (exposure) - knee osteoarthritis (outcome)** |
| --- |
| 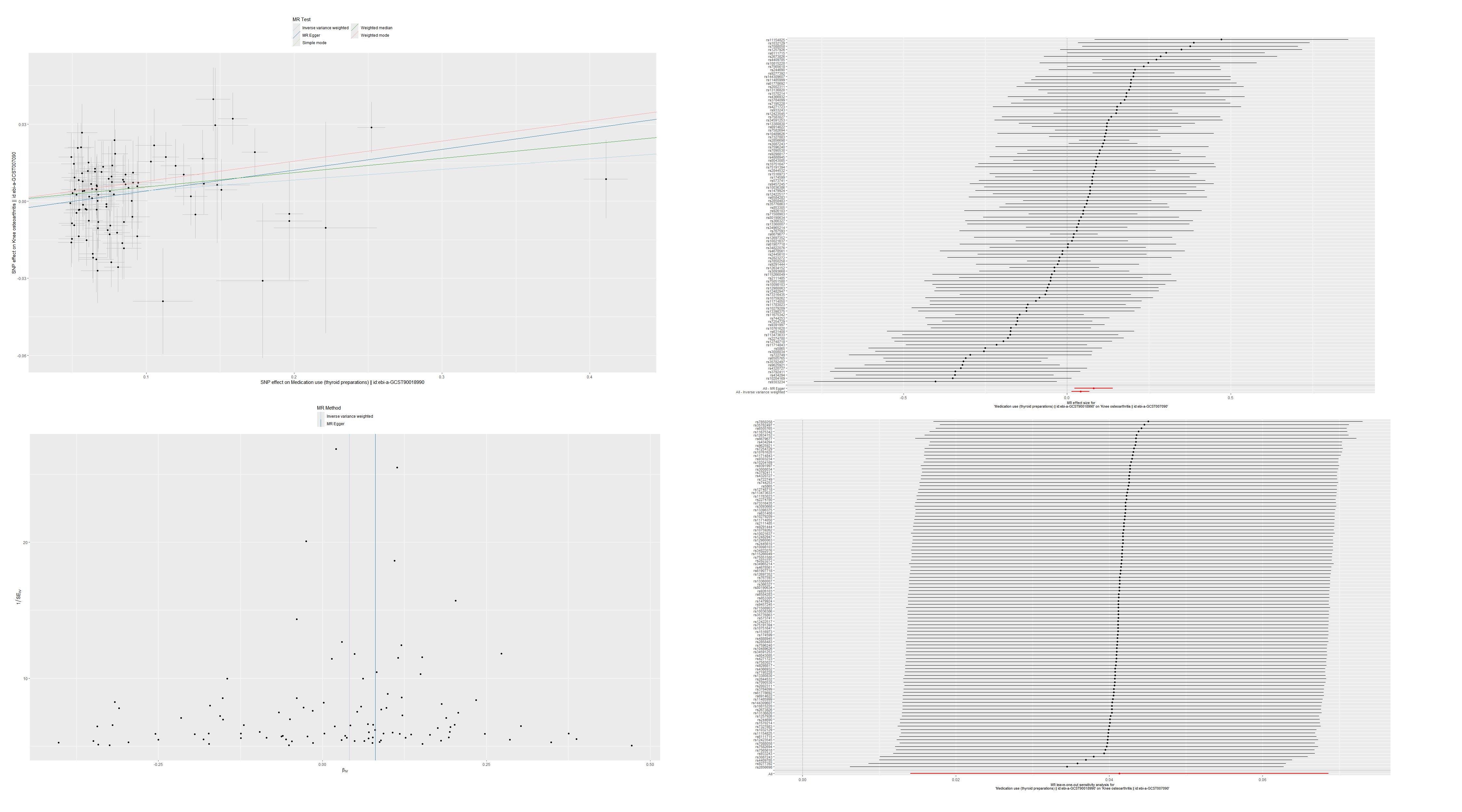 |
| Top left: scatter diagram; Top right: forest map; Bottom left: funnel plot; Bottom right: sensitivity analysis chart of leave one out method |
